# Support Models for Addiction Related Treatment (SMART) for pregnant women: Study protocol of a cluster randomized trial of two treatment models for opioid use disorder in prenatal clinics

**DOI:** 10.1371/journal.pone.0261751

**Published:** 2022-01-13

**Authors:** Ariadna Forray, Amanda Mele, Nancy Byatt, Amalia Londono Tobon, Kathryn Gilstad-Hayden, Karen Hunkle, Suyeon Hong, Heather Lipkind, David A. Fiellin, Katherine Callaghan, Kimberly A. Yonkers

**Affiliations:** 1 Department of Psychiatry, Yale School of Medicine, New Haven, Connecticut, United States of America; 2 Department of Psychiatry, University of Massachusetts School of Medicine, Worcester, Massachusetts, United States of America; 3 Department of Ob/Gyn, University of Massachusetts Medical School, Worcester, Massachusetts, United States of America; 4 Department of Population and Quantitative Health Sciences, University of Massachusetts Medical School, Worcester, Massachusetts, United States of America; 5 Department of Psychiatry and Human Behavior, Brown University, Providence, Rhode Island, United States of America; 6 Department of Obstetrics, Gynecology, and Reproductive Sciences, Yale School of Medicine, New Haven, Connecticut, United States of America; 7 Department of Internal Medicine, Yale School of Medicine, New Haven, Connecticut, United States of America; 8 Department of Emergency Medicine, Yale School of Medicine, New Haven, Connecticut, United States of America; 9 Yale School of Public Health, New Haven, Connecticut, United States of America; Christiana Care/University of Delaware, UNITED STATES

## Abstract

**Introduction:**

The prevalence of opioid use disorder (OUD) in pregnancy increased nearly five-fold over the past decade. Despite this, obstetric providers are less likely to treat pregnant women with medication for OUD than non-obstetric providers (75% vs 91%). A major reason is many obstetricians feel unprepared to prescribe medication for opioid use disorder (MOUD). Education and support may increase prescribing and overall comfort in delivering care for pregnant women with OUD, but optimal models of education and support are yet to be determined.

**Methods and analysis:**

We describe the rationale and conduct of a matched-pair cluster randomized clinical trial to compare the effectiveness of two models of support for reproductive health clinicians to provide care for pregnant and postpartum women with OUD. The primary outcomes of this trial are patient treatment engagement and retention in OUD treatment. This study compares two support models: 1) a collaborative care approach, based upon the Massachusetts Office-Based-Opioid Treatment Model, that provides practice-level training and support to providers and patients through the use of care managers, versus 2) a telesupport approach based on the Project Extension for Community Healthcare Outcomes, a remote education model that provides mentorship, guided practice, and participation in a learning community, via video conferencing.

**Discussion:**

This clustered randomized clinical trial aims to test the effectiveness of two approaches to support practitioners who care for pregnant women with an OUD. The results of this trial will help determine the best model to improve the capacity of obstetrical providers to deliver treatment for OUD in prenatal clinics.

**Trial registration:**

**Clinicaltrials.gov trial registration number**: NCT0424039.

## Introduction

Between 1999 and 2014 the point prevalence of an opioid use disorder (OUD) in pregnant patients increased from 1.5 to 6.5 per 1000 deliveries, which is in line with rates of opioid prescribing in the general population [1]. Thus, it is not surprising that between 2007 and 2016, pregnancy-associated mortality resulting from overdose more than doubled in the US, from 1.3 to 4.2 deaths per 100,000 live births. In many states, overdose is the leading cause of maternal morbidity and mortality [2]. Unfortunately, outpatient providers of medication treatment for OUD (MOUD) are less likely to treat pregnant patients compared to non-pregnant patients (75% vs 91%) [3].

Medication for OUD (MOUD) and behavioral therapy/psychosocial support are recommended in this population [4–10]. Medication treatment enhances adherence to prenatal care and reduces pregnancy and birth complications [11]. The use of MOUD in pregnancy increases the likelihood that a woman will continue treatment after delivery, a period of high risk among perinatal patients with OUD [12]. Unfortunately, it is often difficult for perinatal patients with OUD to access care because of challenges in treatment availability (wait lists, absence of specialized addiction programs, centers that will not enroll pregnant patients) [3,13,14]; accessibility (limited transportation, competing time demands given child care) [15]; affordability (lack of insurance or other financial resources) [16]; and acceptability (concerns over reports to child protective services, stigma/shame related to the illness, provider attitudes toward illness and MOUD) [13,17].

Given the current scope of the opioid crisis in the US, the need for treatment outpaces the capacity to provide it [18]. Obstetrical providers can enhance treatment capacity but report a variety of barriers including lack of expertise and education [19–22], limited physician time and resources [19,23,24], concern about MOUD misuse or diversion [18,25], lack of institutional support [18,23,26], cumbersome regulations [21,27], and insurance barriers (e.g. insufficient rates of reimbursement) [18,19,22,24].

The use of MOUD in perinatal patients is one component of treatment. Psychosocial support and sensitive, respectful approaches to care are also requisite and can enhance retention in treatment and thereby improve maternal and fetal well-being. However, to deliver this level of care, providers need education and training. Unfortunately, little information is available on the best systems of care to provide expertise and support to prenatal care providers and their patients with OUD. To this end we designed a study to compare the effectiveness of two models of support for reproductive health clinicians who provide care for pregnant and postpartum patients with OUD: 1) collaborative care (CC) and 2) Project Extension for Community Healthcare Outcomes (Project ECHO). The CC approach is based upon the Massachusetts Office-Based-Opioid Treatment (OBOT) Model [28] that provides practice-level training and support to providers and patients through the use of care managers (CMs). Project ECHO is a telesupport remote education model that provides mentorship, guided practice, and participation in a learning community, via video conferencing [29]. Both models show feasibility and acceptability in primary care settings but have not been studied in obstetrical settings.

This paper aims to describe the Support Models for Addiction Related Treatment (SMART) trial, a matched pair cluster-randomized clinical trial protocol designed to compare two support models (CC vs. Project ECHO) that provide buprenorphine education and support for providers caring for pregnant patients with OUD.

## Methods and design

The institutional review board (IRB) at all centers participating in this study approved the following study, including Yale University IRB, Lowell General Hospital IRB, Hartford Healthcare IRB, and Beth Israel Deaconess Medical Center IRB. At Yale, the SMART Trial was approved on 1/20/2020 by the Human Research Protection Program Institutional Review Boards, FWA00002571, IRB Protocol ID: 2000027031, submission ID: 200002703. Written informed consent will be obtained from all participants. Study findings will be disseminated through peer-reviewed publications and presentations at scientific conferences.

### Study aims and overview

This study protocol was developed in response to a Patient-Centered Outcomes Research Institute (PCORI) funding initiative addressing the following question: “What is the comparative effectiveness of different strategies for providing support or coordination of services for components of medication-assisted treatment (MAT) (induction and/or psychosocial services) to providers who offer office-based opioid treatment (OBOT) to pregnant women, in terms of maternal and neonatal outcomes?” (https://www.pcori.org/funding-opportunities/announcement/medication-assisted-treatment-cycle-2-2018).

There are two primary aims in this study. First, to determine differences in engagement and retention in OUD treatment (MOUD and/or non-pharmacological care) between patient participants who receive care from a center that uses a CC vs. Project ECHO support model (Aim 1). The second primary aim, is to determine differences in patient activation according to the Patient Activation Measure (PAM). Activation has four stages; “believing the patient role is important, having the confidence and knowledge necessary to take action, actually taking action to maintain and improve one’s health, and staying the course even under stress” [30].

The SMART trial includes twelve obstetric centers in Connecticut (n = 8) and Massachusetts (n = 4) that were randomized to Project ECHO or CC support models modified for perinatal women.

### Study conditions

#### SMART ECHO

Project ECHO (Extension for Community Healthcare Outcomes) was developed originally to build capacity to treat chronic, complex health conditions in rural and underserved communities that lack ready access to clinical specialists [31,32]. It uses a virtual hub-and-spoke educational model that links primary care clinicians with specialists through a real-time learning model made possible by inexpensive videoconferencing technology [31,33]. Unlike traditional telemedicine, the ECHO model results in “force multiplication” [34], where a few specialists mentor many providers, who in turn provide enhanced care for large numbers of patients that would otherwise have limited access to specialty care. Project ECHO is now used to train providers to manage many other conditions, such as HIV [35,36], mental illness [37,38], chronic pain [39,40], diabetes [41], and OUD [33]. Data show it to be an effective and potentially cost-saving model [33]. The Project ECHO Integrated Addictions and Psychiatry (IAP) program trained the largest number of buprenorphine-waivered physicians in rural areas of New Mexico, which started at <20 and increased to 140 as of 2014 [42].

For SMART ECHO, we modified the IAP ECHO to allow expert obstetricians and psychiatrists who regularly treat perinatal women for OUD to train other obstetric providers about OUD screening, diagnosis, and treatment in pregnancy. We collaborated with the Weitzman Institute (https://www.weitzmaninstitute.org/project-echo) to develop SMART ECHO. The Weitzman Institute is a certified “replication center” for Project ECHO, which ensures that all elements of the ECHO sessions are in accordance with the evidence-based approach developed and promoted by the University of New Mexico. Consistent with the traditional ECHO model, we have one hub for all study practices randomized to this condition. A typical SMART ECHO session consists of 1) introductions of participants; 2) a brief didactic session, usually a 30-minute presentation on substance use or mental health; and 3) discussion of case presentations submitted by participants in advance for one hour total. Sessions are twice a month in the first six months and monthly thereafter. Examples of topics covered in these sessions include, management of OUD in pregnancy, pain management and anesthesia at delivery, postpartum management of OUD, neonatal opioid withdrawal syndrome, harm reduction strategies, and psychiatric comorbidities.

#### Collaborative Care for Opioids in Pregnancy (CC-OP)

CC was originally developed to enhance the capacity for treatment of depression in primary care settings [43]. As articulated by the University of Washington, it includes several components [1,31]: systematic screening for medical and behavioral health needs [3,33] and a team-based approach that includes a care manager (CM), psychiatrist and primary care physician [44,45]. The CC model has since been refined [46,47] and tested for treatment of other disorders; it was deployed in additional venues including obstetrical-gynecological settings [48,49]. CC was used in the treatment of OUD in open trials of non-pregnant patients [50,51] and pregnant women [52]. It increased treatment initiation, engagement, and use of psychotherapy among non-pregnant patients [53].

For our CC-OP, we generally follow the Collaborative Opioid Prescribing Model (Massachusetts OBOT Model). However, we are retaining several features of the original CC model that were not in the Massachusetts OBOT Model including the use of a registry. The registry is a secure web-based patient tracking system that is updated twice a month by the CM. Also, we will not limit inclusion to women only selecting buprenorphine treatment. CC-OP includes the following components: universal screening of all pregnant women, a team-based approach where a CM and the obstetrical provider discuss each patient at least once every two-weeks, treating to wellness with regular monitoring via a patient registry, and recovery coaching. Universal screening is done via a validated tool (e.g. the NIDA quick screen, the 4P’s Plus, the SURP-P, etc.), selected by the individual practices and integrated to the individual practice workflow. The CM responsibilities include: 1) screening patients for an OUD; 2) entering CC-OP participants into the patient registry; 3) providing education to participants about OUD; 4) assistance in initiation procedures for patients who would like to receive buprenorphine; 5) informing the obstetrical provider of a positive urine drug screen and need to consider an increase in buprenorphine; if the patient is on methadone they coordinate with the outside treatment program to ensure adequate intervention/support; 6) if the patient is not receiving MOUD, discuss its role and/or psychological treatment; and 7) providing recovery coaching.

### Practice recruitment

Obstetrical clinics of any type, private practices, hospital-based clinics, or Federally Qualified Health Centers, were approached by the study principal investigators. Sites were recruited based on the number of cases of OUD in the geographical area and their ability to be randomized to one or the other condition. The parts of Connecticut and Massachusetts that were of interest were those that had high need. Sites and physicians voluntarily participated. There were no practice eligibility criteria or ineligibility criteria other than their ability to be randomized and willingness to participate.

### Practices and allocation procedures

The unit of randomization was the obstetrical practice. Twelve obstetrical centers were matched into six pairs according to state (4 in Massachusetts and 8 in Connecticut), size, and academic (6) vs non-academic (6), private practices. Most practices were located in smaller urban areas. To randomly assign centers to a model of care, the statistician, masked to the identities of the sites, assigned numbers to each center using a random number generator. Within each matched pair, the clinic assigned the lower randomly generated number was allocated to CC-OP; the remaining clinic was assigned to SMART ECHO for a total of 6 clinics per model of care. The collaborating physicians identify a part-time mid-level clinician, such as a nurse, social worker or research assistant, who is trained to be the CM for CC-OP or data support person for SMART ECHO.

### Patient participant eligibility/ineligibility criteria

Patient participants follow their site randomization. To be eligible, patient-participants must be at least 18 years of age, speak/read English, have a diagnosis of DSM-5 OUD and be less than 34 weeks pregnant at the time of enrollment. Participants are not required to receive or be receiving MOUD at study entry but may be started on MOUD during the field study if they so choose.

### Outcomes

#### Primary outcomes

The first primary outcome is percent engaged which is operationalized as > 2 visits for opioid use disorder treatment in 30 days [53] (>2 visits within 30 days of baseline visit; < = 2 visits since baseline or did not consent to be in the study).

The second component of Aim 1 is retention. We define unsuccessful retention according to a modification of Wilder, et al. (2015) [12], as women who are enrolled in pharmacological or behavioral treatment for opioid use disorder who stopped treatment with no plan for ongoing therapy or medication for > one month (e.g. discharge, relapse and left treatment, lost to follow up with no discharge plan) during pregnancy, and the 3 months post-delivery. We hypothesize that the benefit from a CM and proactive monitoring approach will lead to greater treatment engagement and retention among patient participants.

For Aim 2, our primary outcome is the 13-item Patient Activation Measure (PAM) [30]. The PAM is a patient-centered questionnaire that measures health care knowledge, beliefs, skills, and confidence in managing illnesses, and uses a 4-point Likert scale with higher scores showing more favorable health outcomes [30]. Further details on primary outcomes are listed on Table 1.

**Table 1:** Primary outcome measures.

Outcome	Definition	Assessment Tool	Assessment Timepoint(s)
Treatment engagement	> 2 visits for opioid use disorder treatment within 30 days of baseline	Treatment utilization form, medical records, and study database	30 days after baseline
Treatment retention	No stoppage of OUD treatment (with no plan for ongoing therapy or medication) for > one month	Treatment utilization form, medical records, and study database	Delivery and 3-months postpartum
Patient Activation	Increase in at least one level on the Patient Activation Measure (PAM) from baseline to 34 weeks and 3-months postpartum.	Patient Activation Measure (PAM)	Baseline, 26 weeks, 34 weeks and 3-months postpartum

#### Secondary and exploratory measures and outcomes

**Fig 1:**
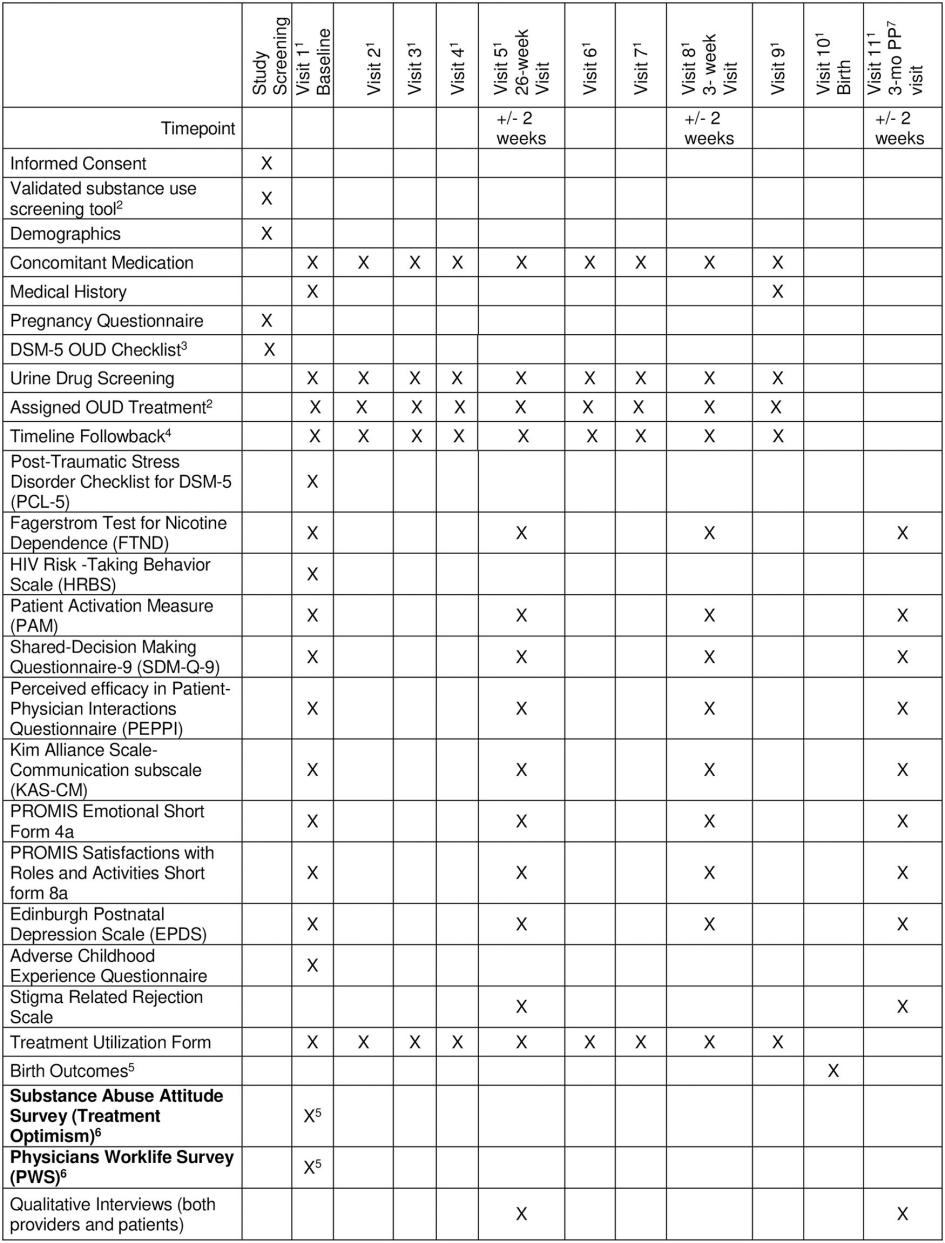
SPIRIT schedule of enrollment, interventions and assessments. 1. Participant will enter the study at different point of pregnancy. Therefore, it is likely that participants will complete varying numbers of visits between screening and the 26-week visit, and the 36-week visit and birth. This is expected and is not considered a protocol deviation. Visit 1 will follow immediately after the consenting process. 2. All participating centers will administer a validated substance use screening tool, such as the NIDA Quick Screen, 4Ps Plus or equivalent validated instrument as standard of care. The tool utilized is determined by the clinical site. 3. The DSM-5 OUD Checklist will be administered to any patients who have a positive substance use screen as part of the standard of care. 4. The Timeline Followback (TLFB) will be conducted monthly during the length of the study. The number of TLFB assessments will vary depending on when the patient enters the study and when they give birth. 5. Birth outcomes include: Low birth weight, resuscitation at delivery, fetal demise, preterm birth, duration of hospitalization. **6. Provider measures**: The Substance Abuse Attitude Scale, the Physicians Worklife Survey and the Qualitative Interviews will be completed by providers and done prior to the first participant being enrolled at the site and after the last participant is enrolled at the site. 7. **3**-mo PP: 3-months postpartum.

We will collect secondary and exploratory process and outcomes measures that are outlined in Table 2 and Fig 1. Secondary and exploratory analyses for Aim 1 will compare eligible women in the two conditions (gravidas with an OUD, not in treatment when presenting for prenatal care) on the following outcomes: 1) #/% offered MOUD including buprenorphine, specifically; 2) #/% initiated onto MOUD; 3) for all participants, #/% retained on MOUD and 4) rates of abstinence from illicit opioids or misuse of prescription opioids [54]. Additionally, for all pregnant women treated with MOUD: 1) #/% continuing MOUD at 3 months postpartum; 2) #/% engaging in an opioid treatment program at 3 months post-delivery; 3) #/% with concurrent substance use according to the Timeline Followback (TLFB) [54] and urine tests; 4) fetal and neonatal outcomes (low birth weight, resuscitation at delivery, fetal demise, preterm birth, duration of hospitalization); 5) racial and ethnic differences; and 6) differences among participants who use illicit street opioids (e.g. heroin) vs. those that use prescription opioids.

**Table 2:** Secondary and exploratory outcomes.

Outcome	Definition	Assessment Tool	Assessment Timepoints	Type of Outcome
Process Measures				
Initiated onto medication for opioid use disorder (MOUD)	> 2 visits for MOUD treatment within 30 days of baseline	Treatment utilization form (TUF), medical records, and study database	30 days after baseline	Secondary
Retained on MOUD	No stoppage of MOUD treatment (with no plan for ongoing therapy or medication) for > one month	TUF, medical records, and study database	Delivery and 3-months postpartum	Secondary
Offered MOUD	Healthcare provider discussed MOUD with patient	Patient self-report at baseline and monthly research surveys via TUF	30 days after baseline	Exploratory
Postpartum Engagement in treatment	Engaged in an opioid treatment program at 3-months postpartum	TUF, medical records, and study database	Delivery and 3-months postpartum	Exploratory
Abstinent from illicit opioids or misuse or prescription opioids	No self-reported use and negative drug screen	a. Timeline Followback (TLFB) b. Urine drug screens	a. monthly assessments b. each prenatal and post-delivery office visit	Exploratory
Concurrent substance use	Self-reported use and/or positive urine drug screen	a. TLFB b. Urine drug screens	a. monthly assessments b. each prenatal and post-delivery office visit	Exploratory
Patient Measures				
Stigma	Scale score	Stigma-Related Rejection Scale (SRS)	Week 26 of pregnancy and 3-months postpartum	Secondary
Shared Decision Making	Scale score	SDM-Q-9	Baseline and week 26 and 36 of pregnancy	Exploratory
Patient-Physician Interaction	Scale score	PEPPI-5	Baseline, week 26 and 36 of pregnancy, and 3-months postpartum	Exploratory
Clinician/Patient therapeutic relationship	Scale score	Kim Alliance Communication (KAC)	Baseline, week 26 and 36 of pregnancy, and 3-months postpartum	Exploratory
Satisfaction with roles and activities	Scale score	PROMIS Emotional Short Form 4a	Baseline, week 26 and 36 of pregnancy, and 3-months postpartum	Exploratory
Depression	Scale score	EPDS	Baseline, week 26 and 36 of pregnancy, and 3-months postpartum	Exploratory
Practitioner Measures				
Physician Work Satisfaction	Scale score	Physician Work-Life Survey	Before first participant enrollment and after last participant enrollment	Secondary
Attitude Toward Treatment of Individuals with Substance Misuse	Scale score	Substance Abuse Attitude Survey (SAAS)	Before first participant enrollment and after last participant enrollment	Secondary
Birth Outcomes				
Birth weight	Weight in grams at birth	Medical records	Birth	Secondary
Low birth weight	<2500 grams	Medical records	Birth	Exploratory
Resuscitation at delivery	Any respiratory assistance at birth: suctioning, positive pressure ventilation via bag/mask, endotracheal intubation, chest compression, epinephrine/volume administration	Medical records	Birth	Exploratory
Fetal demise	Intrauterine fetal demise after 20 weeks’ gestation and/or 350 grams birthweight	Medical records	Birth	Exploratory
Preterm birth	Born before 37 weeks’ gestation	Medical records	Birth	Exploratory
Duration of hospitalization	Discharge date- admittance date	Medical records	Hospital discharge	Exploratory

Note: SDM-Q-9 = 9-item Shared Decision Making Questionnaire; PEPPI-5 = 5-item Perceived Efficacy in Patient-Physician Interactions; PROMIS = Patient-Reported Outcomes Measurement Information System; EPDS = Edinburgh Postnatal Depression Scale.

Secondary and exploratory outcomes for Aim 2 include participant reported differences in: 1) the Shared-Decision Making Questionnaire-9 (SDM-Q-9) [55]; 2) Perceived efficacy in Patient-Physician Interactions Questionnaire (PEPPI) [56]; 3) Kim Alliance Scale-Communication subscale (KAS-CM) [57]; 4) PROMIS Emotional Short Form and Satisfaction with Roles and Activities [58]; and 5) Edinburgh Postnatal Depression Scale (EPDS) [59,60]; 6) Stigma-Related Rejection Scale (SRS) [61]. We will also explore racial and ethnic differences in patient measures and possible differences in outcomes among those who use illicit street opioids vs misuse of prescription opioids.

Providers complete the following secondary outcomes: the Autonomy (5 items: e.g. freedom in practice activities), Patient Care Issues (4 items: e.g. perception of needs of patients), and Relationship with Patients (4 items: e.g., satisfaction with patient relationship) subscales from the Physicians Worklife Survey (PWS) [62] and the Treatment Optimism Subscale of the Substance Abuse Attitude Survey (SAAS (4 items)) [63] that measures attitudes toward treatment of individuals with substance use.

### Fidelity procedures

#### CC-OP

To ensure that CC-OP is being implemented with fidelity we will:

1. Check screening rates for an OUD by conducting medical chart reviews of at least 50 consecutive charts (selected to start at a random date) at each CC-OP site at baseline, 6 months, 12 months, 18 months, 24 months, and 36 months. Screening is considered completed if patients were asked about substance use using a validated scale and documented in the medical record. The percentage of women being screened for OUD at their first prenatal visit is calculated.
2. Measure CM and/or physician contacts the patient (either in-person, by phone or via telehealth) and discuss each their progress with OUD treatment at least biweekly. This is tracked in the participant registry, which is audited quarterly by the coordinating centers and is a measure of “team-based care.”
3. Follow the “treat to wellness” model. If a participant’s urine drug screen (UDS) is negative for a prescribed MOUD, or if the UDS is positive for illicit or non-prescribed opioids, action will be taken by the providers (e.g., increase buprenorphine dose, initiate or extend recovery coaching or refer to additional substance use treatment). Actions are noted in the registry and audited quarterly. We give CC-OP sites a “report card” every 6 months that provides feedback on adherence to the fidelity metrics.

#### SMART ECHO

To ensure fidelity to the Project ECHO model we will follow the Weitzman Institute’s replication model [64]:

1. Use a web-based database to monitor outcomes. Physician participant and session details will be tracked in the UNM-required iECHO platform. Physician participants will complete an enrollment form on Survey Monkey before the first SMART ECHO session to capture demographic information, session topic ideas, pre-intervention self-reported measures of attitudes, self-efficacy, behavior related to screening, prescribing, and interdisciplinary care. A program specialist will maintain an attendance tracker for all sessions, which will be completed after each session. Physician participants will complete a CME survey at the end of each session, as well as mid-series and end-of-series surveys to capture: overall satisfaction with SMART ECHO, session topic ideas, self-reported measures of attitudes, self-efficacy.
2. Follow a case-based learning format. The majority of each SMART ECHO session is dedicated to at least 30 minutes of case discussion. Participants are provided a case feedback form inquiring about their perceptions of the quality of the recommendations they received and the likelihood that they will use the recommendations provided. The content is monitored according to: # of unique cases presented, # of cases presented, and # (%) of participants who submitted cases.
3. Leverage technology and utilize Zoom videoconferencing and leverage its advanced features, including polling and chat, to promote engagement during SMART Project ECHO sessions.
4. SMART ECHO team uses the Project ECHO Quality Checklist, which assesses the quality of the following domains: technology, remote faculty technology, session logistics, faculty, participants, and HIPAA Compliance. This checklist is completed during each ECHO session and allows the measurement of the % of ECHO sessions that satisfied all of the quality requirements of a Project ECHO session.

### Study procedures

Patients who screen positive for substance use and have OUD according to DSM-5 criteria will be informed about the study and if interested, site staff will obtain consent for further eligibility determination. After providing informed consent, patient-participants will complete a screening assessment that includes demographic information, pregnancy dates, a checklist that confirms an OUD diagnosis, and verification of other inclusion criteria and lack of exclusion criteria. If consent is not provided, only basic demographic information, number of weeks pregnant and the reason for disinterest, if known, is collected. Data will be collected on a secure tablet computer or via a secure email link using REDCap, a web application for building and managing online surveys and databases. A study team member will review and verify screening results to confirm eligibility. Participants will be compensated with an Amazon gift card for the time they spent during the screening process. Enrollees will complete additional baseline questionnaires and submit a urine sample for a UDS during Visit 1 and are compensated with an additional gift card for their time.

**Fig 2:**
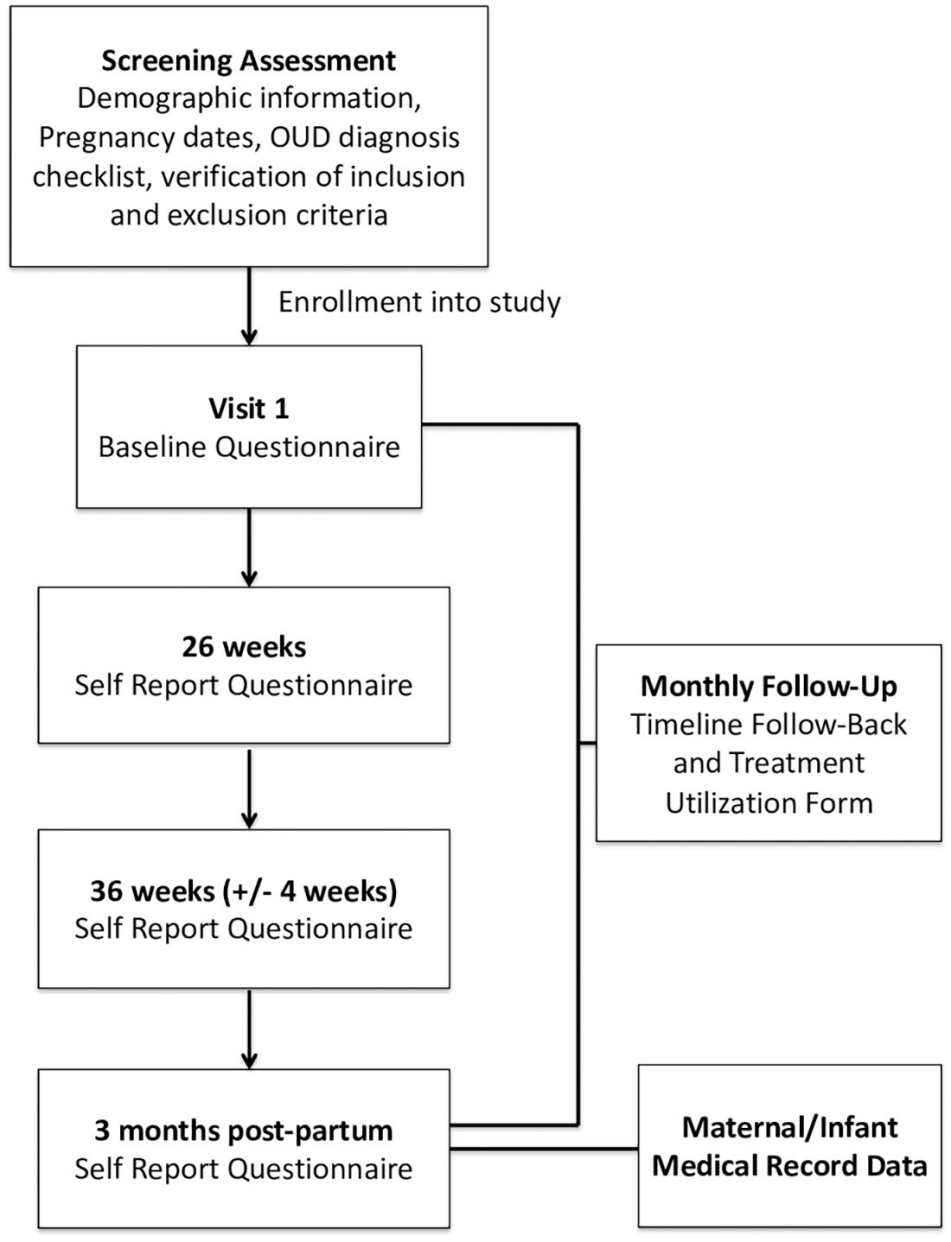
Participant flow through study procedures. Note: If a patient-participant is enrolled after 26 weeks’ gestation and before 36 weeks’ gestation, the items from the 26-week visit are added to the baseline assessment.

After completion of the baseline portion, patient-participants will be contacted monthly by a research assistant blinded to the patient’s clinic or study arm. They will collect the Timeline Follow-Back (TLFB) [65] and the Treatment Utilization Form that includes questions about the quantity and frequency of all substances used and any treatment received outside of the study. Patient participants will complete additional self-report questionnaires at baseline, 26 weeks and 36 weeks (+/- 4 weeks), and at 3-months post-partum. Involvement of child protective services is assessed in the 3-month postpartum questionnaire. Maternal/infant medical record data will be extracted by the onsite staff for maternal, fetal, and neonatal outcomes (Fig 2).

### Analytic plan, power, and sample size estimate

Descriptive statistics will summarize data on all randomized subjects by treatment group, and also by site. Data analyses will employ the intent-to-treat principle and include follow-up on all patient-participants regardless of their retention to treatment.

To test for differences between care models for the primary outcomes, we will use permutation tests [66,67] that account for the cluster-randomized nature of the trial. We will test for differences in proportions (for binary outcomes) and in means (for quantitative outcomes) between the two interventions. The permutation approach will also be used to construct 95% confidence intervals for those differences.

To evaluate the different effects associated with implementation of each model, we will also report estimates of proportion and mean differences within each matched pair together with 95% confidence intervals. Due to feasibility constraints (only 12 groups in 6 matched pairs), we will not be able to perform a rigorous statistical evaluation of the effects of site-level factors. However, we will present descriptive statistics, by site, that may be useful in guiding future use of the proposed models.

In addition, we will perform exploratory mixed model analyses to compare each model’s outcomes due to individual-level covariates including age, race/ethnicity, parity, illicit substances vs non-medical use of prescription opioids, and concurrent other substance use. Mixed effects models will account for potential positive correlations among observations within sites and within matched pairs and will evaluate fixed effects for the interventions and potential covariates at the individual level. Generalized linear mixed models will be used for categorical outcomes and linear mixed models for continuous outcomes.

The proposed sites are primarily in high-need, urban settings. Combined, the sites in our study have an average of 36,995 deliveries per year. Based on the rates of OUD in pregnant women in MA (13.1/1000) and CT (10/1000) this would mean an average of 430 pregnant women per year with an OUD would be served by these locations for a total of 1,075 eligible women (or 90 per site) over the 2.5 years of study recruitment.

We used the approach of Hayes and Bennett (1999) [68] for matched-pair cluster randomized trials to conduct power analyses for our three primary dichotomous outcomes. For the treatment retention outcome, we hypothesized that the proportion retained in CC-OP would be greater compared to ECHO. A sample of six cluster pairs (12 clusters) with 40 participants per cluster achieves 80% power to detect a difference of 0.20 between the CC proportion engaged in treatment (0.94) [52] and the ECHO proportion (0.74). This is based on a two-sided paired test of the proportion difference with a significance level of 0.05 and an estimated within-pair coefficient of variation (CV) of 0.10 between clusters. We did not have empirical data for an estimate of the CV, so we followed the approach of Hayes and Bennett (1999) [68]. We generated 10,000 data sets with 6 clusters each and calculated the CV based on formula(9) in the manuscript. This follows the conservative approach suggested on p.322 of the paper (i.e. to use the coefficient of variation between clusters within each group as an upper limit for the coefficient of variation in matched pairs designs). After examining the distribution of the resulting coefficient of variation (CV) we selected 0.10 as the value for our subsequent power calculations since more than 90% of the data sets had values under that threshold.

For the treatment engagement outcome, we hypothesized that the proportion engaged in CC-OP would be greater compared to ECHO. Assuming 90 eligible participants per cluster with six clusters per group and a 43% engagement rate in the CC-OP group [53], we are powered at 80% to detect a 13% difference between CC-OP and SMART ECHO in engagement rates, setting significance level at 0.05 and CV at 0.10.

In an ongoing study of mothers with depression randomized to cognitive behavioral therapy with or without a community ambassador, 37% of 103 participants increased their patient activation by at least one PAM level (M. Smith, 2019, personal communication). Using this proportion for the CC-OP group and hypothesizing that participants in the CC-OP are more likely than ECHO participants to see an increase in patient activation, 40 participants per site will provide 80% power to detect a clinically meaningful 15% difference in the proportion of women who increase at least one PAM level between CC-OP and SMART ECHO. This estimate assumes a two-sided test, with a significance level of 0.05 and an estimated within-pair coefficient of variation (CV) of 0.10 between clusters.

### Provider and qualitative interviews

We will use qualitative methods to understand the experiences of obstetric care providers. We will invite at least one provider from each practice (minimum of 12) to participate in qualitative interviews to assess their perceptions and the impact of the care models to which they were assigned. A subset of patient participants (1–2 per site) will be given the opportunity to participate in qualitative interviews to collect data on patient satisfaction with treatment of OUD. Interviews will be transcribed by a member of the research team and de-identified. Further details of the qualitative work will be published in a forthcoming separate manuscript.

## Discussion

Improving the capacity of obstetric caregivers to provide treatment for pregnant patients with an OUD is critical. While addiction treatment admissions among pregnant patients with OUD increased over the past few years [69–71], the use of MOUD remains low [70,71], and the consequences are significant with opioid overdose becoming a leading cause of maternal mortality [2]. Barriers to treatment access are well documented [3,15], including a limited number of providers who offer and feel comfortable providing treatment for OUD in pregnancy. Many obstetricians who would like to offer comprehensive care to pregnant patients with OUD have not been given the knowledge base and support required to treat these patients. Addressing these barriers by providing education, training, and support can improve care for pregnant patients with OUD [15,52,72,73]. The Project SMART trial aims to address these limitations by testing the effectiveness of two approaches to support practitioners who care for women with OUD in obstetric settings.

Despite the potential benefits of a program that provides integrated prenatal and addiction care, there are a limited number of studies that compare the effectiveness of models for supporting obstetric settings. Only a few studies assessed possible differences in maternal and fetal well-being for gravidas treated in programs that provided combined vs. separate prenatal care and addiction treatment [72]. Some cohort studies provided information on fetal outcomes in centers that provide combined care [73,74]. The results of this study can inform how we can better address OUD in obstetric settings and help address maternal mortality due to OUD and the issue of lack of care addressing social determinants of health. Equally important is the lack of knowledge regarding women’s experiences when they receive treatment. This trial can provide information on patient’s engagement in care, knowledge of care, confidence in managing pregnancy and their addiction, alliance with their providers, and emotional well-being, and fill in these important gaps.

The comparator conditions, SMART ECHO and CC-OP have different strengths and weaknesses. SMART ECHO is scalable, and with the development of a “learning community” through the group case presentation process, it is a very powerful way in which to share knowledge and enhance therapeutic confidence among providers. The benefits of the “learning community” can go beyond clinical issues of medication initiation and titration to include ways to improve referral systems. Depending upon the commitment of the practice and their willingness to share resources, multiple individuals—such as care providers and support staff—can be trained through the SMART ECHO model. However, members of the practice who participate in SMART ECHO, must make time and be available to attend the SMART ECHO sessions and to share case information with their colleagues. Availability can be a challenge in busy obstetric settings not only because of issues of patient flow but because of obstetrical emergencies. Additionally, physicians or advanced practice nurses must fit their treatment of OUD in practices that are already stressed for time.

Compared to SMART ECHO, CC-OP is advantageous in that its use of a CM may help to save physicians’ time. The CM is trained in supporting behavioral techniques such as recovery coaching. The limitation of CC-OP is that it is potentially more labor-intensive—the CM must be willing to follow a group of patients rather closely and this can take time and effort. CC-OP can also be more costly than SMART ECHO; for the model to work, practices must be willing to dedicate financial resources to the CM. However, Medicare has made the costs of a CM billable and Massachusetts Medicaid has started to allow providers to bill for recovery coaching. Unfortunately, Medicaid reimbursement for recovery coaching is not universal and currently is not in place in Connecticut; a study such as this one could inform policymakers in funding these services.

The results of this Project SMART Trial may have policy and public health implications. Both the SMART ECHO and CC-OP interventions have the potential to be feasible, sustainable, and transportable to practice settings besides obstetric care. Measures of physicians’ attitudes, perception of support, and perception of the relationship with patients will also provide data to understand clinicians’ needs and potential targets for interventions. Lastly, the outcomes of this study may be helpful to payers and providers to understand how to implement adequate and manageable support systems. Patients, clinicians, payers, and policymakers will benefit from a comparison of these two models as we seek to understand if retention and engagement of pregnant women with opioid use disorder are impacted by the education and support their providers receive and the type of care patients receive during and after pregnancy.

## Supporting information

S1 Checklist Standard Protocol Items: Recommendations for Interventional Trials (SPIRIT) checklist. (DOC)

S1 File Approved Yale University IRB protocol. (PDF)

S2 File Approved Yale University IRB consent form. (PDF)

## References

[1] Haight SC, Ko JY, Tong VT, Bohm MK, Callaghan WM. Opioid Use Disorder Documented at Delivery Hospitalization—United States, 1999–2014. Morbidity and Mortality Weekly Report. 2018;67(31):845–9. doi: 10.15585/mmwr.mm6731a1 30091969 PMC6089335

[2] Cleveland LM, McGlothen-Bell K, Scott LA, Recto P. A life-course theory exploration of opioid-related maternal mortality in the United States. Addiction. 2020;115(11):2079–88. doi: 10.1111/add.15054 32279394 PMC7587012

[3] Patrick SW, Buntin MB, Martin PR, Scott TA, Dupont W, Richards M, et al. Barriers to Accessing Treatment for Pregnant Women with Opioid Use Disorder in Appalachian States. Subst Abus. 2018:1–18. Epub 2018/06/28. 29949454 10.1080/08897077.2018.1488336 PMC9069995

[4] Kampman K, Comer S, Cunningham CO, Fishman MJ, Gordon A, Langleben D, et al. National Practice Guideline for the Use of Medications in the Treatment of Addiction Involving Opioid Use. Chevy Chase, MD: American Society of Addiction Medicine, 2015.

[5] ACOG. Opioid use and opioid use disorder in pregnancy. Committee Opinion No.711. Obstetrics and gynecology. 2017;130:e81–94. doi: 10.1097/AOG.0000000000002235 28742676

[6] Jones HE. Treating opioid use disorders during pregnancy: historical, current, and future directions. Subst Abus. 2013;34(2):89–91. Epub 2013/04/13. doi: 10.1080/08897077.2012.752779 . 23577898

[7] Substance Abuse and Mental Health Services Administration. A Collaborative Approach to the Treatment of Pregnant Women with Opioid Use Disorders. Rockville, MD: Substance Abuse and Mental Health Services Administration; 2016.

[8] Hand DJ, Short VL, Abatemarco DJ. Treatments for opioid use disorder among pregnant and reproductive-aged women. Fertility and Sterility. 2017;108(2):222–7. doi: 10.1016/j.fertnstert.2017.06.011 28697916

[9] Fullerton CA, Kim M, Thomas CP, Lyman DR, Montejano LB, Dougherty RH, et al. Medication-assisted treatment with methadone: assessing the evidence. Psychiatr Serv. 2014;65(2):146–57. Epub 2013/11/20. doi: 10.1176/appi.ps.201300235 . 24248468

[10] Klaman SL, Isaacs K, Leopold A, Perpich J, Hayashi S, Vender J, et al. Treating Women Who Are Pregnant and Parenting for Opioid Use Disorder and the Concurrent Care of Their Infants and Children: Literature Review to Support National Guidance. J Addict Med. 2017;11(3):178–90. Epub 2017/04/14. doi: 10.1097/ADM.0000000000000308 . 28406856 PMC5457836

[11] Jones HE, O’Grady KE, Malfi D, Tuten M. Methadone maintenance vs. methadone taper during pregnancy: maternal and neonatal outcomes. The American journal on addictions/American Academy of Psychiatrists in Alcoholism and Addictions. 2008;17(5):372–86. doi: 10.1080/10550490802266276 . 18770079

[12] Wilder C, Lewis D, Winhusen T. Medication assisted treatment discontinuation in pregnant and postpartum women with opioid use disorder. Drug & Alcohol Dependence. 2015;149:225–31. doi: 10.1016/j.drugalcdep.2015.02.012 25735465

[13] Meyer M, Phillips J. Caring for pregnant opioid abusers in Vermont: A potential model for non-urban areas. Preventive Medicine. 2015;80:18–22. doi: 10.1016/j.ypmed.2015.07.015 26212632 PMC4592470

[14] Jumah NA, Edwards C, Balfour-Boehm J, Loewen K, Dooley J, Gerber Finn L, et al. Observational study of the safety of buprenorphine+naloxone in pregnancy in a rural and remote population. BMJ Open. 2016;6(10):e011774. Epub 2016/11/02. doi: 10.1136/bmjopen-2016-011774 . 27799240 PMC5093362

[15] Sutter MB. Patient-centered Care to Address Barriers for Pregnant Women with Opioid Dependence. Obstetrics and gynecology clinics of North America. 2017;44(1):95–107. doi: 10.1016/j.ogc.2016.11.004 28160896

[16] Bachhuber MA, Mehta PK, Faherty LJ, Saloner B. Medicaid Coverage of Methadone Maintenance and the Use of Opioid Agonist Therapy Among Pregnant Women in Specialty Treatment. Med Care. 2017;55(12):985–90. Epub 2017/11/15. doi: 10.1097/MLR.0000000000000803 . 29135769 PMC5772976

[17] Falletta L, Hamilton K, Fischbein R, Aultman J, Kinney B, Kenne D. Perceptions of child protective services among pregnant or recently pregnant, opioid-using women in substance abuse treatment. Child Abuse Negl. 2018;79:125–35. Epub 2018/02/13. doi: 10.1016/j.chiabu.2018.01.026 . 29433069

[18] Huhn AS, Dunn KE. Why aren’t physicians prescribing more buprenorphine? Journal of Substance Abuse Treatment. 2017;78:1–7. doi: 10.1016/j.jsat.2017.04.005 28554597 PMC5524453

[19] Barry DT, Irwin KS, Jones ES, Becker WC, Tetrault JM, Sullivan LE, et al. Integrating Buprenorphine Treatment into Office-based Practice: a Qualitative Study. Journal of general internal medicine. 2009;24(2):218–25. doi: 10.1007/s11606-008-0881-9 19089500 PMC2628993

[20] Gordon AJ, Kavanagh G, Krumm M, Ramgopal R, Paidisetty S, Aghevli M, et al. Facilitators and barriers in implementing buprenorphine in the Veterans Health Administration. Psychology of Addictive Behaviors. 2011;25(2):215–24. doi: 10.1037/a0022776 21480679

[21] DeFlavio JR, Rolin SA, Nordstrom BR, Kazal LA Jr. Analysis of barriers to adoption of buprenorphine maintenance therapy by family physicians. Rural Remote Health. 2015;15:3019. Epub 2015/02/05. . 25651434

[22] McMurphy S, Shea J, Switzer J, Turner BJ. Clinic-based Treatment for Opioid Dependence: A Qualitative Inquiry. American journal of health behavior. 2006;30(5):544–54. doi: 10.5555/ajhb.2006.30.5.544 16893317

[23] Hutchinson E, Catlin M, Andrilla CHA, Baldwin L-M, Rosenblatt RA. Barriers to Primary Care Physicians Prescribing Buprenorphine. The Annals of Family Medicine. 2014;12(2):128–33. doi: 10.1370/afm.1595 24615308 PMC3948759

[24] Kermack A, Flannery M, Tofighi B, McNeely J, Lee JD. Buprenorphine prescribing practice trends and attitudes among New York providers. Journal of Substance Abuse Treatment. 2017;74:1–6. doi: 10.1016/j.jsat.2016.10.005 28132694

[25] McRae-Clark AL, Carter RE, Price KL, Baker NL, Thomas S, Saladin ME, et al. Stress- and cue-elicited craving and reactivity in marijuana-dependent individuals. Psychopharmacology. 2011;218(1):49–58. doi: 10.1007/s00213-011-2376-3 21710170 PMC3209966

[26] Walley AY, Alperen JK, Cheng DM, Botticelli M, Castro-Donlan C, Samet JH, et al. Office-Based Management of Opioid Dependence with Buprenorphine: Clinical Practices and Barriers. Journal of general internal medicine. 2008;23(9):1393–8. doi: 10.1007/s11606-008-0686-x 18592319 PMC2518016

[27] Andraka-Christou B, Capone MJ. A qualitative study comparing physician-reported barriers to treating addiction using buprenorphine and extended-release naltrexone in U.S. office-based practices. International Journal of Drug Policy. 2018;54:9–17. doi: 10.1016/j.drugpo.2017.11.021 29324253

[28] LaBelle CT, Han SC, Bergeron A, Samet JH. Office-Based Opioid Treatment with Buprenorphine (OBOT-B): Statewide Implementation of the Massachusetts Collaborative Care Model in Community Health Centers. Journal of Substance Abuse Treatment. 2016;60:6–13. doi: 10.1016/j.jsat.2015.06.010 26233698 PMC4682362

[29] Arora S, Thornton K, Jenkusky SM, Parish B, Scaletti JV. Project ECHO: Linking university specialists with rural and prison-based clinicians to improve care for people with chronic hepatitis C in New Mexico. Public health reports. 2007;122(SUPPL. 2):74–7. doi: 10.1177/00333549071220S214 17542458 PMC1831800

[30] Hibbard JH, Mahoney ER, Stockard J, Tusler M. Development and Testing of a Short Form of the Patient Activation Measure. Health Services Research. 2005;40(6p1):1918–30. doi: 10.1111/j.1475-6773.2005.00438.x 16336556 PMC1361231

[31] Arora S, Geppert CMA, Kalishman S, Dion D, Pullara F, Bjeletich B, et al. Academic health center management of chronic diseases through knowledge networks: Project ECHO. Academic Medicine. 2007;82(2):154–60. doi: 10.1097/ACM.0b013e31802d8f68 17264693 PMC3855463

[32] Arora S, Thornton K, Komaromy M, Kalishman S, Katzman J, Duhigg D. Demonopolizing Medical Knowledge. Academic Medicine. 2014;89(1):30–2. doi: 10.1097/ACM.0000000000000051 24280860

[33] Zhou C, Crawford A, Serhal E, Kurdyak P, Sockalingam S. The Impact of Project ECHO on Participant and Patient Outcomes: A Systematic Review. Academic medicine: journal of the Association of American Medical Colleges. 2016;91(10):1439–61. Epub 2016/08/05. doi: 10.1097/ACM.0000000000001328 . 27489018

[34] Komaromy M, Bartlett J, Manis K, Arora S. Enhanced Primary Care Treatment of Behavioral Disorders With ECHO Case-Based Learning. Psychiatric Services. 2017;68(9):873–5. doi: 10.1176/appi.ps.201600471 28806893

[35] Moeckli J, Stewart KR, Ono S, Alexander B, Goss T, Maier M, et al. Mixed-Methods Study of Uptake of the Extension for Community Health Outcomes (ECHO) Telemedicine Model for Rural Veterans With HIV. J Rural Health. 2017;33(3):323–31. Epub 2016/08/25. doi: 10.1111/jrh.12200 . 27557039

[36] Rich KM, Bia J, Altice FL, Feinberg J. Integrated Models of Care for Individuals with Opioid Use Disorder: How Do We Prevent HIV and HCV? Curr HIV/AIDS Rep. 2018;15(3):266–75. Epub 2018/05/19. doi: 10.1007/s11904-018-0396-x . 29774442 PMC6003996

[37] Mazurek MO, Brown R, Curran A, Sohl K. ECHO Autism: A New Model for Training Primary Care Providers in Best-Practice Care for Children with Autism. Clinical Pediatrics. 2017;56(3):247–56. doi: 10.1177/0009922816648288 27169714

[38] Sockalingam S, Arena A, Serhal E, Mohri L, Alloo J, Crawford A. Building Provincial Mental Health Capacity in Primary Care: An Evaluation of a Project ECHO Mental Health Program. Acad Psychiatry. 2018;42(4):451–7. Epub 2017/06/09. doi: 10.1007/s40596-017-0735-z . 28593537

[39] Carey EP, Frank JW, Kerns RD, Michael Ho P, Kirsh SR. Implementation of telementoring for pain management in Veterans Health Administration: Spatial analysis. Journal of Rehabilitation Research and Development. 2016;53(1):147–56. doi: 10.1682/JRRD.2014.10.0247 26934696

[40] Katzman JG, Comerci G, Boyle JF, Duhigg D, Shelley B, Olivas C, et al. Innovative telementoring for pain management: Project ECHO pain. Journal of Continuing Education in the Health Professions. 2014;34(1):68–75. doi: 10.1002/chp.21210 24648365

[41] Colleran K, Harding E, Kipp BJ, Zurawski A, MacMillan B, Jelinkova L, et al. Building Capacity to Reduce Disparities in Diabetes:Training Community Health Workers Using an Integrated Distance Learning Model. The Diabetes Educator. 2012;38(3):386–96. doi: 10.1177/0145721712441523 . 22491397

[42] Komaromy M, Duhigg D, Metcalf A, Carlson C, Kalishman S, Hayes L, et al. Project ECHO (Extension for Community Healthcare Outcomes): A new model for educating primary care providers about treatment of substance use disorders. Subst Abus. 2016;37(1):20–4. Epub 2016/02/06. doi: 10.1080/08897077.2015.1129388 . 26848803 PMC4873719

[43] Katon W, Von Korff M, Lin E, Walker E, Simon GE, Bush T, et al. Collaborative management to achieve treatment guidelines. Impact on depression in primary care. Journal of American Medical Association. 1995;273:1026–31. 7897786

[44] Huffman JC, Niazi SK, Rundell JR, Sharpe M, Katon WJ. Essential articles on collaborative care models for the treatment of psychiatric disorders in medical settings: a publication by the academy of psychosomatic medicine research and evidence-based practice committee. Psychosomatics. 2014;55(2):109–22. Epub 2013/12/29. doi: 10.1016/j.psym.2013.09.002 . 24370112

[45] Coventry PA, Hudson JL, Kontopantelis E, Archer J, Richards DA, Gilbody S, et al. Characteristics of effective collaborative care for treatment of depression: a systematic review and meta-regression of 74 randomised controlled trials. PLoS ONE. 2014;9(9):e108114. Epub 2014/09/30. doi: 10.1371/journal.pone.0108114 . 25264616 PMC4180075

[46] Gilbody S, Bower P, Fletcher J, Richards D, Sutton AJ. Collaborative care for depression: a cumulative meta-analysis and review of longer-term outcomes. Archives of internal medicine. 2006;166(21):2314–21. Epub 2006/11/30. doi: 10.1001/archinte.166.21.2314 . 17130383

[47] Thota AB, Sipe TA, Byard GJ, Zometa CS, Hahn RA, McKnight-Eily LR, et al. Collaborative care to improve the management of depressive disorders: a community guide systematic review and meta-analysis. Am J Prev Med. 2012;42(5):525–38. Epub 2012/04/21. doi: 10.1016/j.amepre.2012.01.019 . 22516495

[48] Melville JL, Reed SD, Russo J, Croicu CA, Ludman E, LaRocco-Cockburn A, et al. Improving care for depression in obstetrics and gynecology: a randomized controlled trial. Obstetrics and gynecology. 2014;123(6):1237–46. Epub 2014/05/09. doi: 10.1097/AOG.0000000000000231 . 24807320 PMC4052378

[49] Bhat A, Grote NK, Russo J, Lohr MJ, Jung H, Rouse CE, et al. Collaborative Care for Perinatal Depression Among Socioeconomically Disadvantaged Women: Adverse Neonatal Birth Events and Treatment Response. Psychiatr Serv. 2017;68(1):17–24. Epub 2016/10/04. doi: 10.1176/appi.ps.201600002 . 27691376

[50] Alford DP, LaBelle CT, Kretsch N, Bergeron A, Winter M, Botticelli M, et al. Collaborative care of opioid-addicted patients in primary care using buprenorphine: five-year experience. Archives of internal medicine. 2011;171(5):425–31. Epub 2011/03/16. doi: 10.1001/archinternmed.2010.541 . 21403039 PMC3059544

[51] Suzuki J, Matthews ML, Brick D, Nguyen M-T, Jamison RN, Ellner AL, et al. Implementation of a collaborative care management program with buprenorphine in primary care: A comparison between opioid-dependent patients and chronic pain patients using opioids non-medically. Journal of opioid management. 2014;10(3):159–68. doi: 10.5055/jom.2014.0204 24944066 PMC4085743

[52] Mittal L, Suzuki J. Feasibility of collaborative care treatment of opioid use disorders with buprenorphine during pregnancy. Subst Abus. 2017;38(3):261–4. Epub 2015/12/18. doi: 10.1080/08897077.2015.1129525 . 26672650

[53] Watkins KE, Ober AJ, Lamp K, et al. Collaborative care for opioid and alcohol use disorders in primary care: The summit randomized clinical trial. JAMA internal medicine. 2017;177(10):1480–8. doi: 10.1001/jamainternmed.2017.3947 28846769 PMC5710213

[54] Fals-Stewart W, O’Farrell TJ, Freitas TT, McFarlin SK, Rutigliano P. The timeline followback reports of psychoactive substance use by drug-abusing patients: psychometric properties. Journal of consulting and clinical psychology. 2000;68:134–44. doi: 10.1037//0022-006x.68.1.134 10710848

[55] Kriston L, Scholl I, Hölzel L, Simon D, Loh A, Härter M. The 9-item Shared Decision Making Questionnaire (SDM-Q-9). Development and psychometric properties in a primary care sample. Patient education and counseling. 2010;80(1):94–9. doi: 10.1016/j.pec.2009.09.034 19879711

[56] Maly RC, Frank JC, Marshall GN, DiMatteo MR, Reuben DB. Perceived efficacy in patient-physician interactions (PEPPI): validation of an instrument in older persons. Journal of the American Geriatrics Society. 1998;46(7):889–94. Epub 1998/07/22. doi: 10.1111/j.1532-5415.1998.tb02725.x . 9670878

[57] Kim SC, Boren D, Solem SL. The Kim Alliance Scale: development and preliminary testing. Clinical nursing research. 2001;10(3):314–31. Epub 2002/03/08. doi: 10.1177/c10n3r7 . 11881945

[58] Cella D, Yount S, Rothrock N, Gershon R, Cook K, Reeve B, et al. The Patient-Reported Outcomes Measurement Information System (PROMIS): Progress of an NIH Roadmap Cooperative Group During its First Two Years. Medical care. 2007;45(5 Suppl 1):S3–S11. doi: 10.1097/01.mlr.0000258615.42478.55 17443116 PMC2829758

[59] Cox JL, Holden JM, Sagovsky R. Detection of postnatal depression: Development of the 10-item Edinburgh Postnatal Depression Scale. The British Journal of Psychiatry. 1987;150:782–6. doi: 10.1192/bjp.150.6.782 3651732

[60] Cox JL, Chapman G, Murray D, Jones P. Validation of the Edinburgh Postnatal Depression Scale (EPDS) in non-postnatal women. J Affect Disord. 1996;Volume 39(Issue 3):185–9. 10.1016/0165-0327(96)00008-0 8856422

[61] Luoma JB, Twohig MP, Waltz T, Hayes SC, Roget N, Padilla M, et al. An investigation of stigma in individuals receiving treatment for substance abuse. Addict Behav. 2007;32(7):1331–46. Epub 2006/11/10. doi: 10.1016/j.addbeh.2006.09.008 . 17092656

[62] Williams ES, Konrad TR, Linzer M, McMurray J, Pathman DE, Gerrity M, et al. Refining the Measurement of Physician Job Satisfaction: Results from the Physician Worklife Survey. Medical Care. 1999;37(11):1140–54. doi: 10.1097/00005650-199911000-00006 10549616

[63] Chappel JN, Veach TL, Krug RS. The substance abuse attitude survey: an instrument for measuring attitudes. J Stud Alcohol. 1985;46(1):48–52. Epub 1985/01/01. doi: 10.15288/jsa.1985.46.48 . 3974235

[64] Khatri K, Haddad M, Anderson D. Project ECHO: replicating a novel model to enhance access to hepatitis C care in a community health center. Journal of health care for the poor and underserved. 2013;24(2):850–8. Epub 2013/06/04. doi: 10.1353/hpu.2013.0093 . 23728050

[65] Sobell LC, Brown J, Leo GI, Sobell MB. The reliability of the Alcohol Timeline Followback when administered by telephone and by computer. Drug Alcohol Depen. 1996;42(1):49–54. doi: 10.1016/0376-8716(96)01263-X 8889403

[66] Edgington E Randomization Tests. 2nd ed. New York, NY: Marcel Dekker; 1987.

[67] Gail MH, Mark SD, Carroll RJ, Green SB, Pee D. On design considerations and randomizatjion-based inference for community intervention trials. Statistics in medicine. 1996;15(11):1069–92. 8804140 10.1002/(SICI)1097-0258(19960615)15:11<1069::AID-SIM220>3.0.CO;2-Q

[68] Hayes RJ, Bennett S. Simple sample size calculation for cluster-randomized trials. International journal of epidemiology. 1999;28(2):319–26. doi: 10.1093/ije/28.2.319 10342698

[69] Guille C, Barth KS, Mateus J, McCauley JL, Brady KT. Treatment of Prescription Opioid Use Disorder in Pregnant Women. American Journal of Psychiatry. 2017;174(3):208–14. doi: 10.1176/appi.ajp.2016.16060710 28245688 PMC5446091

[70] MacAfee L, Sawyer A, Terplan M. Trends in receipt of medication-assisted treatment for pregnant women with opioid use disorder in the United States. Drug and Alcohol Dependence. 2017;171(Supplement C):e125. doi: 10.1016/j.drugalcdep.2016.08.347

[71] Martin CE, Longinaker N, Mark K, Chisolm MS, Terplan M. Recent trends in treatment admissions for marijuana use during pregnancy. J Addict Med. 2015;9(2):99–104. doi: 10.1097/ADM.0000000000000095 . 25525944

[72] Krans EE, Bobby S, England M, Gedekoh RH, Chang JC, Maguire B, et al. The Pregnancy Recovery Center: A women-centered treatment program for pregnant and postpartum women with opioid use disorder. Addict Behav. 2018;86:124–9. Epub 2018/06/10. doi: 10.1016/j.addbeh.2018.05.016 . 29884421

[73] Saia K, Bagley SM, Wachman EM, Patel PP, Nadas MD, Brogly SB. Prenatal treatment for opioid dependency: observations from a large inner-city clinic. Addiction science & clinical practice. 2017;12(1):5. Epub 2017/01/15. doi: 10.1186/s13722-016-0070-9 . 28086944 PMC5237261

[74] Brogly SB, Saia KE, Werler MM, Regan E, Hernandez-Diaz S. Prenatal Treatment and Outcomes of Women With Opioid Use Disorder. Obstetrics and gynecology. 2018. Epub 2018/09/12. doi: 10.1097/AOG.0000000000002881 . 30204704 PMC6153027

