# Supplementary material for: Support Models for Addiction Related Treatment (SMART) for pregnant women: Study protocol of a cluster randomized trial of two treatment models for opioid use disorder in prenatal clinics
Source: PLoS One. 2022 Jan 13;17(1):e0261751. doi: 10.1371/journal.pone.0261751 (PMC8758001; doi:10.1371/journal.pone.0261751)
Supplement: S1 File — (PDF) [file pone.0261751.s002.pdf]

COMPOUND AUTHORIZATION AND CONSENT FOR PARTICIPATION IN A RESEARCH STUDY

YALE UNIVERSITY SCHOOL OF MEDICINE AND YALE NEW HAVEN HOSPITAL & YALE MEDICINE & BRIDGEPORT HOSPITAL

Study Title: Support Models for Addiction Related Treatment (SMART) Trial of Opioid Use Disorder in Pregnant Women

Principal Investigator (the person who is responsible for this research:
Ariadna Forray, MD
40 Temple street Suite 6B
New Haven CT 06510
United States

YNHH Site PIs: Heather Lipkind, MD & Shefali Pathy, MD

Yale Medicine Site PI: Amy Snyder, MD

Bridgeport Hospital Site PIs: Crina Boeras, MD & Jean Tornatore, MD

Research Study Summary:

- We are asking you to join a research study.
- The purpose of this research study is to try to improve treatment for pregnant and post-partum women who have opioid use disorder.
- Study activities will include: If you agree to participate in this study, your in-person research visits will take place at your obstetric provider's office before or after your regularly scheduled prenatal visits. During some of these visits (at the beginning of the study, at 26 weeks of pregnancy, at 36 weeks of pregnancy and 3 months postpartum) we will ask you to complete questionnaires on a computer tablet. We will also ask that you provide a urine sample that will be tested for substances at the 26 and 36 week visits. A member from the research team will call you monthly and ask about any treatment you may have received and any substances you may have used (eg. opioids, alcohol, tobacco, etc)
- Your involvement will require approximately 45–60 minutes for screening and baseline assessment, 20-30 minutes during up to 3 assessment points and about 5-10 minutes per month by phone. Your first monthly phone call may take longer, about 15 or 20 minutes.
- We believe there are very few risks from participating in this study. We would like you to tell us about your use of opioids and other substances during the study. We know that stopping use of opioids can be quite difficult. There can also be stigma associated with admitting use of substance during pregnancy. Stigma is when a person disapproves of or discriminates against a person. Stigma can be unfair and biased. In order to be helpful to you we need to know about your substance use. We only ask that you do your best to stop using drugs, be honest about yourself and your problems, and be available to complete an assessment during your appointment times and during the monthly phone call.
- We cannot promise any benefits to you or others from your taking part in this research. However, possible benefits to you include that it may help you to better manage your opioid use disorder. Your participation may also help women in the future if one of these systems

APPROVED BY THE YALE UNIVERSITY IRB 12/9/2020

of care is more helpful than the other to obstetric teams that take care of pregnant women who use opioids.

- There are other choices available to you outside of this research. Instead of being part of this research, you can continue your regular prenatal care with your obstetrician and other providers and not complete the questionnaires that are part of this study.
- Taking part in this study is your choice. You can choose to take part, or you can choose not to take part in this study. You also can change your mind at any time. Whatever choice you make will not have any effect on your relationship with your obstetrical provider
- If you are interested in learning more about the study, please continue reading, or have someone read to you, the rest of this document. Ask the study staff questions about anything you do not understand. Once you understand the study, we will ask you if you wish to participate; if so, you will have to sign this form.

Why is this study being offered to me?
We are asking you to take part in a research study because you screened positive for use of an opioid medication or drug during pregnancy. People who use illegal opioids or misuse prescription opioid pain pills may have opioid use disorder. Pregnant women who have opioid use disorder may require treatment for this problem at the same time they receive prenatal care. The purpose of this research is to try to improve treatment for pregnant and postpartum women who have an opioid use disorder. We are looking for 480 women, across 12 obstetrical practices, to be part of this research study.

Who is paying for the study?
This study is being funded by the Patient Centered Outcome Research Institute (PCORI). PCORI is a US-based not for profit institute created through the 2010 Patient Protection and Affordable Care Act. It is a government sponsored organization that aims to “fund research that can help patients and those who care for them make better-informed decisions about healthcare choices.”

What is the study about?
The purpose of this study is to compare two models of education, clinical training and support for clinicians who provide obstetrical care for pregnant women with opioid use disorder.

What are you asking me to do and how long will it take?
If you agree to take part, your participation in this study will involve completing assessments on a computer tablet. We ask that you complete them at your obstetric provider’s office before or after your regularly scheduled prenatal visits at the beginning of the study, at 26 weeks of pregnancy, at 36 weeks of pregnancy. If you prefer we can send you these assessments as email surveys. The 3 months postpartum assessments will be sent to you as an email survey. If you are further along than 26 weeks we ask that you only complete the 36 week and 3 month postpartum assessments. The assessments ask questions about your relationship with your obstetrician; whether you feel your obstetrician listens to you; questions about your health; questions about health habits such as use of substances; questions about any history of traumatic events in your life. We will also ask that you provide a urine sample that will be tested for substances. We think that the study assessments will take 20 minutes of your time. We will also call you monthly and ask about any treatment you have received and use of substances. The first monthly phone call will take about 15 to 20 minutes of your time. After that, they will take 5-10 minutes of your time.

Are there any risks from participating in this research?

We do not expect many risks from taking part in this study but there are some. While we take steps to protect any confidential health information, there is always a risk of loss of confidentiality. Also, there may be stigma associated with admitting use of substances in pregnancy; stigma is when a person disapproves of or discriminates against a person based on some characteristic they notice. Stigma can be unfair and biased. We have tried to educate everyone involved in this project about stigma but you may still feel uncomfortable talking about these issues.

How can the study possibly benefit me or others?

You may benefit from participating in the study since it may help you to better manage your opioid use disorder, although we cannot guarantee this. We hope that our results will add to the knowledge about the best treatments of opioid use disorder in pregnancy by showing whether one model of support for obstetricians is better than the other model.

Are there any costs to participation?

You will not have to pay for taking part in this study. The only costs may include transportation and your time coming to the study visits. Most of these visits will be at your regularly scheduled prenatal care visits.

Will I be paid for participation?

You will be paid up to $185 for taking part in this study:

- For comprehensive eligibility screening you will receive an Amazon gift card for $5
- If you continue to be eligible and complete the Intake Assessment you will receive a $25 Amazon gift card; For Week 26 of pregnancy visit you will receive a $25 Amazon gift card; for Week 36 of pregnancy you will receive a $25 Amazon gift card; for 3 month post-partum visit you will receive a $50 Amazon gift card; for the first monthly phone assessment you will receive a $15 Amazon gift card. For each monthly phone assessment you complete after the first one you will receive a $5 Amazon gift card

  Additionally, you may be invited to do two additional interviews over the phone. This interview would involve you giving your opinions on the opioid use treatment you are receiving. If you are interested in participating in these optional interviews, we will have you read a separate consent later in the study. You would be paid an additional $50 for each interview for a total of $100.

How will you keep my data safe and private?

All of your responses will be held in close confidence. All of the information you provide will be listed under a study subject identification number rather than your name or any other identifying information. We will remove identifiers from identifiable private information. The list linking your name and code will be kept in REDCap secure data system, which is protected by password and encryption software. A hard copy will be kept in a locked filing cabinet at Yale University. Only study team members will have access to those codes. Only the researchers involved in this study and those responsible for research oversight (such as rep
resentatives of the Yale University Human Research Protection Program, the Yale University Institutional Review Boards, and others) will have access to any information that could identify you that you provide. We will share it with others if you agree to it or when we have to do it because U.S. or State law requires it. For example, we will tell somebody if we learn that you are putting a child or older person in immediate danger (see below).

APPROVED BY THE YALE UNIVERSITY IRB 12/9/2020

If you decide to take part in this research study, you will be required to give us information about your substance use. We have obtained a Certificate of Confidentiality (CoC) issued by the National Institute of Health (NIH). Researchers can use this Certificate to legally refuse to disclose information that may identify you in any federal, state, or local civil, criminal, administrative, legislative, or other proceedings, for example, if there is a court subpoena. The researchers will use the Certificate to resist any demands for information that would identify you, except as explained below.

The protection offered by the CoC does not stop us from voluntarily reporting information about suspected or known sexual, physical, or other abuse of a child or older person, or a participant's threats of violence to self or others. If any member of the research team is given such information, he or she will make a report to the appropriate authorities. Even when a CoC is in place, you and your family members must still continue to actively protect your own privacy. If you voluntarily give your written consent for anyone to receive information about your participation in the research, then we may not use the CoC to withhold this information.

When we publish the results of the research or talk about it in conferences, we will not use your name. If we want to use your name, we would ask you for your permission.

We will also share information about you with other researchers for future research but we will not use your name or other identifiers. We will not ask you for any additional permission.

What Information Will You Collect About Me in this Study?

The information we are asking to use and share is called "Protected Health Information." It is protected by a federal law called the Privacy Rule of the Health Insurance Portability and Accountability Act (HIPAA). In general, we cannot use or share your health information for research without your permission. If you want, we can give you more information about the Privacy Rule. Also, if you have any questions about the Privacy Rule and your rights, you can speak to Yale Privacy Officer at 203-432-5919.

The specific information about you and your health that we will collect, use, and share includes:

- Research study records (assessments)
- Medical and laboratory records of only those services provided in connection with this Study.
- Information on your babies' birth and any medical complications you or your baby had at delivery
- Results of urine drug tests
- Records about phone calls made as part of this research
- Records about your study visits
- Information obtained during this research regarding
    - HIV / AIDS test results
    - Hepatitis infection
    - Sexually transmitted diseases
    - Other reportable infectious diseases
    - Physical exams
    - Laboratory, x-ray, and other test results
    - The diagnosis and treatment of a mental health condition
    - Use of illegal drugs or the study of illegal behavior

APPROVED BY THE YALE UNIVERSITY IRB 12/9/2020

How will you use and share my information?
We will use your information to conduct the study described in this consent form.
We may share your information with:

- Representatives from Yale University, the Yale Human Research Protection Program and the Institutional Review Board (the committee that reviews, approves, and monitors research on human participants), who are responsible for ensuring research compliance. These individuals are required to keep all information confidential.
- Governmental agencies to whom certain diseases (reportable diseases) must be reported
- Health care providers who provide services to you in connection with this study.
- Co-Investigators and other investigators
- Study Coordinator and Members of the Research Team
- Data and Safety Monitoring Boards and others authorized to monitor the conduct of the Study

We will do our best to make sure your information stays private. But, if we share information with people who do not have to follow the Privacy Rule, your information will no longer be protected by the Privacy Rule. Let us know if you have questions about this. However, to better protect your health information, agreements are in place with these individuals and/or companies that require that they keep your information confidential.

Why must I sign this document?
By signing this form, you will allow researchers to use and disclose your information described above for this research study. This is to ensure that the information related to this research is available to all parties who may need it for research purposes. You always have the right to review and copy your health information in your medical record.

What if I change my mind?
The authorization to use and disclose your health information collected during your participation in this study will never expire. However, you may withdraw or take away your permission at any time. You may withdraw your permission by telling the study staff or by writing to Dr. Ariadna Forray, 40 Temple Street, Suite 6B, New Haven, CT 06510.

If you withdraw your permission, you will not be able to stay in this study but the care you receive from your doctor outside this study will not change. No new health information identifying you will be gathered after the date you withdraw. Information that has already been collected may still be used and given to others until the end of the research study to insure the integrity of the study and/or study oversight.

What if I want to refuse or end participation before the study is over?
Taking part in this study is your choice. You can choose to take part, or you can choose not to take part in this study. You also can change your mind at any time. Whatever choice you make will not have any effect on your relationship with your obstetrician or nurse at Yale School of Medicine or Yale New Haven Hospital

APPROVED BY THE YALE UNIVERSITY IRB 12/9/2020

Who should I contact if I have questions?
Please feel free to ask about anything you don't understand.

If you have questions later or if you have a research-related problem, you can call the Principal Investigator at (203) 764-8620

If you have questions about your rights as a research participant, or you have complaints about this research, you call the Yale Institutional Review Boards at (203) 785-4688 or email hrpp@yale.edu.

A description of this clinical trial will be available on http://www.ClinicalTrials.gov, as required by U.S. Law. This Web site will not include information that can identify you. At most, the Web site will include a summary of the results. You can search this Web site at any time.

Please answer the following questions before signing

I may withdraw from this study at any time	☐ Yes	☐ No
I will answer questions on a computer tablet at my obstetrician visits	☐ Yes	☐ No
I am asked for permission to review my medical record	☐ Yes	☐ No

Authorization and Documentation of Consent

Your signature below indicates that you read and understand this consent form and the information presented and that you agree to be in this study.

We will give you a copy of this form.

Participant Printed Name	Participant Signature	Date
Person Obtaining Consent Printed Name	Person Obtaining Consent Signature	Date

APPROVED BY THE YALE UNIVERSITY IRB 12/9/2020
