# Supplementary material for: Support Models for Addiction Related Treatment (SMART) for pregnant women: Study protocol of a cluster randomized trial of two treatment models for opioid use disorder in prenatal clinics
Source: PLoS One. 2022 Jan 13;17(1):e0261751. doi: 10.1371/journal.pone.0261751 (PMC8758001; doi:10.1371/journal.pone.0261751)
Supplement: S2 File — (PDF) [file pone.0261751.s003.pdf]

APPROVED BY THE YALE UNIVERSITY IRB 12/9/2020

Title: Support Models for Addiction Related Treatment (SMART) Trial of Opioid Use Disorder in Pregnant Women

Protocol Number: 2000027031

National Clinical Trial (NCT) Identified Number: NCT04240392

Principal Investigator: Ariadna Forray, MD

Funding: Patient Centered Outcomes Research Institute (PCORI)

Protocol Version: 5.0

Version Date: December 3, 2020

APPROVED BY THE YALE UNIVERSITY IRB 12/9/2020

APPROVED BY THE YALE UNIVERSITY IRB 12/9/2020

Table of Contents

STATEMENT OF COMPLIANCE 1
INVESTIGATOR'S SIGNATURE 2
1 PROTOCOL SUMMARY 3
1.1 Synopsis 3
1.2 Schema 5
1.3 Schedule of Activities 6
2 INTRODUCTION 7
2.1 Study Rationale 7
2.2 Background 8
2.3 Risk/Benefit Assessment 11
2.3.1 Known Potential Risks 11
2.3.2 Known Potential Benefits 11
2.3.3 Assessment of Potential Risks and Benefits 12
3 OBJECTIVES AND ENDPOINTS 13
4 STUDY DESIGN 16
4.1 Overall Design 16
4.2 Scientific Rationale for Study Design 17
4.3 Justification for Intervention 18
4.4 End-of-Study Definition 19
5 STUDY POPULATION 20
5.1 Inclusion Criteria 20
5.2 Exclusion Criteria 20
5.3 Lifestyle Considerations 21
5.4 Screen Failures 21
5.5 Strategies for Recruitment and Retention 21
6 STUDY INTERVENTION(S) OR EXPERIMENTAL MANIPULATION(S) 22
6.1 Study Intervention(s) or Experimental Manipulation(s) Administration 22
6.1.1 Study Intervention or Experimental Manipulation Description 22
6.1.2 Administration and/or Dosing 23
6.2 Fidelity 23
6.2.1 Interventionist Training and Tracking 23
6.3 Measures to Minimize Bias: Randomization and Blinding 24
6.4 Study Intervention/Experimental Manipulation Adherence 24
6.5 Concomitant Therapy 25
6.5.1 Rescue Therapy 25
7 STUDY INTERVENTION/EXPERIMENTAL MANIPULATION DISCONTINUATION AND PARTICIPANT DISCONTINUATION/WITHDRAWAL 26
7.1 Participant Discontinuation/Withdrawal from the Study 26
7.2 Lost to Follow-Up 26
8 STUDY ASSESSMENTS AND PROCEDURES 27
8.1 Endpoint and Other Non-Safety Assessments 27
8.1.1 Screening Procedures 27
8.1.2 Standard of Care Prenatal Visit 28
8.1.3 Timeline Follow Back Visits 28
8.1.4 Week 26 Visit 28
8.1.5 Week 34 Visit 29
8.1.6 Birth 29
8.1.7 3 Months Post-Partum Visit 29
8.2 Safety Assessments 30

APPROVED BY THE YALE UNIVERSITY IRB 12/9/2020

APPROVED BY THE YALE UNIVERSITY IRB 12/9/2020

8.3 Adverse Events and Serious Adverse Events 30
8.3.1 Definition of Adverse Events 30
8.3.2 Definition of Serious Adverse Events....30
8.3.3 Classification of an Adverse Event 30
8.3.4 Time Period and Frequency for Event Assessment and Follow-Up 31
8.3.5 Adverse Event Reporting 32
8.3.6 Serious Adverse Event Reporting 32
8.3.7 Events of Special Interest 32
8.4 Unanticipated Problems....32
8.4.1 Definition of Unanticipated Problems 32
8.4.2 Unanticipated Problems Reporting 33
9 STATISTICAL CONSIDERATIONS....33
9.1 Statistical Hypotheses 33
9.2 Sample Size Determination 34
9.3 Populations for Analyses 34
9.4 Statistical Analyses 35
9.4.1 General Approach....35
9.4.2 Analysis of the Primary Endpoint(s)....35
9.4.3 Analysis of the Secondary Endpoint(s) 35
9.4.4 Baseline Descriptive Statistics 36
9.4.5 Planned Interim Analyses 36
9.4.6 Sub-Group Analyses....36
9.4.7 Tabulation of Individual Participant Data 36
9.4.8 Exploratory Analyses 36
10 SUPPORTING DOCUMENTATION AND OPERATIONAL CONSIDERATIONS 37
10.1 Regulatory, Ethical, and Study Oversight Considerations 37
10.1.1 Informed Consent Process....37
10.1.2 Study Discontinuation and Closure....37
10.1.3 Confidentiality and Privacy 37
10.1.4 Safety Oversight....38
10.1.5 Clinical Monitoring 38
10.1.6 Quality Assurance and Quality Control....38
10.1.7 Data Handling and Record Keeping 39
10.1.8 Protocol Deviations 40
11 REFERENCES....41
12 APPENDIX: Study Personnel and Roles....50

APPROVED BY THE YALE UNIVERSITY IRB 12/9/2020

STATEMENT OF COMPLIANCE

This trial will be conducted in compliance with the protocol, International Council on Harmonization Good Clinical Practice (ICH GCP) and applicable state, local and federal regulatory requirements. Each engaged institution must have a current *Federal-Wide Assurance (FWA)* issued by the Office for Human Research Protections (OHRP) and must provide this protocol and the associated informed consent documents and recruitment materials for review and approval by an appropriate Institutional Review Board (IRB) or Ethics Committee (EC) registered with OHRP. Any amendments to the protocol or consent materials must also be approved before implementation.

The protocol, informed consent form(s), recruitment materials, and all participant materials will be submitted to the IRB for review and approval. Approval of both the protocol and the consent form(s) must be obtained before any participant is consented. Any amendment to the protocol will require review and approval by the IRB before the changes are implemented to the study. All changes to the consent forms will be IRB approved; a determination will be made regarding whether a new consent needs to be obtained from participants who provided consent, using a previously approved consent form by the IRB of record.

INVESTIGATOR'S SIGNATURE

The signature below constitutes the approval of this protocol and provides the necessary assurances that this study will be conducted according to all stipulations of the protocol, including all statements regarding confidentiality, and according to local legal and regulatory requirements and applicable US federal regulations and ICH guidelines, as described in the *Statement of Compliance* above.

Principal Investigator:

Signed: ______________________________ Date: ______________

Name*:

Title*:

Investigator Contact Information

Affiliation*:
Address:
Telephone:
Email:

1 PROTOCOL SUMMARY

1.1 SYNOPSIS

Title: Support Models for Addiction Related Treatment (SMART) Trial of Opioid Use Disorder in Pregnant Women

Grant Number: MAT-2018C2-12891

Study Description: Between 1999 and 2014 the point prevalence of an Opioid Use Disorder (OUD) in pregnant women increased from 1.5 to 6.5 per 1000 deliveries, which is in line with rates of opioid prescribing in the general population.(1) Unfortunately, providers of outpatient medication assisted treatment (MAT) are less likely to treat pregnant women compared to non-pregnant women (75% vs 91%).(2) The provision of MAT in reproductive health settings may augment the capacity to treat pregnant women with an OUD and increase treatment rates, but the optimal models for care need to be determined. We propose a cluster randomized clinical trial to compare the effectiveness of two models of support for reproductive health clinicians to provide care for pregnant and postpartum women with an OUD: 1) a collaborative care (CC) approach based upon the Massachusetts Office-Based-Opioid Treatment (OBOT) Model,(3) that would provide onsite training, and support to providers and patients through the use of a care managers (CMs) vs 2) a telesupport approach modeled on the Project Extension for Community Healthcare Outcomes (ECHO), a remote education model that provides mentorship and guided practice and participation in a learning community, via video conferencing.(4)

**Objectives:**	1) To determine differences in engagement and retention between participants who receive care from a center that uses a CC vs an ECHO support model. We hypothesize that the benefit from a CM and proactive monitoring approach will lead to greater treatment engagement and retention among patient participants.<br><br>H_1: Participants with an OUD are more likely to be *engaged* in care if the center is randomized to the CC vs the ECHO model of support.<br>H_2: Participants with an OUD treated in CC centers are more likely to be *retained* in MAT and/or non-pharmacological care for an OUD than those treated in a center randomized to the ECHO model of care.<br>2) To determine differences in patient-reported outcomes between the two treatment models. For this domain we will use the **Patient Activation Measure (PAM)** as the sentinel measure, operationalized by assigning patients to one of four PAM levels based upon their scores.<br>H_3: Participants in the CC, compared to the ECHO condition, are more likely to increase at least one PAM level from baseline to delivery and 3-months post-partum.
**Endpoints:**	Treatment Retention<br>Treatment Engagement<br>Patient Activation Measure (PAM)<br>Birth Outcomes
**Study Population:**	480 pregnant women with opioid use disorder
**Phase or Stage:**	Phase 3
**Description of Sites/Facilities:**	12 characteristically matched obstetrical centers in Connecticut and Massachusetts.
**Study Intervention/Experimental Manipulation:**	Collaborative Opioid Prescribing Model (CC)<br>Project ECHO (Extension for Community Healthcare Outcomes)
**Study Duration:**	4 years
**Participant Duration:**	Approximately 44 weeks (8 weeks pregnant– 3 months post-partum)

APPROVED BY THE YALE UNIVERSITY IRB 12/9/2020

1.2 SCHEMA

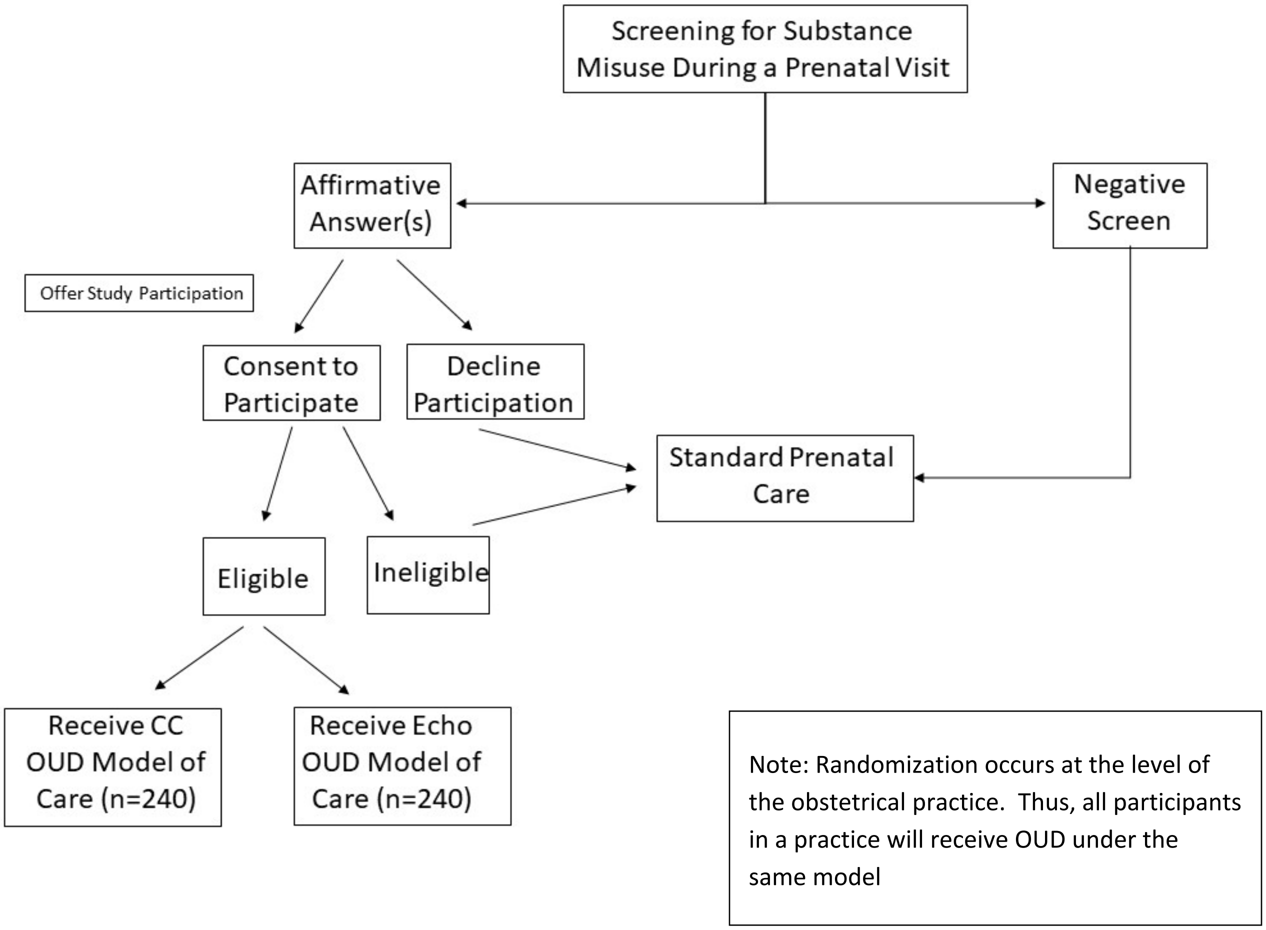

APPROVED BY THE YALE UNIVERSITY IRB 12/9/2020

APPROVED BY THE YALE UNIVERSITY IRB 12/9/2020

1.3 SCHEDULE OF ACTIVITIES

	Study Screening	Visit 1[3] (BLine)	Visit 2[3]	Visit 3[3]	Visit 4[3]	Visit 5[3] 26 Week Visit	Visit 6[3]	Visit 7[3]	Visit 8[3] 36 Week Visit	Visit 9[3]	Visit 10[3] Birth	Visit 11[3] 3 Months post-partum visit
**Window**						+/- 4 weeks			+/- 4weeks			+/- 4 weeks
Informed Consent	X											
NIDA Quickscreen	X											
Demographics	X											
Concomitant Medication		X	X	X	X	X	X	X	X	X		
Medical History		X								X		
Pregnancy Questionnaire	X											
Urine Drug Screening[1]		X---X										
DSM-5 OUD Checklist[6]	X											
Contact Information		X										
Assigned OUD Treatment[2]		X---X										
Time Line Follow Back[4]		X	X	X	X	X	X	X	x	X		
Post Traumatic Stress Disorrder Checklist for DSM-5 (PCL-5)		X										
Fagerstrom Test for Nicotine Dependence (FTND)		X				X			X			X
HIV Risk -Taking Behavior Scale (HRBS)		X										
Patient Activation Measure (PAM)		X				X			X			X
Shared-Decision Making Questionnaire-9 (SDM-Q-9)		X				X			X			X
Perceived efficacy in Patient-Physician Interactions Questionnaire (PEPPI)		X				X			X			X
Kim Alliance Scale-Communication subscale (KAS-CM)		X				X			X			X
PROMIS Emotional Short Form 4a		X				X			X			X
PROMIS Satisfactions with Roles and Activities Short form 8a		X				X			X			X
Satisfaction with Roles and Activities		X				X			X			X
Edinburgh Postnatal Depression Scale (EPDS)		X				X			X			X
Adverse Childhood Experience Questionnaire		X										
Stigma Related Rejection Scale						X						X
Treatment Utilization Form		X	X	X	X	X	X	X	X	X		
Substance Abuse Attitude Survey (Treatment Optimism)[8]												

APPROVED BY THE YALE UNIVERSITY IRB 12/9/2020

Physicians Worklife Survey(PWS)[8]												
Birth Outcomes)[7]											X	
Qualitative Interviews (sub group						X						X

1. Standard of care urine drug screens will be tested for the presents of illicit and licit opiate drugs at every prenatal visit after consent. Results are recorded by the clinic staff and faxed to the Yale research assistant.
2. Participants will receive OUD treatment via the clinic assigned treatment method (either CC or ECHO) throughout the length of their participation.
3. Participant will enter the study at different point of pregnancy. Therefore, it is likely that participants will complete varying numbers of visits between screening and the 26-week visit, and the 34-week visit and birth. This is expected and is not considered a protocol deviation. Visit 1 will follow immediately after the consenting process
4. The Time Line Follow Back will be conducted monthly during the length of the study. The number of TLFB assessments will vary depending on when the patient enters the study and when they give birth.
5. All participating centers will perform a drugs screen such as the NIDA Quick Screen or equivalent validated instrument as standard of care. If it is positive the DSM-5 OUD checklist will be completed to determine study eligibility.
6. The DSM-5 OUD Checklist will be administered to any patients who have a positive NIDA Quick Screen as part of the standard of care.
7. Birth outcomes include: low birth weight, resuscitation at delivery, fetal demise, preterm birth, duration of hospitalization.
8. The Substance Abuse Attitude Scale, the Physicians Worklife Survey and the Qualitative Interviews will be completed by physicians And done prior to the first participant being enrolled at the site and after the last participant is enrolled at the site.

2 INTRODUCTION

2.1 STUDY RATIONALE

In 2015, an estimated 3.8 million individuals in the US misused pain relievers and over 1.9 million suffered from an OUD.(5, 6) The lethality of opioid misuse is staggering in that more than 90 people in the US die each day from an opioid overdose for a total of 33,000 deaths in 2015.(7) Pregnant women have not been spared. About 5% of pregnant women report nonmedical use of opioids in the prior year and 1% report nonmedical use in the preceding month.(8) Similarly, an analysis of commercial insurance beneficiaries finds that 2.2% of pregnant women were dispensed opioids at least 3 times in pregnancy.(9) Not only is nonmedical use of opioids a problem in and of itself, it primes individuals to transition to illicit opioids such as heroin. National data from the Centers for Disease Control found a startling upsurge in the rates of an OUD among obstetrical inpatients, increasing 333% from 1.5 to 6.5 cases per 1000 deliveries between 1999-2014. (1) Although data from **Connecticut (CT)** were not available for this analysis, the rate of change in **Massachusetts (MA)** was 6th out of the 30 states and the District of Columbia that contributed data.

The potential consequences of maternal opioid misuse in pregnancy are documented extensively and include maternal and fetal effects. **Neonatal opioid withdrawal syndrome (NOWS)** has received widespread attention (10) as rates of maternal opioid use have risen. National estimates for NOWS show escalations from 3.4 to 5.8 per 1000 hospital births between 2009 and 2012. (11) In utero opioid exposure also increases the risk of premature rupture of membranes,(12) preterm birth,(12) small for gestational age delivery,(13) and still birth.(12) On the maternal side, opioid use in pregnancy is associated with placental abruption, preterm labor, cardiac arrest and death.(12) Indeed, a recent analysis finds that the 300 percent increase in opioid use among pregnant women during the study period paralleled an upsurge in cardiac events, the leading cause of death in pregnancy.(14) The annual

percent increment in risk of an acute heart attack or cardiac arrest between 2002 and 2014 was 13% for women who used opioids during pregnancy. (14) These data illustrate the need for treatment of an OUD in pregnancy.

The context for women who misuse opioids in pregnancy needs to be considered. For example, poverty, unstable housing, interpersonal violence and food insecurity occur frequently among women with an OUD; these factors can influence maternal, fetal and neonatal health.(15-18) General medical conditions can complicate pregnancy. About 60-70% of pregnant women who inject drugs and use opioids in pregnancy are positive for Hepatitis C (19, 20) and ~1% are HIV positive.(21) As well, co-use of substances that have deleterious effects on maternal and fetal outcomes,(22-26) particularly benzodiazepines, cocaine, nicotine and alcohol, are a problem.(27-29) A third component to morbidity derives from concurrent psychiatric illness confronting pregnant women with substance use disorders. Among women with non-medical prescription opioid use, more than 40% have a depressive or anxiety disorder.(30)

2.2 BACKGROUND

Treatment for pregnant women with an OUD includes medication and behavioral therapy/psychosocial support.(31-34) At minimum, psychosocial support should include an assessment of the mother's needs and referral to psychosocial treatment services.(32) **Medication assisted treatment (MAT)** is a critical component of standard care for pregnant women with an OUD (35, 36) and is recommended by the **American College of Obstetricians and Gynecologists (ACOG)**(33) and other experts.(5, 37) It enhances adherence to prenatal care (38) and increases the likelihood that the mother will continue MAT after delivery, a period of high risk among perinatal women with OUD.(39) Initially MAT included methadone but now includes buprenorphine and naltrexone (not yet indicated for use in pregnancy), which can be prescribed in primary care settings.(40)

Some women still wish to forgo MAT in pregnancy and prefer medically-assisted withdrawal. Concerns are the risk of NOWS and other neonatal complications,(41, 42) but equally important to consider are the high rates of relapse to substance misuse (17%-96%; average 48%),(42) and other complications among women who decline MAT.(38, 43-45) Women must be fully informed of the risks and benefits of MAT in order to make a decision. As well, women must be educated and engaged in making decisions regarding the type of MAT, if any, to accept. For example, birth outcomes may be slightly improved under buprenorphine versus methadone treatment (36) although differences are small and reflect the characterstics of patients engaged in one or the other treatment.(46)

The treatment of pregnant women with OUD entails greater complexity than typically found for non-pregnant individuals with OUD. Pregnant women require regular prenatal care and for those receiving MAT, dosage adjustments.(5, 36, 40, 47) We note the high rates of infections above. Unfortunately, access to care for pregnant women with an OUD can be challenging.(2, 17) One model,(48) applied to pregnant women with OUD,(49) describes difficulties with treatment access in the following domains: **availability** (wait lists, absence of specialized addiction programs, centers that will not enroll pregnant women);(2, 15) **accessibility** (limited transportation, competing time demands given child care, lack of family and friend support);(50) **affordability** (lack of insurance or other financial);(51) and **acceptability** (concerns over reports to child protective services,(52) stigma/shame related to the illness, provider attitudes toward illness and MAT).(50, 53) While addiction treatment admissions among pregnant women with OUD have increased (26, 54, 55) the use of MAT remains low, with one group finding a rate of 37% in 2010(26) and 46% in 2012.(55) The legitimate concerns many women have about the possible

APPROVED BY THE YALE UNIVERSITY IRB 12/9/2020

effects of gestational medication use on their offspring may deter use of MAT. However, under-treatment is not limited to medication use since completion rates in specialty treatment programs during this critical reproductive time were only 66%, likely reflecting the barriers noted above.(26)

Given the scope of opioid crisis, the need for treatment outpaces the capacity to provide it.(56) Increasing the cadre of buprenorphine providers can help address this but there are barriers including: lack of patient and physician interest,(57-59) lack of expertise and education,(57, 58, 60, 61) limited physician time and resources, (57, 62, 63) stigma towards patients with OUD,(58, 61) concern about abuse or diversion,(56, 64) lack of institutional support,(56, 59, 62) cumbersome regulations,(60, 65) and insurance barriers (e.g. insufficient rates of reimbursement).(56, 57, 61, 63) Provider barriers may be particularly problematic for pregnant women with an OUD.(66) A possible solution is to combine addiction care (including MAT) with prenatal care.(50, 67-69) Some existing programs provide "women-centered" care to patients with an OUD, including prenatal care, MAT, care coordination, behavioral health services, family planning and breastfeeding support.(18, 67, 70) Such programs are more likely than non-women-centered programs, to dose opiate agonist treatment (buprenorphine) to fit the physiology of pregnancy, enhance the utilization of long-acting contraception and have better rates of breastfeeding continuation.(67) Despite the potential benefits of a program that provides conjoint prenatal and addiction care, **there is a major gap in knowledge regarding the best models for supporting centers that would like to provide such women-centered care to women with an OUD treated in obstetrical settings.** The models of care we propose to test address many of these concerns, although they differ in their potential to affect many of them: 1) we know that the cadre of physicians, including obstetricians, who are trained to provide MAT is insufficient; 2) we also know that among those who have been waivered to use MAT, many obstetrical providers do not provide MAT; 3) indeed, many obstetricians identify lack of support for their provision of addiction care, especially given the complex needs of this patient population (71) as the reason they do not provide MAT. There is a need to compare different types of support to obstetrical practices that would allow them to deliver addiction care, in a women-centered manner.

<u>Comparative Results on Maternal and Fetal Outcomes and Patients' Perception of Care</u> **<u>(RQ-3)</u>**: If addiction treatment, including MAT, is provided through obstetrical practices, we need to do it with fidelity and in a way that meets patients' needs. Only a few studies have assessed possible differences in maternal and fetal well-being for gravidas treated in programs that provided combined vs separate prenatal care and addiction treatment.(67) Some cohort studies provided information on fetal outcomes in centers that provide combined care.(18, 20) We were unable to identify studies or trials that compared different models of combined prenatal and substance use care for pregnant women with OUD. Gaps in this literature are many, some of which include: rates of engagement in addiction treatment; level of retention in prenatal care and addiction treatment; rates of adverse birth outcomes; utilization of MAT as part of addiction treatment; rates of continuing MAT during the postpartum period; treatment of concurrent substance misuse and psychiatric conditions.

As well, but equally important is the lack of knowledge regarding women's experiences when they receive treatment under different models of combined care. Some of the domains of importance include patient's engagement in care, knowledge of care, confidence in managing pregnancy and their addiction, alliance with their providers, emotional well-being, ability to fulfill important roles in life, and retain custody of their children if they wish to do so. A trial of different models of care can provide information to fill in these important gaps.

APPROVED BY THE YALE UNIVERSITY IRB 12/9/2020

Primary Care Models of Addiction Treatment **(RQ-5)**: A technical report by the Agency for Healthcare Policy and Research **(AHRQ)** (72) outlines 12 models of care to provide MAT for OUD. Although they include "Integrated Prenatal Care and Medication-Assisted Treatment" as a model, they acknowledge that it is an insufficiently tested model and few details of such a model are articulated. We focus on two of the other 11 models that could be used to integrate prenatal care and addiction treatment: 1) Collaborative Opioid Prescribing Model (CC) and 2) Project Extension for Community Healthcare Outcomes (ECHO).

Collaborative Opioid Prescribing Model (Massachusetts Model): This model is based upon CC, a system developed initially to enhance the capacity for treatment of depression in primary care settings.(73) The model has since been refined (74, 75) and tested for treatment of other disorders; it has been deployed in additional venues including obstetrical-gynecological settings.(76, 77) CC, as articulated by the University of Washington, includes several components: (1) patients are systematically evaluated (screened) for medical and behavioral health needs; (2) a care manager assists with assessment and enrolls patients in a registry that includes data on symptoms that are collected longitudinally; the care manager provides some proactive behavioral treatment and care coordination; 3) a psychiatrist reviews cases and assists the primary physician in providing stepped-care recommendations that are tailored to the particular patient's needs.(78) Components of CC that have the greatest impact on successful outcomes include the use of standard screening among all patients in the system (e.g. clinic) and administration of psychological treatments.(79) CC has been applied to the treatment of OUD in open trials of non-pregnant patients (80, 81) and pregnant women.(68) It increased treatment initiation, engagement and use of psychotherapy among non-pregnant patients.(82) To our knowledge, there are no controlled or efficacy trials using CC for treatment of OUD in pregnancy. However, the model was felt to be sufficiently strong for MA to promote a statewide program for **Federally Qualified Health Centers** (**FQHCs**) in order to enhance the capacity of primary care providers to use buprenorphine.(3) As implemented in 14 primary care centers, the "Massachusetts Model" is slightly different than CC for depression outlined above and includes: (1) patients are systematically (screened) for opioid use and an OUD by a care manager; (2) the care manager assists with assessment and enrolls patients into a registry that includes symptom data that are collected longitudinally; (3) the care manager provides education, some behavioral treatment and care coordination; (4) the care manager assists with induction (treatment initiation) and follow-up (**urine toxicology screens**; **UDS**). In this way, the care manager assists the physician with time burdens and offers additional effort to tailor treatments and referrals to the needs of the patient. The Massachusetts Model does not include a psychiatrist.

Project ECHO (Extension for Community Healthcare Outcomes): This model was developed to build capacity to treat chronic, complex health conditions in rural and underserved communities that lack ready access to clinical specialists.(83, 84) Developed at the University of New Mexico Health Sciences Center to train primary care clinicians to treat patients with hepatitis C in rural communities (84, 85), it is now widely replicated across the country and around the world. Project ECHO uses a virtual hub-and-spoke educational model that links primary care clinicians with specialists through a real-time learning model made possible by inexpensive videoconferencing technology.(84, 86) Unlike traditional telemedicine, the ECHO model results in "force multiplication," (87) where a few specialists mentor many providers, who in turn provide enhanced care for large numbers of patients that would otherwise have limited access to specialty care. Project ECHO has been broadened to address many other conditions, such as HIV,(88, 89) mental illness,(90, 91) chronic pain,(92, 93) diabetes,(94) and OUD; it has been found to be an effective and potentially cost-saving model.(86)

In 2005, the **Integrated Addictions and Psychiatry (IAP)** ECHO clinic was developed to treat substance use and psychiatric disorders in New Mexico.(95) Supporting the availability of MAT with buprenorphine was a major focus of the IAP ECHO clinic. Support consists of video-conferencing and presentations of cases reviewed by the practitioners in a network of centers, “spokes,” that are curated by an expert in a “hub” (usually located in an academic medical center) who is available remotely. A typical ECHO session consists of 1) introductions of participants; 2) a brief didactic session, usually a 30-minute presentation on substance use or mental health; and 3) discussion of case presentations submitted by participants in advance. A community partner of ours would provide ECHO and would modify this by adding time during the first few months for education and training. The IAP program has contributed to training the largest number of buprenorphine-wavered physicians in rural areas of New Mexico, from <20 when the program started to 140 as of 2014.(95) The focus on OUD and buprenorphine training and support enhanced the capacity to treat OUD in New Mexico. While we know capacity to treat increased, there are no trials evaluating the efficacy of ECHO for the treatment of OUD in pregnancy compared to other methods.

2.3 RISK/BENEFIT ASSESSMENT

2.3.1 KNOWN POTENTIAL RISKS

The research procedures in this study are associate with minimal risk to the participants.

Stigma: The stigma associated with admitting use of substances in pregnancy is a problem and can deter women from seeing both prenatal care and treatment for an OUD. As well, practitioners may have negative attitudes towards patients with an OUD and be reluctant to offer them care. They may also be unwilling to manage women who require opioid agonist treatment and insist on abstinence, which may not be possible for some women. These issues will need to be addressed via broader education of a range of stakeholder groups.

Legal Concerns: Some states have mandatory reporting of substance use in pregnancy while others do not require notification unless the mother or baby tests positive for substances at delivery. Patients may not be willing to use prenatal care or participate because they may be concerned with potential legal consequences and involvement of Child Protective Services if they disclose opioid use. Obstetrical programs may also have concerns about reporting requirements and the burden this places on practitioners. This issue of reporting will need to be handled sensitively and in collaboration with patients so that women do not feel punished because they are trying to engage in treatment. We will include training for sites in both conditions on non-judgmental ways to handle reporting requirements. We will also provide guidance on complying with the laws. In qualitative work, we will explore the impact of reporting requirements on treatment and on participants’ willingness to receive treatment.

2.3.2 KNOWN POTENTIAL BENEFITS

Impact on Health Decisions

There is a dire need to establish care delivery systems that can address the opioid crisis in pregnant women. While there are several “ideal” models, a helpful model is one that could be implemented in obstetrical settings alongside prenatal care and would help pregnant women minimize their use of opioids and other substances. Both models outlined above have strengths and weaknesses in this regard. ECHO is scalable and as evidenced by the dramatic success in New Mexico, shows great promise to train obstetrical providers. The development of a “learning community” through the group case

presentation process, is a very powerful way in which to share knowledge and enhance therapeutic confidence among providers. This can go beyond clinical issues of medication initiation and titration to include ways to improve referral systems. Depending upon the commitment of the practice and their willingness to share resources, multiple individuals can be trained, and this can include physicians and support staff. The potential down-side of ECHO is the time-crunch physicians experience. While each practice can dedicate staff duties as it would desire, there is no explicit CM role in the ECHO model. In this sense, ECHO may have a greater impact in settings where some extender activities are already available (e.g. social worker services, recovery coaches, advanced practice nurses) although this is not clear. In addition to extender concerns, members of the team in the practice who plan to participate in ECHO (in this case physicians and nurses), must make time and be available to attend the ECHO sessions and to share case information with their colleagues. Availability can be a challenge in busy obstetric settings not only because of issues of patient flow but because of obstetrical emergencies.

As noted, CC has the benefit of a CM. The CM is trained in supporting behavioral techniques such as recovery coaching. The down side of CC is that it is potentially more labor intensive--the CM must be willing to follow a group of patients rather closely and this can take time. CC can also be costlier than ECHO and ultimately practices must be willing to dedicate resources to the CM. Medicare has made the costs of a CM billable and MA Medicaid has started to allow providers to bill for recovery coaching. Medicaid in CT does not allow practitioners to bill for these services but a study such as ours can inform policy makers.

The qualitative work proposed in this application can be very helpful. Infrastructure burdens can be explored. We will also qualitatively and quantitatively measure physicians' attitudes, perception of support, and perception of relationship with patients to enrich our understanding of clinicians' needs. Such results of this study may be helpful to payers and providers.

Impact on Questions that Affect Outcomes of Interest to Patients

Although the study compares delivery models for pregnant women with OUD, it is also poised to address many outcomes that are of interest to patients. First and foremost, our primary aims were developed with our community partners who felt treatment availability, engagement and retention were critical. Also, of interest to patients is their empowerment to make or share medical decision, including whether to elect MAT or not (see above). The study would also address domains that are important to patient stakeholders including: 1) communication between providers and patients; 2) recovery-related outcomes (role satisfaction, confidence in managing addiction); 3) emotional well-being; and 4) birth outcomes.

2.3.3 ASSESSMENT OF POTENTIAL RISKS AND BENEFITS

<u>Potential Risks</u>

Patient participants may experience distress while completing standardized assessment surveys, with feelings of guilt about substance use during pregnancy; anxiety and worry of adverse outcomes for themselves and their infants; and fear of public disclosure and potential legal consequences. The research team includes 4 psychiatrists who will be available to provide support for women who experience any psychological distress. We will reinforce the notion that all of the usual care available on site will be available to all participants. We will provide referrals as needed.

There is risk associated with a breach of confidentiality, and the release of unwanted information to persons outside the research team. There are also risks related to reporting requirements for substance

use in pregnancy (MA) or after delivery (all sites). This risk is present whether or not a woman participates in the study. We will follow usual clinic procedures for reporting.

There is a risk of clinical worsening (e.g., suicidal ideation, out of control substance use) in either groups that is common in this population.

Protection Against Risks

To minimize risks associated with study participation, we will take the following steps: First, designated staff person or care managers will tell potential participants that some assessment questions focus on sensitive topics and they can always choose not to answer a specific question. If a participant is upset by survey content, or develops suicidal ideation, the care manager can locate appropriate clinic staff or study staff to assist with the situation. We will train research staff to identify severe psychological distress and under those circumstances will inform a senior member of the research team to determine the safest course of action. We will also include a rescue protocol for worsening opioid or other substance use or suicidal ideation. This may include inpatient or partial hospitalization. Study faculty are experienced in the management of such circumstances with patient safety of paramount importance. We will tell participants that their clinical data are confidential and protected under HIPAA. We will link research data only by a participant ID number. We will collect survey data by computer using REDCAP®, a secure, encrypted program that affords additional protection of confidentiality for study participants. To further protect pregnant women participating in this research, we will obtain a Federal Certificate of Confidentiality. In addition, women will be told their decision to participate in the study is voluntary. They can choose not to participate or they can choose to withdraw from the study at any time, and it will not adversely affect their care at the clinic.

Benefits

It is critical to help pregnant women avoid use of illicit opioids as well as other substances that can negatively affect her health and contribute to adverse birth outcomes and longer-term problems with her offspring. This comparative effectiveness trial will compare models of care that may help pregnant women with OUD by providing onsite OBOT and counseling and it may help pregnant women for OUD in the future to tests these models of care.

Use of Collaborative Care with the obligatory longitudinal symptom monitoring, the provision of supportive psychological and psychiatric treatment, and the option of MAT shows promise to: reduce the maternal and fetal consequences of non-prescribed or illicit opioid use, minimize co-use of additional harmful substances such as cocaine and benzodiazepines, and improve maternal functioning at delivery and 3-months post-delivery. The condition of telesupport and a learning community is also poised to enhance the likelihood of buprenorphine prescribing and the care of pregnant women with an OUD. Patients, clinicians, payers and policy makers will benefit from a comparison of these two models.

3 OBJECTIVES AND ENDPOINTS

Our primary aims are:

1) To determine differences in **engagement and retention in OUD treatment** (MAT and/or non-pharmacological care) between participants who receive care from a center that uses a CC vs an ECHO support model. We hypothesize that the benefit from a CM and proactive monitoring approach will lead to greater treatment engagement and retention among patient participants.

H_{1a}: Participants with an OUD are more likely to be *engaged* in OUD treatment if the center is randomized to the CC vs the ECHO model of support.

H_{1b}: Participants with an OUD treated in CC centers are more likely to be *retained* OUD treatment than those treated in a center randomized to the ECHO model of care.

2) To determine differences in patient-reported outcomes between the two treatment models. For this domain we will use the **Patient Activation Measure (PAM)** as the sentinel measure, operationalized by assigning patients to one of four PAM levels based upon their scores.

H_2: Participants in the CC, compared to the ECHO condition, are more likely to increase at least one PAM level from baseline to delivery and 3-months post-partum.

Our secondary aims are:

1) To determine differences in **engagement and retention in MAT** between participants who receive care from a center that uses a CC vs an ECHO support model. We hypothesize that the benefit from a CM and proactive monitoring approach will lead to greater MAT engagement and retention among patient participants.

H_{1a}: Participants with an OUD are more likely to be *engaged* in **MAT** if the center is randomized to the CC vs the ECHO model of support.

H_{1b}: Participants with an OUD treated in CC centers are more likely to be *retained* in MAT for an OUD than those treated in a center randomized to the ECHO model of care.

2) To determine differences in birth outcomes between participants who receive care from a center that uses a CC vs. and Echo support model. For this domain we will examine **birth weight**.

H_2: Babies born to mothers treated in CC centers will have higher birth weights on average compared to babies born to mothers treated in ECHO centers.

3) To determine differences in obstetrical providers' work satisfaction among the two treatment models according to the **Physicians Worklife Survey.**

$H_{3:}$ Obstetrical providers randomized to the CC compared to the ECHO condition will score higher on subscales (autonomy, relationship with patients, patient care issues and relationship with staff) of the Physicians Worklife Survey.

Primary, secondary and exploratory outcomes are listed in the table below:

**Outcome**	**Type of Outcome**	**Assessment Tool**	**Assessment timepoints**	**Sample size**
Treatment engagement	Primary	Medical records and study database	Prenatal treatment period	480
Treatment retention	Primary	Medical records and study database	Birth and 3-months post-partum	480
Patient Activation	Primary	Patient Activation Measure	Baseline, 24 weeks ,36 weeks and 3 months post-	480

			partum	
#/% initiated onto MAT	Secondary	Medical records and study database	Prenatal treatment period	480
#/% retained on MAT	Secondary	Medical records and study database	Birth	TBD
Birth weight	Secondary	Medical records and study database	Birth	480
Physician Work Satisfaction	Secondary	Physician Work Life Survey	Before first participant enrollment and after last participant enrollment	12
#/% offered MAT	Exploratory	Medical records and study database	Prenatal treatment period	480
#/% continuing MAT at 3 months postpartum	Exploratory	Medical records and study database	3-months post-partum	TBD
#/% engaging in an opioid treatment program at 3 months post delivery	Exploratory	Medical Records and study database	3-months post-partum	480
#/% abstinent from illicit opioids or misuse or prescription opioids	Exploratory	a. TLFB<br>b. Urine Drug screens	a. monthly<br>b. each prenatal visit and birth and post birth	480
#/% with concurrent substance use	Exploratory	a. TLFB<br>b. Urine Drug screens	a. monthly<br>b. each prenatal visit and birth and post birth	480
Low birth weight	Exploratory	Medical records and study database	birth	480
Resuscitation at delivery	Exploratory	Medical records and study database	birth	480
Fetal demise	Exploratory	Medical records and study database	birth	480
Preterm birth	Exploratory	Medical records and study database	birth	480
Duration of hospitalization	Exploratory	Medical records and study database	Hospital discharge	480
Shared Decision Making	Exploratory	SDM-Q-9	Baseline, 24 weeks and 36 weeks	480
Patient-Physician Interaction	Exploratory	PEPPI-5	Baseline, 24 weeks, 36 weeks and 3 months post-partum	480

Clinician/Patient therapeutic relationship	Exploratory	Kim Alliance Communication (KAC)	Baseline, 24 weeks, 36 weeks and 3 months post-partum	480
Satisfaction with roles and activities	Exploratory	PROMIS Emotional Short Form 4a	Baseline, 24 weeks, 36 weeks and 3 months post-partum	480
Depression	Exploratory	EPDS	Baseline, 24 weeks, 36 weeks and 3 months post-partum	480

4 STUDY DESIGN

4.1 OVERALL DESIGN

Yale will lead this study to compare the effectiveness of two models of support for reproductive health clinicians who will provide prenatal and addiction care for pregnant and postpartum women with an opioid use disorder in obstetrical centers in Connecticut and Massachusetts. The two models being compared are: 1) a collaborative care (CC) approach, based upon the Massachusetts Office-Based-Opioid Treatment Model, that would provide onsite training, and support to providers through the use of a care managers; and 2) a tele-support approach modeled on the Project Extension for Community Healthcare Outcomes (ECHO), a remote education model that provides mentorship and guided practice and participation in a learning community via video conferencing. Twelve obstetrical centers who currently do not have a system in place to treat pregnant women with OUD will be recruited and their characteristics matched. The unit of randomization is the obstetrical center. We will randomly assign one center in each pair to either the CC or ECHO model, 6 sites to each model of support. A statistician, blinded to the identities of the clinics, will assign a number to each clinic using a random number generator; within each matched pair, the clinic with the lower number will be assigned to CC and the other will be assigned to ECHO. Among the centers, we would recruit 480 women in total, 240 women from the CC sites and 240 from the ECHO sites, about 40 per site among the 12 sites.

Recruitment of patient participants will be facilitated by universal screening by practice sites and referral to the study if appropriate. Screening for opioid misuse will occur at prenatal visits as recommended by the American College of Obstetricians and Gynecologists. Clinicians will follow up on affirmative answers and discuss study participation with them. A designated staff person (DSP) will hand potentially eligible women a tablet that includes screening questions and a patient consent document. After reviewing the consent form, and having any questions answered and signing the consent form, study participants will then complete a baseline assessment that includes demographic information and questions about pregnancy dates and a checklist that would indicate an opioid use disorder (OUD) (via a tablet computer). After completion of these questions, the tablet and DSP will determine whether the woman remains eligible for participation and will be enrolled. The participant will be assigned a study identification number. She will then complete additional questions that are part of the baseline assessment for Visit 1 (see Table 1). After enrollment, a urine drug screen (UDS) will be obtained at each prenatal visit (the urine will be collected as part of the regular prenatal visit and tested for the presence

of illicit and licit opiate drugs). Study sites will be asked to report results of UDS by completing a form that is entered into REDCap. Participants will also be contacted monthly by a Yale research assistant (who is blind to condition) to complete a **Time Line Follow-Back** (**TLFB**) and the **Treatment Utilization Form** that includes questions about the quantity and frequency of all substances used and treatment received. Study participants will complete additional self-report questionnaires at the following assessment points: enrollment, 26 weeks and 36 weeks (+/- 4 weeks), and at 3M post-partum. Data will be collected using a tablet provided by the DSP. In addition, maternal/infant medical record data will be extracted by the DSP and provided to the Yale and UMass data collection teams. These data will include maternal, fetal and neonatal outcomes.

A subset of patient participants in the trial will be given the opportunity to participate in a separate qualitative interview. These interviews will collect data on patent level of satisfaction with MAT experiences at the clinics.

The experiences of physicians involved in the project will also be assessed. At least one provider from each practice (minimum of 12) will also be invited to participate in qualitative interviews to assess their perceptions of the implementation of MAT within their practice including barriers and facilitators to implementation. All providers who participated will be asked to complete brief forms asking about workload and clinical support.

4.2 SCIENTIFIC RATIONALE FOR STUDY DESIGN

Given the scope of opioid crisis, the need for treatment outpaces the capacity to provide it.(56) Increasing the cadre of buprenorphine providers can help address this but there are barriers including: lack of patient and physician interest,(57-59) lack of expertise and education,(57, 58, 60, 61) limited physician time and resources, (57, 62, 63) stigma towards patients with OUD,(58, 61) concern about abuse or diversion,(96, 97) lack of institutional support,(59, 62, 96) cumbersome regulations,(60, 65) and insurance barriers (e.g. insufficient rates of reimbursement).(57, 61, 63, 96) Provider barriers may be particularly problematic for pregnant women with an OUD.(2) A possible solution is to combine addiction care (including MAT) with prenatal care.(50, 67-69) Some existing programs provide “women-centered” care to patients with an OUD, including prenatal care, MAT, care coordination, behavioral health services, family planning and breastfeeding support. (18, 67, 70) Such programs are more likely than non-women-centered programs, to dose opiate agonist treatment (buprenorphine) to fit the physiology of pregnancy, enhance the utilization of long-acting contraception and have better rates of breastfeeding continuation.(67) Despite the potential benefits of a program that provides conjoint prenatal and addiction care, **there is a major gap in knowledge regarding the best models for supporting centers that would like to provide such women-centered care to women with an OUD treated in obstetrical settings.** The models of care we propose to test address many of these concerns, although they differ in their potential to affect many of them: 1) we know that the cadre of physicians, including obstetricians, who are trained to provide MAT is insufficient; 2) we also know that among those who have been waivered to use MAT, many obstetrical providers do not provide MAT; 3) indeed, many obstetricians identify lack of support for their provision of addiction care, especially given the complex needs of this patient population (Society of Maternal Fetal Medicine Workshop Report, forthcoming, 2018) as the reason they do not provide MAT. There is a need to compare different types of support to obstetrical practices that would allow them to deliver addiction care, in a women-centered manner.

Comparative Results on Maternal and Fetal Outcomes and Patients’ Perception of Care **(RQ-3)**: If addiction treatment, including MAT, is provided through obstetrical practices, it needs to be done with

APPROVED BY THE YALE UNIVERSITY IRB 12/9/2020

fidelity and in a way that meets patients' needs. Only a few studies have assessed possible differences in maternal and fetal well-being for gravidas treated in programs that provided combined vs separate prenatal care and addiction treatment.(67) Some cohort studies provided information on fetal outcomes in centers that provide combined care.(18, 20) We were unable to identify studies or trials that compared different models of combined prenatal and substance use care for pregnant women with OUD. Gaps in this literature are many, some of which include: rates of engagement in addiction treatment; level of retention in prenatal care and addiction treatment; rates of adverse birth outcomes; utilization of MAT as part of addiction treatment; rates of continuing MAT during the postpartum period; treatment of concurrent substance misuse and psychiatric conditions.

As well, but equally important is the lack of knowledge regarding women's experiences when they receive treatment under different models of combined care. Some of the domains of importance include patient's engagement in care, knowledge of care, confidence in managing pregnancy and their addiction, alliance with their providers, emotional well-being, ability to fulfill important roles in life and retain custody of their children if they wish to do so. A trial of different models of care can provide information to fill in these important gaps.

4.3 JUSTIFICATION FOR INTERVENTION

Collaborative Opioid Prescribing Model (Massachusetts Model):

This model is based upon CC, a system developed initially to enhance the capacity for treatment of depression in primary care settings.(73) The model has since been refined (74, 75) and tested for treatment of other disorders; it has been deployed in additional venues including obstetrical-gynecological settings.(76, 77) CC, as articulated by the University of Washington, includes several components: (1) patients are systematically evaluated (screened) for medical and behavioral health needs; (2) a CM assists with assessment and enrolls patients in a registry that includes data on symptoms that are collected longitudinally; the CM provides some proactive behavioral treatment and care coordination; 3) a psychiatrist reviews cases and assists the primary physician in providing stepped-care recommendations that are tailored to the particular patient's needs.(78) Components of CC that have the greatest impact on successful outcomes include the use of standard screening among all patients in the system (e.g. clinic) and administration of psychological treatments.(79) CC has been applied to the treatment of OUD in open trials of non-pregnant patients(80, 81) and pregnant women.(68) It increased treatment initiation, engagement and use of psychotherapy among non-pregnant patients.(82) To our knowledge, there are no controlled or efficacy trials using CC for treatment of OUD in pregnancy. However, the model was felt to be sufficiently strong for MA to promote a statewide program for **Federally Qualified Health Centers** (**FQHCs**) in order to enhance the capacity of primary care providers to use buprenorphine. (3) As implemented in 14 centers with primary care patients, the "Massachusetts Model" is slightly different than CC for depression outlined above and includes: (1) patients are systematically (screened) for opioid use and an OUD by a CM; (2) the CM assists with assessment and enrolls patients into a registry that includes symptom data that are collected longitudinally; (3) the CM provides education, some behavioral treatment and care coordination; (4) the CM assists with induction (treatment initiation) and follow-up (**urine toxicology screens**; **UDS**). In this way, the CM assists the physician with time burdens and offers additional effort

Figure 1: Collaborative Care Model

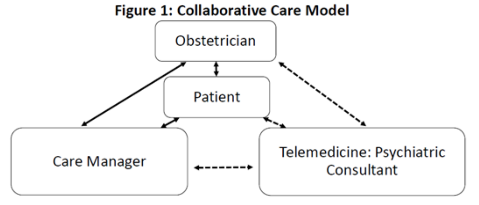

APPROVED BY THE YALE UNIVERSITY IRB 12/9/2020

to tailor treatments and referrals to the needs of the patient. The Massachusetts Model does not include a psychiatrist.

Project ECHO (Extension for Community Healthcare Outcomes):

This model was developed to build capacity to treat chronic, complex health conditions in rural and underserved communities that lack ready access to clinical specialists.(83, 84) Developed at the University of New Mexico Health Sciences Center to train primary care clinicians to treat patients with hepatitis C in rural communities(84, 85), it is now widely replicated across the country and around the world. Project ECHO uses a virtual hub-and-spoke educational model that links primary care clinicians with specialists through a real-time learning model made possible by inexpensive videoconferencing technology.(84, 86) Unlike traditional telemedicine, the ECHO model results in "force multiplication,"(87) where a few specialists mentor many providers, who in turn provide enhanced care for large numbers of patients that would otherwise have limited access to specialty care. Project ECHO has been broadened to address many other conditions, such as HIV,(88, 89) mental illness,(90, 91) chronic pain,(92, 93) diabetes,(94) and OUD; it has been found to be an effective and potentially cost-saving model.(86)

In 2005, the **Integrated Addictions and Psychiatry (IAP)** ECHO clinic was developed to treat substance use and psychiatric disorders in New Mexico.(95) Supporting the availability of MAT with buprenorphine was a major focus of the IAP ECHO clinic. Support consists of video-conferencing and presentations of cases reviewed by the practitioners in a network of centers, "spokes," that is curated by an expert in a "hub" (usually located in an academic medical center) who is available remotely. A typical ECHO session consists of 1) introductions of participants; 2) a brief didactic session, usually a 30-minute presentation on substance use or mental health; and 3) discussion of case presentations submitted by participants in advance. A community partner of ours would provide ECHO and would modify this by adding time during the first few months for education and training. The IAP program has contributed to training the largest number of buprenorphine-wavered physicians in rural areas of New Mexico, from <20 when the program started to 140 as of 2014.(95) The focus on OUD and buprenorphine training and support has enhanced the capacity to treat OUD in New Mexico. While we know capacity to treat has increased, there are no trials evaluating the efficacy of ECHO for the treatment of OUD in pregnancy compared to other methods.

4.4 END-OF-STUDY DEFINITION

A participant is considered to have completed engagement in the study if she has completed the baseline assessment and has had at least one visit per month for 3 months with the OUD care treatment provider. Participants who leave the study before their 3-month postpartum visit will still be included in analysis and will be an important part of the outcome data. A participant is considered to have been successfully retained in the study if she has completed engagement and remained in treatment through the 3-month postpartum follow-up visit.

The end of the study is defined as completion of the 3-month post-partum visit shown in the Schedule of Activities (SoA), **Section 1.3**.

5 STUDY POPULATION

In this study design both the clinician and the patient- participant are contributing data toward the study aims. Physicians and patient – participants will both provide informed consent as appropriate.

Physician – participants will need to be a member of a practice or clinic that was included in the clinical trial which has a CM, DSP or obstetrician, who will be providing care and be willing to provide informed consent.

5.1 INCLUSION CRITERIA

In order to be eligible to participate in this study, a participant must meet all of the following criteria:

1. Provision of signed and dated informed consent form
2. Females ≥ 18 years old
3. Documented pregnancy in the medical record of less than 34 weeks gestation
4. Delivery date no later than March 15, 2023
5. Willingness to adhere to the study schedule
6. Confirmed opioid use disorder by the DSM-5 Opioid Use Disorder questionnaire
7. Ability to communicate in English

In order for providers who are not principal investigators to participate they must meet the following criteria:

1. Provision of verbal consent
2. Willingness to adhere to the study schedule based upon site randomization
3. Speak English

5.2 EXCLUSION CRITERIA

A patient participant who meets any of the following criteria will be excluded from participation in this study:

1. Experiencing cognitive or emotional impairment that precludes them from providing informed consent
2. Current hospitalization, incarceration, or institutionalization (if women present for care after institutionalization, they will be eligible to participate), or a court case pending such that incarceration during the study treatment period would be likely
3. Have plans to move from the area in which they are currently seeking their obstetrical care within the study period (which will be 44 weeks at most)

Providers will be excluded who:

1. Have no exposure to one of the two models of support that are being compared
2. Are a temporary worker (e.g. student or per diem for a short period of time, although long-term per diems will be allowed)
3. Are not willing to undergo training for buprenorphine administration

5.3 LIFESTYLE CONSIDERATIONS

During this study, participants are asked to:

- Come to visits as scheduled
- Continue or begin any behavioral or medication assisted OUD treatment (MAT).
    - Note the MAT status of patients can vary: women may choose to receive buprenorphine, they may receive methadone from anther provider, or they may choose to receive no MAT. We would also allow women to change their decisions about MAT; we would like to respect their choice and they may decide later to engage in MAT. We will also include women who are on methadone since they may prefer to switch to buprenorphine or no MAT.

5.4 SCREEN FAILURES

Screen failures are defined as participants who consent to participate in this study but are not subsequently entered in the study due to not meeting one or more eligibility criteria. Screen failures may be rescreened if their status changes at any point during their prenatal care. Rescreened participants will be assigned the same participant number as for the initial screening.

5.5 STRATEGIES FOR RECRUITMENT AND RETENTION

Participants will be enrolled at 12 obstetrical centers that are matched on demographics of patient population. Within each pair of centers, each will be randomly assigned to the CC or ECHO condition. We will enroll 480 women in total, half in each treatment condition.

Women will be enrolled at each site by a designated staff person (DSP), most likely a nurse (at the CC practices it will be the CM). As recommended by ACOG,(33) the DSP will administer their standard opioid screening to all pregnant women at all sites. Women with a positive screen will be invited to participate. DSPs will then obtain written consent from patients who agree to participate in the trial. The DSM-5 checklist will be completed for the diagnosis of OUD. Women who meet criteria for OUD will be deemed eligible to participate in the trial. To minimize attrition, the DSP will then: 1) explain the importance of follow-ups, 2) rapidly schedule first visit with a provider associated with the study after confirmation of eligibility and consent, 3) collect at least 3 verified locators, 4) call women to remind them of their appointments, and will have contact with a significant other who can provide collateral information and maintain subject involvement. Investigators will monitor participants who drop out and explore with the staff reasons for the dropouts.

In addition to patient participants in the trial, qualitative interviews will be conducted with a subset of patient participants and physicians involved in the project. Patient participants will be contacted, consented and enrolled in this portion of the project by computer randomization.

Physicians will provide verbal consent form prior to participation.

Incentives:

Patients enrolled at both the CC and ECHO sites will be compensated up to $285. This would include $5 for Screening, $25 for Baseline, $15 for the initial phone interview, $5 for each following Timeline Follow-back phone interview, $25 for 26-week follow-up, $25 for 36-week follow-up and $50 for 3-Month post-partum follow-up.

Patients who enroll in the qualitative portion of the study would be paid up to an additional $50 for each interview (at 6 months of pregnancy and 3 months post-partum).

6 STUDY INTERVENTION(S) OR EXPERIMENTAL MANIPULATION(S)

6.1 STUDY INTERVENTION(S) OR EXPERIMENTAL MANIPULATION(S) ADMINISTRATION

6.1.1 STUDY INTERVENTION OR EXPERIMENTAL MANIPULATION DESCRIPTION

The sites randomized to CC will have 4-6 on-site support visits at the outset of the study. These visits will occur during the first 3 months of the project. During these visits, study staff will again review buprenorphine induction procedures (heretofore referred to as "initiation" rather than "induction") and dosing of buprenorphine as well as therapeutic behavioral treatments. We will provide the center with forms and educational materials to help dosing during initiation procedures. At the same time, we will set up visits for the CM to meet with a recovery coach trainer provided by our community partner, CCAR. We will review data collection materials and methods.

The CC team includes the nursing CM and obstetrical provider. The CM responsibilities include: 1) screening patients for an OUD; 2) entering CC participants into the patient registry; 3) providing education to participants about OUD (and psychiatric disorders such as depression if needed); 4) assistance in initiation procedures for patients who would like to receive buprenorphine;(3) 5) informing the obstetrical provider of a positive UDS and need to consider an increase in buprenorphine; if the patient is on methadone, the outside treatment program obtains a UDS and will make appropriate adjustments but the CM can provide support and will collect UDS as project data; 6) if the patient is not receiving MAT, a discussion about starting MAT or psychological treatment; 7) following a safety transfer protocol, a manual SOP provided to the sites by the Yale research team at the study start-up meeting; if the patient has 3 consecutive urines that are positive for illicit substances and the dose of buprenorphine is maximized (24 mgs/day), or if there is definitive evidence of diversion, or the participant is receiving any form of MAT and has dangerous level of cocaine, opioid or methamphetamine use), or is suicidal; 8) recovery coaching. For research purposes, the CM will confirm informed consent, collect and enter results of the UDS into a database. At enrollment and at 26 and 34 weeks' gestation and 3 months postpartum she will ask participants to complete an assessment battery of self-reported measures.

The Weitzman Institute (WI), a community partner and provider for the ECHO condition, is a research and innovation center embedded within one of the largest FQHCs in the country, Community Health Center, Inc.(CHC). It is focused on improving care for medically underserved and special populations. Since 2013, CHCI/WI has provided education and technical assistance through its established video conference and online learning platforms to over 800 primary care providers and over 200 care team members who treat patients with chronic pain and opioid addiction in 28 states plus Washington DC and Puerto Rico. CHC/WI partners with national experts in pain care, substance abuse disorder, and MAT to create and deliver a robust training program for clinicians across the country through Project ECHO ®. The WI will work with our study team (Drs. Fiellin, Callaghan, Lipkind, Yonkers, Byatt and Forray) to develop and staff the sessions. Data collection will mirror the CC condition and conducted by a person designated by the practice, designated study person (DSP).

6.1.2 ADMINISTRATION AND/OR DOSING

CC- Obstetricians, who are X-waivered will see all participants; initially they will discuss preferences for addiction treatment (buprenorphine, methadone or no MAT) with the participant. For gravidas who elect treatment with buprenorphine, obstetricians will adjust the dose of buprenorphine until patients are stabilized. They will see stabilized buprenorphine, methadone and no MAT participants as needed for OUD care (typically every 1-2 weeks for those who are prescribed buprenorphine; other women it may be more or less frequently), and typical prenatal care. The obstetrician may benefit from ongoing support via e-mail and video conference available at no cost from the SAMHSA-funded Prescriber Clinical Support System for MAT (PCSS-MAT). Practices may elect to have more than one obstetrician or APRN complete the X-waiver process so that they can provide back-up care during the absences of the primary study obstetrician.

The care team (CM, obstetrician) will meet at least monthly but more frequently if the case load is in need of more frequent meetings, and will review the CC participants' scores, UDS and needs. The obstetrician can refer a participant to psychological or psychiatric treatment offsite and/or could be contacted by the CM for support, recovery coaching and to troubleshoot issues with attendance or other issues.

ECHO- The practice members who participate in ECHO will include obstetricians and nurses as well as other members of the care team who wish to join. ECHO sessions are recorded and available asynchronously for review if providers are unavailable. Providers participating in Project ECHO sessions are given access to a password-protected website containing the recorded sessions, a discussion board, and resource library. CME credits are available for each session and are provided through the American Academy of Family Physicians (AAFP) and can motivate providers to attend.

A DSP will obtain consent, check the UDS and enter results of the UDS into a database. At enrollment, 26 and 34 weeks' gestation and at 3-month postpartum visit, this person will give participants a tablet with an assessment battery of self-reported measures.

Thus, essential differences between models are: 1) CC has designated roles for the CM (a participant registry and psychotherapeutic support via recovery coaching); 2) ECHO has ongoing support from a learning community tailored to the needs of the practices.

6.2 FIDELITY

6.2.1 INTERVENTIONIST TRAINING AND TRACKING

After clinical sites have been finalized, they will be randomized to a condition and start-up meeting for sites in each condition will be scheduled. Prior to the meeting we will ask physicians (who have not completed the waiver-2000 process) at each site to complete 4 hours of on-line training for the waiver-2000 process (Drug Addiction Treatment Act of 2000 (DATA 2000) buprenorphine waiver trainings). The additional 4 hours will be completed at the start up meeting. At the meeting, we will provide education about OUD, especially in pregnancy. We will review the study protocol, discuss site concerns and challenges and plans for either the on-site visits or ECHO sessions.

The WI will train faculty to the ECHO model and deliver the sessions in Year 1 of the project. The sessions will occur more frequently during this time (2-4 times per month) since this is the time when the most intensive education is needed. After that, they will receive monthly sessions during Years 2. In addition to ECHO, providers in this condition will have access to the educational materials that the CC condition has. We will inform them of the PCSS-MAT that offers support to clinicians who prescribe

buprenorphine. While the roles in the ECHO condition are not prescribed as they are in CC, the practice may decide to have a specific nurse in charge of many of the tasks of the CM, but this is not obligatory and we will not be providing the onsite training. We recognize that sites will vary in terms of the availability and proximity of psychiatric and psychological services. To help neutralize differences we will include educational material that discusses supportive counseling and motivational interviewing for substance use disorders. We will present this material to the learning community as an educational component prior to discussion of cases during ECHO sessions. As well, part of the ECHO curriculum will discuss building a referral network and enhancing referral to counseling and the use of motivational interviewing.

6.3 MEASURES TO MINIMIZE BIAS: RANDOMIZATION AND BLINDING

Matching of Clinics- Participating clinics will be matched as closely as possible based on state, size and academic vs. non-academic / community hospital to lessen site interactions with the data.

Randomization of Clinics- Within each matched pair, one clinic will be randomly assigned to the CC condition and one will be assigned to the ECHO condition. Randomization will occur by assigning a number to each clinic using a random number generator; within each matched pair, the clinic with the lower number will be assigned to CC and the other will be assigned to ECHO.

Blinding of Research Staff- The statistician will be blinded to the identities of the clinics when assigning treatment allocation during randomization. Research staff who will contact the participants to conduct the monthly TLFB will be blinded to the treatment condition. In addition, the research staff who extract the maternal and infant outcome data

6.4 STUDY INTERVENTION/EXPERIMENTAL MANIPULATION ADHERENCE

Our primary outcomes are percent of pregnant women who are i) successfully engaged at least one visit per month for 3 months) and ii) retained on MAT and/or non-pharmacological care (for the purposes of this protocol, these visits will be with billable opioid use treatment providers)at delivery and through 3 months postpartum . The percent will be determined via annual baseline assessments of women who would be eligible according to an estimation of the number of women in the practice who have urine tests positive for opioids (see section D.5.d.). We define unsuccessful retention according to a modification of Wilder, et al. (2015),(98) as women who enrolled in pharmacological or behavioral treatment for opioid misuse who stopped treatment with no plan for ongoing therapy or medication for two months (e.g. discharge, relapse and left treatment, lost to follow up with no discharge plan) during pregnancy, and the 3 months post-delivery. We add **percent engagement** as our outcome because a site may look successful in retention if they only engage a very motivated subset of eligible women in treatment yet are able to retain them. This would miss a proportion of women who may have complex illness and are more difficult to both engage and retain in treatment. We adopt a broader outcome that includes therapy rather than just initiation and continuation of MAT with input from our community partners who felt that allowing women to select either MAT and/or psychotherapy respects patient choices. We include **percent retained** because engagement is not sufficient to consider a treatment as successful. Exploratory analyses will compare the **number** of women engaged and retained in treatment. We include visits with the care manager as indicative of receiving behavioral therapy in the CC condition only if there is a specific focus on addiction treatment (e.g. recovery counseling) since that condition by default has on-site treatment available. For analysis, we would use two intervals: in

APPROVED BY THE YALE UNIVERSITY IRB 12/9/2020

pregnancy and then after delivery. Pregnancy and postpartum are very different time intervals and it is useful to differentiate them. We would then measure overall retention during the entire study period.

6.5 CONCOMITANT THERAPY

Medication assisted treatment (MAT) is a critical component of standard care for pregnant women with an OUD (35, 36) and is recommended by the **American College of Obstetricians and Gynecologists (ACOG)**(33) and other experts.(5, 37) It enhances adherence to prenatal care (38) and increases the likelihood that the mother will continue MAT after delivery, a period of high risk among perinatal women with OUD.(39) Initially MAT included methadone but now includes buprenorphine and naltrexone (not yet indicated for use in pregnancy), which can be prescribed in primary care settings.(40)

MAT status of patients can vary: women may choose to receive buprenorphine, they may receive methadone from anther provider, or they may choose to receive no MAT. We would also allow women to change their decisions about MAT; we would like to respect their choice and they may decide later to engage in MAT. We will also include women who are on methadone since they may prefer to switch to buprenorphine or no MAT.

Outside behavioral therapy- Patients can be receiving behavioral therapy at the onset of the study or choose to begin therapy while on the study. Concomitant therapy will be recorded in the eCRFs.

6.5.1 RESCUE THERAPY

All practices will have a rescue protocol. This includes guidance on substance use and on suicidal plan endorsement.

Rescue Protocol for Substance Use

1) Consider increasing buprenorphine dose beyond 24 mg per day for the following reason:
    a. Temporary elevation during third trimester to address physiologic changes in metabolism, if patient has been on 24mg per day prior to pregnancy and shows signs of withdrawal in the third trimester.

2) If 3 consecutive urines are positive for illicit opioids (or at least one urine positive for illicit opioids and one or more missed appointments at which additional urines would have been collected), and buprenorphine is maximized, consider transfer to a structured treatment program
    a. Structured treatment programs can include:
        i. methadone program or any outpatient opioid treatment program with methadone or buprenorphine along with multidisciplinary services and more supervision of medication prescriptions
        ii. inpatient substance use treatment
        iii. intensive outpatient treatment
    b. Alternatively, consider increasing adjuvant treatments such as groups, counseling, Peer Support, or Recovery Coaches.

APPROVED BY THE YALE UNIVERSITY IRB 12/9/2020

3) Consider a transfer of care to methadone if there is definitive evidence of diversion -for example as evidenced by 3 consecutive urine drug tests negative for buprenorphine

Rescue Protocol for Suicidal Ideation

1) If a participant endorses greater than “0” on the Edinburgh Postnatal Depression Rating Scale question # 10, the tablet will be programmed to have a hard stop. The participant will be instructed to show the scale to their nurse or doctor so that sites can employ their usual procedures to further assess suicide plans or intent and manage the patient (eg. assessment by a behavioral health care provider or referral to the emergency department)
2) Study personnel will work with sites that do not have a pre-existing suicide protocol to develop such a plan

7 STUDY INTERVENTION/EXPERIMENTAL MANIPULATION DISCONTINUATION AND PARTICIPANT DISCONTINUATION/WITHDRAWAL

7.1 PARTICIPANT DISCONTINUATION/WITHDRAWAL FROM THE STUDY

Participants are free to withdraw from participation in the study at any time upon request.
An investigator may discontinue a participant from the study for the following reasons:

- Lost-to-follow up; unable to contact subject
- Any event or medical condition or situation occurs such that continued collection of follow-up study data would not be in the best interest of the participant or might require an additional treatment that would confound the interpretation of the study
- The participant meets an exclusion criterion (either newly developed or not previously recognized) that precludes further study participation

The reason for participant discontinuation or withdrawal from the study will be recorded in the CRF.

7.2 LOST TO FOLLOW-UP

A participant will be considered lost to follow-up if he or she fails to return for at least three visits scheduled visits and study staff are unable to contact the participant after at least 3 attempts.

The following actions must be taken if a participant fails to return to the clinic for a required study visit:

- The site will attempt to contact the participant, reschedule the missed visit within three weeks, counsel the participant on the importance of maintaining the assigned visit schedule and ascertain if the participant wishes to and/or should continue in the study
- Before a participant is deemed lost to follow-up, the investigator or designee will make every effort to regain contact with the participant including email, text and telephone contact (depending upon patient preference). If a participant does not respond to email, we will attempt 3 telephone calls and, if necessary, a certified letter to the participant’s last known mailing address or local equivalent methods). If the participant responds but does not respond again, this will reset the attempts to contact unless she has asked us not to contact her. These contact attempts will be documented in the participant’s medical record or study file.

- Should the participant continue to be unreachable, he or she will be considered to have withdrawn from the study with a primary reason of lost to follow-up

8 STUDY ASSESSMENTS AND PROCEDURES

8.1 ENDPOINT AND OTHER NON-SAFETY ASSESSMENTS

8.1.1 SCREENING & BASELINE PROCEDURES

- *Substance Misuse Screen* – All patients seen at the participating clinical are asked substance misuse screening questions as part of their standard or care. Patients who answer affirmatively will be approached for consent.

- *Demographics*- Consented patients will have demographic data collected including information about pregnancy dates, medications and complications.

- *Concomitant Therapy*- Participants will be asked about any medications or other therapy they have received since their last visit. This information will be recorded in the eCRF.

- *Medical History*- Participants will be asked about their medical history. Medical history will also be collected through review of the patient's medical record if available.

- *Pregnancy Questionnaire*- The following information will be collected about the patient's current pregnancy; dates, medications and complications

- *Urine Drug Screening*- Urine collected at pre-natal visit will be tested for illicit drugs and licit opioids

- *DSM-5 OUD Checklist*- Patient's will complete the DSM-5 OUD checklist to confirm OUD.

- *Contact Information*- At least 3 verified locators will be collected for each participant. If available, one of these contacts will be a significant other who can provide collateral information and maintain subject involvement

- *Self-Assessments Including:*
    - *Patient Activation Measure (PAM)*
    - *Post Traumatic Stress Disorder Checklist for DSM-5 (PCL-5)*
    - *Fagerstrom Test For Nicotine Dependence (FTND)*
    - *HIV Risk Taking Behavior Scale (HRBS)*
    - *Shared-Decision Making Questionnaire-9 (SDM-Q-9)*
    - *Perceived efficacy in Patient-Physician Interactions Questionnaire (PEPPI)*
    - *Kim Alliance Scale-Communication subscale (KAS-CM)*
    - *PROMIS Emotional Short Form*
    - *Satisfaction with Roles and Activities*

   - *Edinburgh Postnatal Depression Scale (EPDS)*
   - *Stigma Related Rejection Scale*
   - *Adverse Childhood Experience Questionnaire*

8.1.2 STANDARD OF CARE PRENATAL VISIT

- *Assigned OUD Treatment*- Physicians and clinical staff will follow the treatment plan for their assigned group (CC or ECHO) at each patient visit.
- *Urine Drug Screening*- Urine collected at pre-natal visit will be tested for illicit drugs and licit opioids.

8.1.3 TIMELINE FOLLOW BACK VISITS

- *Timeline Follow Back*- Participants will also be contacted monthly by a research assistant (who is blind to condition) to complete a Time Line Follow-Back (TLFB) about the quantity and frequency of all substances used and treatment received.
- *Treatment Utilization Form*- Participants will be asked about any medications or other therapy they have received since their last visit.

8.1.4 WEEK 26 VISIT

- *Self-Assessments Including:*
   - *Patient Activation Measure (PAM)*
   - *Fagerstrom Test For Nicotine Dependence (FTND)*
   - *Shared-Decision Making Questionnaire-9 (SDM-Q-9)*
   - *Perceived efficacy in Patient-Physician Interactions Questionnaire (PEPPI)*
   - *Kim Alliance Scale-Communication subscale (KAS-CM)*
   - *PROMIS Emotional Short Form*
   - *Satisfaction with Roles and Activities*
   - *Edinburgh Postnatal Depression Scale (EPDS)*
- *Qualitative Interviews (sub group)*

8.1.5 WEEK 36 VISIT

- *Self-Assessments Including:*
   - *Patient Activation Measure (PAM)*
   - *Fagerstrom Test For Nicotine Dependence (FTND)*
   - *Shared-Decision Making Questionnaire-9 (SDM-Q-9*

- *Perceived efficacy in Patient-Physician Interactions Questionnaire (PEPPI*
- *Kim Alliance Scale-Communication subscale (KAS-CM)*
- *PROMIS Emotional Short Form*
- *Satisfaction with Roles and Activities*
- *Edinburgh Postnatal Depression Scale (EPDS)*

8.1.6 BIRTH

- *Birth Outcomes-* Birth outcomes will be collected through review of the patient's medical record including low birth weight, resuscitation at delivery, fetal demise, preterm birth, duration of hospitalization.

8.1.7 3 MONTHS POST-PARTUM VISIT

- *Self-Assessments Including:*
  - *Patient Activation Measure (PAM)*
  - *Fagerstrom Test For Nicotine Dependence (FTND)*
  - *Shared-Decision Making Questionnaire-9 (SDM-Q-9*
  - *Perceived efficacy in Patient-Physician Interactions Questionnaire (PEPPI)*
  - *Kim Alliance Scale-Communication subscale (KAS-CM)*
  - *PROMIS Emotional Short Form*
  - *Satisfaction with Roles and Activities*
  - *Edinburgh Postnatal Depression Scale (EPDS)*
  - *Stigma Related Rejection Scale*
- *Qualitative Interviews (sub group)*

PRE AND POST SUBJECT ENROLLMENT

Physicians will complete the Substance Abuse Attitudes Survey, the Physicians WorkLife Survey and the optional Qualitative Interview prior to first participant enrollment at the site and after the last participant enrollment at the site.

8.2 SAFETY ASSESSMENTS

Assessment of Adverse Events- Participants will be asked about any events that meet the definition of an AE listed below at each study contact. This information will be recorded in the eCRF.

8.3 ADVERSE EVENTS AND SERIOUS ADVERSE EVENTS

8.3.1 DEFINITION OF ADVERSE EVENTS

APPROVED BY THE YALE UNIVERSITY IRB 12/9/2020

This protocol uses the definition of adverse event from 21 CFR 312.32 (a): any untoward medical occurrence ***whether or not it is considered related to participation in the study***.

8.3.2 DEFINITION OF SERIOUS ADVERSE EVENTS

A serious adverse event is any adverse event that results in one of the following:

1. Results in death
2. Is immediately life-threatening
3. Requires in-patient hospitalization (other than for expected delivery or other anticipated obstetrical procedures unrelated to participation) or prolongation of existing hospitalization (other than for anticipated obstetrical reasons, e.g., cesarean section).
4. Results in persistent or significant disability or incapacity
5. Results in a congenital abnormality or birth defect
6. Is an important medical event that may jeopardize the subject or may require medical intervention to prevent one of the outcomes listed above

As with AEs, SAEs do not have to be related to participant in the study. For example, if the participant is in a car accident and is hospitalize, that is considered an SAE and should be recorded. It is important to note, that not all SAEs need to be report to IRBs. Please see details below.

8.3.3 CLASSIFICATION OF AN ADVERSE EVENT

8.3.3.1 SEVERITY OF EVENT

Adverse events will be graded according to the following guidelines.

- **Mild** – Events require minimal or no treatment and do not interfere with the participant's daily activities.
- **Moderate** – Events result in a low level of inconvenience or concern with the therapeutic measures. Moderate events may cause some interference with functioning.
- **Severe** – Events interrupt a participant's usual daily activity and may require systemic drug therapy or other treatment. Severe events are usually potentially life-threatening or incapacitating. Of note, the term "severe" does not necessarily equate to "serious".

8.3.3.2 RELATIONSHIP TO STUDY INTERVENTION/EXPERIMENTAL MANIPULATION

All adverse events (AEs) will have their relationship to study procedures, including the intervention, assessed by an appropriately-trained clinician based on temporal relationship and his/her clinical judgment. The degree of certainty about causality will be graded using the categories below.

- **Related** – The AE is known to occur with the study procedures, there is a reasonable possibility that the study procedures caused the AE, or there is a temporal relationship between the study procedures and the event. Reasonable possibility means that there is evidence to suggest a causal relationship between the study procedures and the AE.
- **Not Related** – There is not a reasonable possibility that the study procedures caused the event, there is no temporal relationship between the study procedures and event onset, or an alternate etiology has been established.

8.3.3.3 EXPECTEDNESS

APPROVED BY THE YALE UNIVERSITY IRB 12/9/2020

APPROVED BY THE YALE UNIVERSITY IRB 12/9/2020

Given the population of study participants the following AE are expected and will not recorded as part of the study. Examples of this include hospitalization for delivery with or without complications. Deliveries are expected events and adverse birth complications are not uncommon in this population. This relates to the substances that are misused and not to the randomization of support systems.

Women on medication for OUD commonly deliver babies that are small for gestational age or early and this can be due to the medication, the earlier misuse of an opioid or use/misuse of other substances. Provision of medication for an OUD is recommended treatment outside of the study.

Other common and expectable events include delivery of infants who require admission to a neonatal intensive care or step-down unit. Again, this is a common event among women who use substances, including opioids and medication for OUD.

Women may live in poverty or confront violence. Sadly, this is not uncommon in this population so events such as assault, even if it culminates in hospitalization, would not be considered a severe AE related to the study conditions.

Women with OUD commonly have comorbid problems with conditions such as depression and post-traumatic stress disorder. The presence of these conditions or symptoms of these conditions would not be attributable to this trial given that they are common outside of the trial and nothing in the trial promotes the onset or maintenance of these problems.

An example of an event related to the trial would be that the participant is upset by the questions that are asked of the participant. In this instance, every attempt will be made to discuss this with the patient and provide care and treatment for their distress related to this.

Another example of an event related to the trial would be a breach of confidentiality. Although we will add measures to minimize this risk, should it occur we will take corrective measures with the study team member who is responsible and/or modify systems to decrease the likelihood of a breach.

8.3.4 TIME PERIOD AND FREQUENCY FOR EVENT ASSESSMENT AND FOLLOW-UP

The occurrence of an adverse event (AE) or serious adverse event (SAE) may come to the attention of study personnel during study visits and interviews of a study participant presenting for medical care, or upon review by a study monitor.

All AEs, not otherwise precluded per the protocol, will be captured on the appropriate case report form (CRF). Information to be collected includes event description, time of onset, clinician's assessment of severity, relationship to study procedures (assessed only by those with the training and authority to make a diagnosis), and time of resolution/stabilization of the event. All AEs occurring while on study will be documented appropriately regardless of relationship. All AEs will be followed to adequate resolution.

Any medical or psychiatric condition that is present at the time that the participant is screened will be considered as baseline and not reported as an AE. However, if the study participant's condition deteriorates at any time during the study, it will be recorded as an AE.

At each study visit, the investigator will inquire about the occurrence of AE/SAEs since the last visit.

8.3.5 ADVERSE EVENT REPORTING

APPROVED BY THE YALE UNIVERSITY IRB 12/9/2020

AEs will be recorded as part of the participants study record and entered into the data capture system. They will be reported to the Data and Safety Monitoring Board (DSMB)

8.3.6 SERIOUS ADVERSE EVENT REPORTING

All SAEs will be reported to Dr. Forray (at Yale) or Drs. Yonkers or Byatt (at UMass) for evaluation. Local IRBs will be notified by the site investigator according to local policy. They will be reported to the Data and Safety Monitoring Board (DSMB)

8.3.7 EVENTS OF SPECIAL INTEREST

N/A

8.4 UNANTICIPATED PROBLEMS

8.4.1 DEFINITION OF UNANTICIPATED PROBLEMS

This protocol uses the definition of Unanticipated Problems as defined by the Office for Human Research Protections (OHRP). OHRP considers unanticipated problems involving risks to participants or others to include, in general, any incident, experience, or outcome that meets **all** of the following criteria:

- Unexpected in terms of nature, severity, or frequency given (a) the research procedures that are described in the protocol-related documents, such as the Institutional Review Board (IRB)-approved research protocol and informed consent document; and (b) the characteristics of the participant population being studied;
- Related or possibly related to participation in the research ("possibly related" means there is a reasonable possibility that the incident, experience, or outcome may have been caused by the procedures involved in the research); and
- Suggests that the research places participants or others at a greater risk of harm (including physical, psychological, economic, or social harm) than was previously known or recognized.]

8.4.2 UNANTICIPATED PROBLEMS REPORTING

The investigator will report unanticipated problems (UPs) to the reviewing Institutional Review Board (IRB) according to local policy and to Drs. Yonkers, Forray and Byatt. The UP report will include the following information:

- Protocol identifying information: protocol title and number, PI's name, and the IRB project number
- A detailed description of the event, incident, experience, or outcome
- An explanation of the basis for determining that the event, incident, experience, or outcome represents an UP
- A description of any changes to the protocol or other corrective actions that have been taken or are proposed in response to the UP

To satisfy the requirement for prompt reporting, UPs will be reported to Drs. Yonkers, Forray and Byatt within 24 hours of becoming aware of the event. This will be compiled and provided to the DSMB at regularly scheduled meetings or within 24 hours if they are serious AEs.

9 STATISTICAL CONSIDERATIONS

9.1 STATISTICAL HYPOTHESES

Primary Hypotheses:

H_1: Participants with an OUD are more likely to be *engaged in MAT and/or non-pharmacological care for an OUD* if the center is randomized to the CC vs the ECHO model of support.

H_2: Participants with an OUD treated in CC centers are more likely to be *retained in MAT and/or non-pharmacological care for an OUD* than those treated in a center randomized to the ECHO model of care.

H_3: Participants in the CC, compared to the ECHO condition, are more likely to increase at least one PAM level from baseline to delivery and 3-months post-partum.

Secondary Hypotheses:

H_1: Participants with an OUD are more likely to be *engaged in MAT* if the center is randomized to the CC vs the ECHO model of support.

H_2: Participants with an OUD treated in CC centers are more likely to be *retained in MAT* for an OUD than those treated in a center randomized to the ECHO model of care.

H_3: Babies born to mothers treated in CC centers will have higher birth weights on average compared to babies born to mothers treated in ECHO centers.

$H_{4:}$ Obstetrical providers randomized to the CC compared to the ECHO condition will score higher on subscales (autonomy, relationship with patients, patient care issues and relationship with staff) of the Physicians Worklife Survey.

9.2 SAMPLE SIZE DETERMINATION

We plan to recruit a sample of 6 matched pair clusters (total of 12 clusters), with 6 clusters per condition and 40 patients per cluster for a total of 480 patients. Hypothesizing that CC will perform better than ECHO, this sample size achieves 80% power to detect a clinically meaningful minimum difference of 0.20 between the CC proportion (0.94) and the ECHO proportion (0.74).

To estimate the sample size needed to test differences between models of care in the proportion of women retained in treatment at delivery using a permutation test, we used the method of Hayes and Bennett (1999) for matched-pair cluster randomized trials. Our null hypothesis is $p_{ECHO} = p_{CC}$ and the alternate hypothesis is $p_{ECHO} \neq p_{CC}$, where p is the proportion of women retained in treatment at delivery, setting a two-sided type 1 error rate of 0.05. We based the estimate of the proportion in the CC on a study by our co-investigator in which 94% of patients treated under a CC model continued MAT until delivery.

We could not find empirical data for an estimate of the coefficient of variation, so we followed the approach of Hayes and Bennett (1999). We generated 10,000 data sets with 6 clusters each, with true proportions assumed uniformly distributed between 0.72 and 0.99 to allow for a relatively wide range of plausible retention proportion values. In each of the simulated data sets coefficient of variation values

were calculated based on formula (9) in the manuscript. This follows the conservative approach suggested on p.322 of the paper (i.e. to use the coefficient of variation between clusters within each group as an upper limit for the coefficient of variation in matched pairs designs). After examining the distribution of the resulting coefficient of variation we selected 0.10 as the value for our subsequent power calculations since more than 90% of the data sets had values under that threshold.

In an ongoing study of mothers with depression randomized to cognitive behavioral therapy with or without a community ambassador, 37% of 103 participants increased by at least one PAM level.(M. Smith, 2019, personal communication) Using this proportion for the CC group, 40 participants per site will provide 80% power to detect a 15% difference in the proportion of women who increase at least one PAM level between CC and ECHO. These estimates assume a two-sided test, with a significance level of 0.05 and an estimated within-pair coefficient of variation between clusters (CV) of 0.10.

For minimum detectable effect sizes for secondary outcomes at 80% power (assuming two-sided alpha = 0.05, 6 CC sites, 6 Echo sites, 40 participants per group, CV=0.1) we generated the table below.

**Table 2**<br>Continuous Outcomes	Mean (std. dev.) in Collaborative Care Group	Minimum detectable difference	Effect size (Cohen's d)
Birth weight	3137 (629)	597	0.95
Dichotomous Outcomes	Proportion for Collaborative Care Group	Minimum detectable difference	Effect size (Risk Ratio ECHO:CC)
Retained on MAT	0.89	0.69	0.78

We were not able to assess power for the remaining secondary outcomes: physician work-life survey and engagement with MAT given the limited prior research on these topics.

9.3 POPULATIONS FOR ANALYSES

Primary analyses will use an intention-to-treat analysis population. If missing data are a significant issue, we will use sensitivity analyses and/or multiple imputation methods, depending upon the nature of the missing data. Datasets for sensitivity analyses include per-protocol, complete cases, missing imputed as positive outcome (e.g. retained in treatment) and/or missing imputed as negative outcome (e.g. not retained in treatment). Multiply imputed data sets will be based upon multiple imputation methods that are appropriate for clustered data and the quantity, type (missing completely at random, missing at random, not missing at random), and pattern (monotone, or non-monotone) of missing data. Our safety analysis data sets will include all enrolled patients.

9.4 STATISTICAL ANALYSES

9.4.1 GENERAL APPROACH

We will follow CONSORT guidelines, modified for cluster randomized trials, for reporting the trial (www.consort-statement.org)(IR-5). Plans for data analysis will follow the major aims articulated above (IR-1) and the matched-pain cluster-randomized study design. We will examine the distribution of variables through descriptive statistics and graphical tools to verify that model assumptions are met to assess relationships between variables. Data analyses will use the intent-to-treat principle and we will attempt to follow all participants regardless of their retention to treatment. Dropouts (MD-3) and completers will be compared on baseline characteristics. Missingness (MD-2,4) will be examined for quantity, type (missing completely at random versus missing at random or not missing at random), and pattern (monotone, or

non-monotone). If missing data are a significant issue, we will use sensitivity analyses (MD-4) and multiple imputation methods that are appropriate for clustered data to fill in missing values in order to minimize bias, maximize available information and get accurate estimates of uncertainty. The possible impact of missing data on interpretation will be discussed in reports.

9.4.2 ANALYSIS OF THE PRIMARY ENDPOINT(S)

We have three primary outcomes that are all dichotomous: engagement in OUD treatment, retention in treatment, and an increase of at least one PAM level from baseline to delivery and 3-months post-partum. To tests differences in these outcomes between care models, we will use permutation tests (99-101) to account for cluster randomization (rather than individual randomization), as well as randomization within matched cluster pairs. Permutation tests are non-parametric and are a type of exact test. To conduct a permutation test, the mean difference in proportions (or means) between observed matched pairs and the corresponding t-statistic are calculated. Under the null hypothesis in permutation tests, there are no differences in outcomes between care models, making model assignment exchangeable (equivalent). The observed model assignment is only one of 64 equally likely possible ways of assigning models within pairs (2^n, where n= number of matched pairs; in our study n=6). The next step is to calculate mean differences and t-statistics for the remaining 63 assignment possibilities to serve as a null distribution. The t-statistics from permuted pairs are compared with the observed t-statistic and a p-value is calculated as the proportion of t-statistics that are larger in absolute value than the absolute value of the t-statistic from the observed data.

9.4.3 ANALYSIS OF THE SECONDARY ENDPOINT(S)

To tests differences in secondary patient outcomes between care models groups, we will use permutation tests as described above.

Our co-Investigator, Dr. Kaufman will collect and analyze qualitative data. Qualitative interviews will be will be audio recorded, transcribed, and independently coded by two trained masters level researchers who will be supervised by Dr. Kaufman. All transcripts will be de-identified. The team will develop codes to be used in the content analysis(102, 103) and utilize these codes to identify themes related to deployment of the two models. Themes will be examined both within and across informant groups. The team will use NVivo (104) to analyze data. Subsequent to the initial training, two staff will independently code each transcript and meet together with Dr. Kaufman to compare codes, discuss and work towards consensus in order to build a common understanding and use of codes.(105) Dr. Kaufman will review interrater reliability on a regular basis in order to prevent coder drift and will retrain staff as needed. The collection of data from multiple informants, iterative process of data collection and analysis and use of two researchers to code transcripts, increases creditability, transferability, dependability, and confirmability of the findings.(105, 106)

9.4.4 BASELINE DESCRIPTIVE STATISTICS

Differences between care models in baseline demographic and clinical characteristics will be assessed within matched pairs and overall using chi-square tests for categorical variables and t-tests for continuous variables, or their non-parametric counterparts if the assumption of normality is not met.

9.4.5 PLANNED INTERIM ANALYSES

N/A

9.4.6 SUB-GROUP ANALYSES

To evaluate heterogeneity of effects we will report estimates of proportion and mean differences within each matched pair together with 95% confidence intervals. Due to feasibility constraints (only 12 groups in 6 matched pairs) and lack of effect size estimates, we will not be able to perform a rigorous statistical evaluation of the effects of site level factors. However, we will present descriptive analyses of the sites that might be useful in guiding future use of the proposed models.

In addition, we will provide descriptive and exploratory analyses to evaluate heterogeneity of treatment effects on subject-level outcomes due to individual-level covariates including age, race/ethnicity, parity, illicit substances vs non-medical use of opioid prescriptions and concurrent substance use. We will use mixed effects statistical models to account for potential positive correlations among observations within sites (by including random site effects) and fixed effects of the intervention and potential covariates at the individual level. Interactions between treatment and potential moderating factors will be considered. Generalized linear mixed models will be used for categorical outcomes and linear mixed models for continuous outcomes.

9.4.7 TABULATION OF INDIVIDUAL PARTICIPANT DATA

Data will be analyzed at the site not individual level.

9.4.8 EXPLORATORY ANALYSES

Exploratory analyses will compare care models for eligible women (gravidas with an OUD, not in treatment when presenting for prenatal care) on the following outcomes: 1) #/% offered MAT including buprenorphine, specifically; and 2) rates of abstinence from illicit opioids or misuse of prescription opioids. (107) Additionally, for all pregnant women treated with MAT: 3) #/% continuing MAT at 3 months postpartum; 4) #/% engaging in an opioid treatment program at 3 months post-delivery. We will also explore differences between care models in: 5) #/% with concurrent substance use according to the **Time Line Follow-Back (TLFB)** (107) and urine tests; 6) fetal and neonatal outcomes (low birth weight, resuscitation at delivery, fetal demise, preterm birth, duration of hospitalization).

Exploratory analyses will also compare models in terms of participant reported differences in: 1) the **Shared-Decision Making Questionnaire-9** (**SDM-Q-9**) (108); 2) **Perceived efficacy in Patient-Physician Interactions** Questionnaire **(PEPPI)** (109); 3) Kim Alliance Scale-Communication subscale (**KAS-CM**) (110); 4) PROMIS Emotional Short Form(111) and Satisfaction with Roles and Activities(111); and 5) **Edinburgh Postnatal Depression Scale (EPDS)**(112, 113) .

We will explore racial and ethnic differences on patient measures and possible differences in outcomes among those who were users of illicit street opioids vs misusers of prescription opioids.

10 SUPPORTING DOCUMENTATION AND OPERATIONAL CONSIDERATIONS

10.1 REGULATORY, ETHICAL, AND STUDY OVERSIGHT CONSIDERATIONS

10.1.1 INFORMED CONSENT PROCESS

10.1.1.1 CONSENT/ASSENT AND OTHER INFORMATIONAL DOCUMENTS PROVIDED TO PARTICIPANTS

Consent forms describing in detail the study intervention, study procedures, and risks will be given to the participant and written documentation of informed consent will be completed prior to any research procedures taking place.

10.1.1.2 CONSENT PROCEDURES AND DOCUMENTATION

The DSP at each site will confirm informed consent. Participants will be allowed as much time a necessary to read, understand and ask questions about the study and research procedures. Consent will take place in a private room.

10.1.2 STUDY DISCONTINUATION AND CLOSURE

This study may be temporarily suspended or prematurely terminated if there is sufficient reasonable cause. Written notification, documenting the reason for study suspension or termination, will be provided by the suspending or terminating party to study participants, investigator, funding agency, and regulatory authorities. Study participants will be contacted, as applicable, and be informed of changes to study visit schedule.

Circumstances that may warrant termination or suspension include, but are not limited to:

- Determination of unexpected, significant, or unacceptable risk to participants
- Demonstration of efficacy that would warrant stopping
- Insufficient compliance of study staff to the protocol (i.e., significant protocol violations)
- Data that are not sufficiently complete and/or evaluable
- Determination that the primary endpoint has been met
- Determination of futility

The study may resume once concerns about safety, protocol compliance, and data quality are addressed, and satisfy the funding agency, sponsor, IRB, or other relevant regulatory or oversight bodies.

10.1.3 CONFIDENTIALITY AND PRIVACY

Participant confidentiality and privacy is strictly held in trust by the participating investigators, their staff, the safety and oversight monitor(s), and the sponsor(s) and funding agency. This confidentiality is extended to the data being collected as part of this study. Data that could be used to identify a specific study participant will be held in strict confidence within the research team. No personally-identifiable information from the study will be released to any unauthorized third party without prior written approval of the sponsor/funding agency.

All research activities will be conducted in as private a setting as possible.

The study participant's contact information will be securely stored at each clinical site for internal use during the study. At the end of the study, all records will continue to be kept in a secure location for as long a period as dictated by the reviewing IRB, Institutional policies, or sponsor/funding agency requirements.

Study participant research data, which are for purposes of statistical analysis and scientific reporting, will be transmitted to and stored at Yale University. Individual participants and their research data will be identified by a unique study identification number whenever possible. The study data entry and study management systems used by clinical sites and by Yale's research staff will be secured and password protected. At the end of the study, all study databases will be de-identified and archived at the Yale.

Certificate of Confidentiality
To further protect the privacy of study participants, the Secretary, Health and Human Services (HHS), has issued a Certificate of Confidentiality (CoC) to all researchers engaged in biomedical, behavioral, clinical or other human subjects research funded wholly or in part by the federal government. We will apply for a CoC given the nature of our population.

Safety Oversight
Because of the vulnerable population involved we feel this study will require a DSMB. We will recruit 4 members, including at least 2 individuals with clinical experience with the population and a statistician. None of the individuals will have a conflict of interest and we will ask them to commit to participate as a member for the duration of the project. We will draft a template for a charter that includes an overview of the study, the responsibilities of DSMB members, confidentiality requirements, review of safety data, DSMB meetings, DSMB reports and policies including reporting policies, i.e. minutes to IRBs, all sites, PCORI. We will ask the DSMB to modify as needed and accept the final charter. The DSMB will meet at least every 6 months but may conduct additional meetings if needed. The members will have access to de-identified data if needed. The DSMB will generate reports and send them to IRBs and sites but will only include de-identified or summarized data. The meetings will include an open period, closed period and executive committee period. We will include the type of adverse events and serious adverse events but we will invite DSMB members to ask about other elements they feel are appropriate.

10.1.4 CLINICAL MONITORING

N/A

10.1.5 QUALITY ASSURANCE AND QUALITY CONTROL

Should independent monitoring become necessary, the PI will provide direct access to all trial related sites, source data/documents, and reports for the purpose of monitoring and auditing by the sponsor/funding agency, and inspection by local and regulatory authorities.]

Each site will perform internal quality management based on the SOPs to be provided by the Yale research staff regarding data and biological specimen collection, documentation and completion. All sites will follow a common quality management plan under the supervision of the site investigator and the research team at Yale.

Quality control (QC) procedures will be implemented as follows:

Informed consent --- The research team at Yale in conjunction with the site investigators will review both the documentation of the consenting process as well as a percentage of the completed consent documents. This review will evaluate compliance with GCP, accuracy, and completeness. Feedback will be provided to the study team to ensure proper consenting procedures are followed.

Source documents and the electronic data --- We will monitor data quality by random inspection of the completed forms (e.g. TLFB and others) by the Yale project manager. Problems will be discussed by the site investigators. If necessary, we will re-train data collectors.
The data manager will update data files monthly so that she can correct errors and so that we can monitor consequential differences between models. Data cleaning, which is conducted on a quarterly basis while the study is ongoing to prevent missing data, will include checking for accuracy of key

variables such as age, race, verification of missing data and non-response codes; examination of outliers; checks within and across files for logical inconsistencies. We will code all data by subject number only and will be locked in research offices. Access to REDCAP is password-protected. We will keep data confidential and will not release protected health information to the public. Data will be available to research staff, DSMB, the local IRB and government agencies as required. After study completion, we may provide data to other researchers after a written request and after any possible identifying information is purged. We will follow all disclosure rules pertaining to HIPAA in the exchange of any study data with other researchers. We will not identify subjects by name in any report of the study and a request will be made to NICHD/NIDA for a Certificate of Confidentiality.

Intervention Fidelity — Consistent delivery of the study interventions will be monitored throughout the intervention phase of the study by the Weitzman Institute (for ECHO) and by Dr. Ian Bennet for the CC condition. Procedures for ensuring fidelity of intervention delivery are described in **Section 6.2.1, Interventionist Training and Tracking**.

10.1.6 DATA HANDLING AND RECORD KEEPING

10.1.6.1 DATA COLLECTION AND MANAGEMENT RESPONSIBILITIES

Data collection will be the responsibility of the clinical trial staff at the site under the supervision of the site investigator and the research team at Yale. The investigator will be responsible for ensuring the accuracy, completeness, legibility, and timeliness of the data reported.

All source documents will be completed in a neat, legible manner to ensure accurate interpretation of data.

Hardcopies of the study visit worksheets will be provided for use as source document worksheets for recording data for each participant consented/enrolled in the study when applicable. Data recorded in the electronic case report form (eCRF) derived from source documents will be consistent with the data recorded on the source documents.

Clinical data will be collected using REDCAP>, a 21 CFR Part 11-compliant data capture system provided by Yale. The data system includes password protection and internal quality checks, such as automatic range checks, to identify data that appear inconsistent, incomplete, or inaccurate. Clinical data will be entered directly from the source documents or in some cases directly by the participant.

10.1.6.2 STUDY RECORDS RETENTION

Study documents will be retained for a minimum of 3 years. These documents should be retained for a longer period, however, if required by local regulations. No records will be destroyed without the written consent of Dr. Yonkers. It is the responsibility of Dr. Yonkers to inform the investigators when these documents no longer need to be retained.

10.1.7 PROTOCOL DEVIATIONS

This protocol defines a protocol deviation as any noncompliance with the clinical trial protocol. The noncompliance may be either on the part of the participant, the investigator, or the study site staff. As a result of deviations, corrective actions will be developed by the site and implemented promptly.

These practices are consistent with ICH GCP:

- Section 4.5 Compliance with Protocol, subsections 4.5.1, 4.5.2, and 4.5.3

APPROVED BY THE YALE UNIVERSITY IRB 12/9/2020

- Section 5.1 Quality Assurance and Quality Control, subsection 5.1.1
- Section 5.20 Noncompliance, subsections 5.20.1, and 5.20.2.

It will be the responsibility of the site investigator to use continuous vigilance to identify and report deviations. The site investigator will be responsible for knowing and adhering to the reviewing IRB reporting requirements.

APPROVED BY THE YALE UNIVERSITY IRB 12/9/2020

APPROVED BY THE YALE UNIVERSITY IRB 12/9/2020

11 REFERENCES

1. Haight SC, Ko JY, Tong VT, Bohm MK, Callaghan WM. Opioid Use Disorder Documented at Delivery Hospitalization — United States, 1999–2014. Morbidity and Mortality Weekly Report. 2018;67(31):845-9. doi: 10.15585/mmwr.mm6731a1. PubMed PMID: PMC6089335.
2. Patrick SW, Buntin MB, Martin PR, Scott TA, Dupont W, Richards M, Cooper WO. Barriers to Accessing Treatment for Pregnant Women with Opioid Use Disorder in Appalachian States. Substance abuse : official publication of the Association for Medical Education and Research in Substance Abuse. 2018:1-18. Epub 2018/06/28. doi: 10.1080/08897077.2018.1488336. PubMed PMID: 29949454.
3. LaBelle CT, Han SC, Bergeron A, Samet JH. Office-Based Opioid Treatment with Buprenorphine (OBOT-B): Statewide Implementation of the Massachusetts Collaborative Care Model in Community Health Centers. J Subst Abuse Treat. 2016;60:6-13. Epub 2015/08/04. doi: 10.1016/j.jsat.2015.06.010. PubMed PMID: 26233698; PMCID: PMC4682362.
4. Arora S, Thornton K, Jenkusky SM, Parish B, Scaletti JV. Project ECHO: Linking university specialists with rural and prison-based clinicians to improve care for people with chronic hepatitis C in New Mexico. Public Health Reports. 2007;122(SUPPL. 2):74-7.
5. Hand DJ, Short VL, Abatemarco DJ. Treatments for opioid use disorder among pregnant and reproductive-aged women. Fertil Steril. 2017;108(2):222-7. doi: *http://dx.doi.org/10.1016/j.fertnstert.2017.06.011*.
6. Compton WM, Jones CM, Baldwin GT. Relationship between Nonmedical Prescription-Opioid Use and Heroin Use. N Engl J Med. 2016;374(2):154-63. doi: 10.1056/NEJMra1508490.
7. Rudd R, Aleshire N, Zibbell J, Gladden R. Inreases in drug and opioid overdose deaths US 2000-2014. Morbidity and Mortality Weekly Report. 2016;64(51):1378-82.
8. Kozhimannil KB, Graves AJ, Levy R, Patrick SW. Nonmedical Use of Prescription Opioids among Pregnant U.S. Women. Women's Health Issues. 2017;27(3):308-15. doi: *https://doi.org/10.1016/j.whi.2017.03.001*.
9. Bateman BT, Hernandez-Diaz S, Rathmell JP, Seeger JD, Doherty M, Fischer MA, Huybrechts KF. Patterns of opioid utilization in pregnancy in a large cohort of commercial insurance beneficiaries in the United States. Anesthesiology. 2014;120(5):1216-24. Epub 2014/02/15. doi: 10.1097/aln.0000000000000172. PubMed PMID: 24525628; PMCID: Pmc3999216.
10. Reddy UM, Davis JM, Ren Z, Greene MF. Opioid Use in Pregnancy, Neonatal Abstinence Syndrome, and Childhood Outcomes: Executive Summary of a Joint Workshop by the Eunice Kennedy Shriver National Institute of Child Health and Human Development, American College of Obstetricians and Gynecologists, American Academy of Pediatrics, Society for Maternal-Fetal Medicine, Centers for Disease Control and Prevention, and the March of Dimes Foundation. Obstetrics & Gynecology. 2017;130(1):10-28. doi: 10.1097/aog.0000000000002054. PubMed PMID: 00006250-201707000-00004.
11. Patrick SW, Davis MM, Lehmann CU, Cooper WO. Increasing incidence and geographic distribution of neonatal abstinence syndrome: United States 2009 to 2012. J Perinatol. 2015;35(8):650-5. Epub 2015/05/01. doi: 10.1038/jp.2015.36. PubMed PMID: 25927272; PMCID: PMC4520760.
12. Maeda A, Bateman BT, Clancy CR, Creanga AA, Leffert LR. Opioid abuse and dependence during pregnancy: temporal trends and obstetrical outcomes. Anesthesiology. 2014;121(6):1158-65. Epub 2014/11/19. doi: 10.1097/aln.0000000000000472. PubMed PMID: 25405293.
13. Smith MV, Costello D, Yonkers KA. Clinical correlates of prescription opioid analgesic use in pregnancy. Matern Child Health J. 2015;19(3):548-56. Epub 2014/06/22. doi: 10.1007/s10995-014-1536-6. PubMed PMID: 24951127; PMCID: Pmc4272915.
14. Salihu HM, Salemi JL, Aggarwal A, Steele BF, Pepper RC, Mogos MF, Aliyu MH. Opioid Drug Use and Acute Cardiac Events Among Pregnant Women in the United States. Am J Med. 2018;131(1):64-71 e1. Epub 2017/08/16. doi: 10.1016/j.amjmed.2017.07.023. PubMed PMID: 28807713.

APPROVED BY THE YALE UNIVERSITY IRB 12/9/2020

APPROVED BY THE YALE UNIVERSITY IRB 12/9/2020

15. Jumah NA, Edwards C, Balfour-Boehm J, Loewen K, Dooley J, Gerber Finn L, Kelly L. Observational study of the safety of buprenorphine+naloxone in pregnancy in a rural and remote population. BMJ Open. 2016;6(10):e011774. Epub 2016/11/02. doi: 10.1136/bmjopen-2016-011774. PubMed PMID: 27799240; PMCID: PMC5093362.
16. Jumah NA, Graves L, Kahan M. The management of opioid dependence during pregnancy in rural and remote settings. CMAJ. 2015;187(1):E41-6. Epub 2014/10/08. doi: 10.1503/cmaj.131723. PubMed PMID: 25288311; PMCID: PMC4284191.
17. Meyer M, Phillips J. Caring for pregnant opioid abusers in Vermont: A potential model for non-urban areas. Preventive Medicine. 2015;80:18-22. doi: *http://dx.doi.org/10.1016/j.ypmed.2015.07.015*.
18. Saia K, Bagley SM, Wachman EM, Patel PP, Nadas MD, Brogly SB. Prenatal treatment for opioid dependency: observations from a large inner-city clinic. Addict Sci Clin Pract. 2017;12(1):5. Epub 2017/01/15. doi: 10.1186/s13722-016-0070-9. PubMed PMID: 28086944; PMCID: PMC5237261.
19. Latt NC, Spencer JD, Beeby PJ, M GW, Saunders JB, Collins E, Cossart YE. Hepatitis C in injecting drug-using women during and after pregnancy. Journal of Gastroenterology and Hepatology. 2001;15(2):175-81. doi: 10.1046/j.1440-1746.2000.02060.x.
20. Brogly SB, Saia KE, Werler MM, Regan E, Hernandez-Diaz S. Prenatal Treatment and Outcomes of Women With Opioid Use Disorder. Obstet Gynecol. 2018. Epub 2018/09/12. doi: 10.1097/aog.0000000000002881. PubMed PMID: 30204704.
21. Broz D, Wejnert C, Pham HT, DiNenno E, Heffelfinger JD, Cribbin M, Krishna N, Teshale EH, Paz-Bailey G, National HIVBSSSG. HIV Infection and Risk, Prevention, and Testing Behaviors Among Injecting Drug Users — National HIV Behavioral Surveillance System, 20 U.S. Cities, 2009. Morbidity and Mortality Weekly Report: Surveillance Summaries. 2014;63(6):1-51.
22. Yonkers K, Gilstad-Hayden K, Forray A, Lipkind HS. Association of panic disorder, generalized anxiety disorder, and benzodiazepine treatment during pregnancy with risk of adverse birth outcomes. JAMA Psychiatry. 2017. doi: 10.1001/jamapsychiatry.2017.2733.
23. Gouin K, Murphy K, Shah PS. Effects of cocaine use during pregnancy on low birthweight and preterm birth: systematic review and metaanalyses. Am J Obstet Gynecol. 2011;204(4):340.e1-12. Epub 2011/01/25. doi: 10.1016/j.ajog.2010.11.013. PubMed PMID: 21257143.
24. Quesada O, Gotman N, Howell HB, Funai EF, Rounsaville BJ, Yonkers KA. Prenatal hazardous substance use and adverse birth outcomes. The journal of maternal-fetal & neonatal medicine : the official journal of the European Association of Perinatal Medicine, the Federation of Asia and Oceania Perinatal Societies, the International Society of Perinatal Obstet. 2012;25(8):1222-7. Epub 2012/04/12. doi: 10.3109/14767058.2011.602143. PubMed PMID: 22489543; PMCID: Pmc3398231.
25. Cleary BJ, Eogan M, O'Connell MP, Fahey T, Gallagher PJ, Clarke T, White MJ, McDermott C, O'Sullivan A, Carmody D, Gleeson J, Murphy DJ. Methadone and perinatal outcomes: a prospective cohort study. Addiction. 2012;107(8):1482-92. doi: 10.1111/j.1360-0443.2012.03844.x.
26. Martin CE. Recent trends in treatment admissions for prescription opioid abuse during pregnancy. Journal of substance abuse treatment. 2015;48(1):37-42. doi: 10.1016/j.jsat.2014.07.007.
27. Jarlenski M, Barry CL, Gollust S, Graves AJ, Kennedy-Hendricks A, Kozhimannil K. Polysubstance Use Among US Women of Reproductive Age Who Use Opioids for Nonmedical Reasons. Am J Public Health. 2017;107(8):1308-10. Epub 2017/06/24. doi: 10.2105/ajph.2017.303825. PubMed PMID: 28640680; PMCID: PMC5508143.
28. Eggleston AM, Calhoun PS, Svikis DS, Tuten M, Chisolm MS, Jones HE. Suicidality, aggression, and other treatment considerations among pregnant, substance-dependent women with posttraumatic stress disorder. Comprehensive Psychiatry. 2009;50(5):415-23. PubMed PMID: 19683611.
29. Jones HE, Heil SH, Tuten M, Chisolm MS, Foster JM, O'Grady KE, Kaltenbach K. Cigarette smoking in opioid-dependent pregnant women: neonatal and maternal outcomes. Drug Alcohol Depend.

APPROVED BY THE YALE UNIVERSITY IRB 12/9/2020

2013;131(3):271-7. Epub 2013/01/03. doi: 10.1016/j.drugalcdep.2012.11.019. PubMed PMID: 23279924; PMCID: PMC3694998.
30. Goldner EM, Lusted A, Roerecke M, Rehm J, Fischer B. Prevalence of Axis-1 psychiatric (with focus on depression and anxiety) disorder and symptomatology among non-medical prescription opioid users in substance use treatment: Systematic review and meta-analyses. Addictive Behaviors. 2014;39(3):520-31. doi: *https://doi.org/10.1016/j.addbeh.2013.11.022*.
31. SAMHSA. Medications for Opioid Use Disorder. US Department of Health and Human Services, 2018 Contract No.: HHs Publication No. (SMA) 18-5063.
32. Kampman K, Comer S, Cunningham CO, Fishman MJ, Gordon A, Langleben D, Nordstrom B, Oslin D, Woody G, Wright T, Wyatt S. National Practice Guideline for the Use of Medications in the Treatment of Addiction Involving Opioid Use. Chevy Chase, Md
American Society of Addiction Medicine, 2015.
33. ACOG. Opioid use and opioid use disorder in pregnancy. Committee Opinion No.711. Obstet Gynecol. 2017;130:e81-94.
34. SAMHSA. A collaborative approach to the treatment of pregnant women with opioid use disorder. Rockville, MD: Substance Abuse and Mental Health Services Administration, 2016.
35. Jones HE. Treating opioid use disorders during pregnancy: historical, current, and future directions. Substance abuse : official publication of the Association for Medical Education and Research in Substance Abuse. 2013;34(2):89-91. Epub 2013/04/13. doi: 10.1080/08897077.2012.752779. PubMed PMID: 23577898.
36. Klaman SL, Isaacs K, Leopold A, Perpich J, Hayashi S, Vender J, Campopiano M, Jones HE. Treating Women Who Are Pregnant and Parenting for Opioid Use Disorder and the Concurrent Care of Their Infants and Children: Literature Review to Support National Guidance. J Addict Med. 2017;11(3):178-90. Epub 2017/04/14. doi: 10.1097/adm.0000000000000308. PubMed PMID: 28406856; PMCID: PMC5457836.
37. Fullerton CA, Kim M, Thomas CP, Lyman DR, Montejano LB, Dougherty RH, Daniels AS, Ghose SS, Delphin-Rittmon ME. Medication-assisted treatment with methadone: assessing the evidence. Psychiatr Serv. 2014;65(2):146-57. Epub 2013/11/20. doi: 10.1176/appi.ps.201300235. PubMed PMID: 24248468.
38. Jones HE, O'Grady KE, Malfi D, Tuten M. Methadone Maintenance vs. Methadone Taper During Pregnancy: Maternal and Neonatal Outcomes. The American Journal on Addictions. 2008;17(5):372-86. doi: 10.1080/10550490802266276.
39. Wilder C. Medication assisted treatment discontinuation in pregnant and postpartum women with opioid use disorder. Drug Alcohol Depend. 2015;149:225-31. doi: 10.1016/j.drugalcdep.2015.02.012.
40. Jones HE, Kaltenbach K, Heil SH, Stine SM, Coyle MG, Arria AM, O'Grady KE, Selby P, Martin PR, Fischer G. Neonatal abstinence syndrome after methadone or buprenorphine exposure. N Engl J Med. 2010;363(24):2320-31. Epub 2010/12/15. doi: 10.1056/NEJMoa1005359. PubMed PMID: 21142534; PMCID: PMC3073631.
41. McCarthy JJ. Opioid dependence and pregnancy: minimizing stress on the fetal brain. American journal of obstetrics and gynecology. 2017;216(3):226-31. doi: 10.1016/j.ajog.2016.10.003.
42. Jones HE, Terplan M, Meyer M. Medically Assisted Withdrawal (Detoxification): Considering the Mother-Infant Dyad. Journal of Addiction Medicine. 2017;11(2):90-2. PubMed PMID: 28079573.
43. Stewart RD, Nelson DB, Adhikari EH, McIntire DD, Roberts SW, Dashe JS, Sheffield JS. The obstetrical and neonatal impact of maternal opioid detoxification in pregnancy. American Journal of Obstetrics and Gynecology. 2013;209(3):267.e1-.e5. doi: *http://dx.doi.org/10.1016/j.ajog.2013.05.026*.
44. Dashe JS, Jackson GL, Olscher DA, Zane EH, Wendel GD, Jr. Opioid detoxification in pregnancy. Obstetrics & Gynecology. 1998;92(5):854-8. PubMed PMID: 9794682.

APPROVED BY THE YALE UNIVERSITY IRB 12/9/2020

45. Bell J, Towers CV, Hennessy MD, Heitzman C, Smith B, Chattin K. Detoxification from opiate drugs during pregnancy. American Journal of Obstetrics and Gynecology. 2016;215(3):374.e1-.e6. doi: *https://doi.org/10.1016/j.ajog.2016.03.015*.
46. Nechanská B, Mravčík V, Skurtveit S, Lund IO, Gabrhelík R, Engeland A, Handal M. Neonatal outcomes after fetal exposure to methadone and buprenorphine: national registry studies from the Czech Republic and Norway. Addiction. 2018;113(7):1286-94. doi: 10.1111/add.14192.
47. Caritis SN, Bastian JR, Zhang H, Kalluri H, English D, England M, Bobby S, Venkataramanan R. An evidence-based recommendation to increase the dosing frequency of buprenorphine during pregnancy. American Journal of Obstetrics & Gynecology. 2017;217(4):459.e1-.e6. PubMed PMID: 28669739.
48. Penchansky R, Thomas JW. The concept of access: definition and relationship to consumer satisfaction. Med Care. 1981;19(2):127-40. Epub 1981/02/01. PubMed PMID: 7206846.
49. Jackson A, Shannon L. Barriers to receiving substance abuse treatment among rural pregnant women in Kentucky. Matern Child Health J. 2012;16(9):1762-70. Epub 2011/12/06. doi: 10.1007/s10995-011-0923-5. PubMed PMID: 22139045.
50. Sutter MB. Patient-centered Care to Address Barriers for Pregnant Women with Opioid Dependence. Obstetrics and gynecology clinics of North America. 2017;44(1):95-107. doi: 10.1016/j.ogc.2016.11.004.
51. Bachhuber MA, Mehta PK, Faherty LJ, Saloner B. Medicaid Coverage of Methadone Maintenance and the Use of Opioid Agonist Therapy Among Pregnant Women in Specialty Treatment. Med Care. 2017;55(12):985-90. Epub 2017/11/15. doi: 10.1097/mlr.0000000000000803. PubMed PMID: 29135769.
52. Falletta L, Hamilton K, Fischbein R, Aultman J, Kinney B, Kenne D. Perceptions of child protective services among pregnant or recently pregnant, opioid-using women in substance abuse treatment. Child Abuse Negl. 2018;79:125-35. Epub 2018/02/13. doi: 10.1016/j.chiabu.2018.01.026. PubMed PMID: 29433069.
53. Kremer ME. Clinical, Ethical, and Legal Considerations in Pregnant Women With Opioid Abuse. Obstetrics and gynecology (New York 1953). 2015;126(3):474-8. doi: 10.1097/AOG.0000000000000991.
54. Guille C, Barth KS, Mateus J, McCauley JL, Brady KT. Treatment of Prescription Opioid Use Disorder in Pregnant Women. American Journal of Psychiatry. 2017;174(3):208-14. doi: 10.1176/appi.ajp.2016.16060710.
55. MacAfee L, Sawyer A, Terplan M. Trends in receipt of medication-assisted treatment for pregnant women with opioid use disorder in the United States. Drug Alcohol Depend. 2017;171(Supplement C):e125. doi: *https://doi.org/10.1016/j.drugalcdep.2016.08.347*.
56. Huhn AS, Dunn KE. Why aren't physicians prescribing more buprenorphine? J Subst Abuse Treat. 2017;78:1-7. Epub 2017/05/31. doi: 10.1016/j.jsat.2017.04.005. PubMed PMID: 28554597; PMCID: PMC5524453.
57. Barry DT, Irwin KS, Jones ES, Becker WC, Tetrault JM, Sullivan LE, Hansen H, O’Connor PG, Schottenfeld RS, Fiellin DA. Integrating Buprenorphine Treatment into Office-based Practice: a Qualitative Study. Journal of General Internal Medicine. 2009;24(2):218-25. doi: 10.1007/s11606-008-0881-9.
58. Gordon AJ, Kavanagh G, Krumm M, Ramgopal R, Paidisetty S, Aghevli M, Goodman F, Trafton J, Liberto J. Facilitators and barriers in implementing buprenorphine in the Veterans Health Administration. Psychology of Addictive Behaviors. 2011;25(2):215-24. doi: 10.1037/a0022776.
59. Walley AY, Alperen JK, Cheng DM, Botticelli M, Castro-Donlan C, Samet JH, Alford DP. Office-Based Management of Opioid Dependence with Buprenorphine: Clinical Practices and Barriers. J Gen Intern Med. 2008;23(9):1393-8. doi: 10.1007/s11606-008-0686-x.
60. DeFlavio JR, Rolin SA, Nordstrom BR, Kazal LA, Jr. Analysis of barriers to adoption of buprenorphine maintenance therapy by family physicians. Rural Remote Health. 2015;15:3019. Epub 2015/02/05. PubMed PMID: 25651434.

APPROVED BY THE YALE UNIVERSITY IRB 12/9/2020

APPROVED BY THE YALE UNIVERSITY IRB 12/9/2020

61. McMurphy S, Shea J, Switzer J, Turner BJ. Clinic-based Treatment for Opioid Dependence: A Qualitative Inquiry. American Journal of Health Behavior. 2006;30(5):544-54. doi: 10.5993/AJHB.30.5.11.
62. Hutchinson E, Catlin M, Andrilla CHA, Baldwin L-M, Rosenblatt RA. Barriers to Primary Care Physicians Prescribing Buprenorphine. The Annals of Family Medicine. 2014;12(2):128-33. doi: 10.1370/afm.1595.
63. Kermack A, Flannery M, Tofighi B, McNeely J, Lee JD. Buprenorphine prescribing practice trends and attitudes among New York providers. Journal of Substance Abuse Treatment. 2017;74:1-6. doi: 10.1016/j.jsat.2016.10.005.
64. McRae-Clark AL, Carter RE, Price KL, Baker NL, Thomas S, Saladin ME, Giarla K, Nicholas K, Brady KT. Stress- and cue-elicited craving and reactivity in marijuana-dependent individuals. Psychopharmacology. 2011;218(1):49-58; PMCID: 3209966.
65. Andraka-Christou B, Capone MJ. A qualitative study comparing physician-reported barriers to treating addiction using buprenorphine and extended-release naltrexone in U.S. office-based practices. International Journal of Drug Policy. 2018;54:9-17. doi: *https://doi.org/10.1016/j.drugpo.2017.11.021*.
66. Cummings SR, Ensrud K, Delmas PD, LaCroix AZ, Vukicevic S, Reid DM, Goldstein S, Sriram U, Lee A, Thompson J, Armstrong RA, Thompson DD, Powles T, Zanchetta J, Kendler D, Neven P, Eastell R. Lasofoxifene in postmenopausal women with osteoporosis. N Engl J Med.362(8):686-96. Epub 2010/02/26. doi: 362/8/686 [pii]
10.1056/NEJMoa0808692. PubMed PMID: 20181970.
67. Krans EE, Bobby S, England M, Gedekoh RH, Chang JC, Maguire B, Genday P, English DH. The Pregnancy Recovery Center: A women-centered treatment program for pregnant and postpartum women with opioid use disorder. Addict Behav. 2018;86:124-9. Epub 2018/06/10. doi: 10.1016/j.addbeh.2018.05.016. PubMed PMID: 29884421.
68. Mittal L, Suzuki J. Feasibility of collaborative care treatment of opioid use disorders with buprenorphine during pregnancy. Substance Abuse. 2017;38(3):261-4. doi: 10.1080/08897077.2015.1129525.
69. Saia KA, Schiff D, Wachman EM, Mehta P, Vilkins A, Sia M, Price J, Samura T, DeAngelis J, Jackson CV, Emmer SF, Shaw D, Bagley S. Caring for Pregnant Women with Opioid Use Disorder in the USA: Expanding and Improving Treatment. Curr Obstet Gynecol Rep. 2016;5:257-63. Epub 2016/08/27. doi: 10.1007/s13669-016-0168-9. PubMed PMID: 27563497; PMCID: PMC4981621.
70. Jones HE, Deppen K, Hudak ML, Leffert L, McClelland C, Sahin L, Starer J, Terplan M, Thorp JM, Jr., Walsh J, Creanga AA. Clinical care for opioid-using pregnant and postpartum women: the role of obstetric providers. Am J Obstet Gynecol. 2014;210(4):302-10. Epub 2013/10/15. doi: 10.1016/j.ajog.2013.10.010. PubMed PMID: 24120973.
71. Ecker J, Abuhamad A, Hill W, Bailit J, Bateman BT, Berghella V, Blake-Lamb T, Guille C, Landau R, Minkoff H, Prabhu M, Rosenthal E, Terplan M, Wright TE, Yonkers KA. Substance Use Disorders in Pregnancy: Clinical, Ethical, and Research Imperatives of the Opioid Epidemic: A report of a joint workshop of the Society for Maternal-Fetal Medicine, American College of Obstetricians and Gynecologists, and American Society of Addiction Medicine. Am J Obstet Gynecol. 2019. Epub 2019/04/01. doi: 10.1016/j.ajog.2019.03.022. PubMed PMID: 30928567.
72. Chou R, Korthuis P, Weimer M, Bougatsos C, Blazina I, Zakher B, Grusing S, Devine B, McCarty D. Medication Assisted Treatment Models of Care for Opioid Use Disorder in Primary Care Settings. Rockville, MD: 2016 Contract No.: AHRQ Publication No. 16(17)-EHC039-EF.
73. Katon W, Von Korff M, Lin E, Walker E, Simon GE, Bush T, Robinson P, Russo J. Collaborative management to achieve treatment guidelines. Impact on depression in primary care. Journal of American Medical Association. 1995;273:1026-31.

APPROVED BY THE YALE UNIVERSITY IRB 12/9/2020

APPROVED BY THE YALE UNIVERSITY IRB 12/9/2020

74. Gilbody S, Bower P, Fletcher J, Richards D, Sutton AJ. Collaborative care for depression: a cumulative meta-analysis and review of longer-term outcomes. Arch Intern Med. 2006;166(21):2314-21. Epub 2006/11/30. doi: 10.1001/archinte.166.21.2314. PubMed PMID: 17130383.
75. Thota AB, Sipe TA, Byard GJ, Zometa CS, Hahn RA, McKnight-Eily LR, Chapman DP, Abraido-Lanza AF, Pearson JL, Anderson CW, Gelenberg AJ, Hennessy KD, Duffy FF, Vernon-Smiley ME, Nease DE, Jr., Williams SP. Collaborative care to improve the management of depressive disorders: a community guide systematic review and meta-analysis. Am J Prev Med. 2012;42(5):525-38. Epub 2012/04/21. doi: 10.1016/j.amepre.2012.01.019. PubMed PMID: 22516495.
76. Melville JL, Reed SD, Russo J, Croicu CA, Ludman E, LaRocco-Cockburn A, Katon W. Improving care for depression in obstetrics and gynecology: a randomized controlled trial. Obstet Gynecol. 2014;123(6):1237-46. Epub 2014/05/09. doi: 10.1097/aog.0000000000000231. PubMed PMID: 24807320; PMCID: PMC4052378.
77. Bhat A, Grote NK, Russo J, Lohr MJ, Jung H, Rouse CE, Howell EC, Melville JL, Carson K, Katon W. Collaborative Care for Perinatal Depression Among Socioeconomically Disadvantaged Women: Adverse Neonatal Birth Events and Treatment Response. Psychiatr Serv. 2017;68(1):17-24. Epub 2016/10/04. doi: 10.1176/appi.ps.201600002. PubMed PMID: 27691376.
78. Huffman JC, Niazi SK, Rundell JR, Sharpe M, Katon WJ. Essential articles on collaborative care models for the treatment of psychiatric disorders in medical settings: a publication by the academy of psychosomatic medicine research and evidence-based practice committee. Psychosomatics. 2014;55(2):109-22. Epub 2013/12/29. doi: 10.1016/j.psym.2013.09.002. PubMed PMID: 24370112.
79. Coventry PA, Hudson JL, Kontopantelis E, Archer J, Richards DA, Gilbody S, Lovell K, Dickens C, Gask L, Waheed W, Bower P. Characteristics of effective collaborative care for treatment of depression: a systematic review and meta-regression of 74 randomised controlled trials. PLoS One. 2014;9(9):e108114. Epub 2014/09/30. doi: 10.1371/journal.pone.0108114. PubMed PMID: 25264616; PMCID: PMC4180075.
80. Alford DP, LaBelle CT, Kretsch N, et al. Collaborative care of opioid-addicted patients in primary care using buprenorphine: Five-year experience. Archives of Internal Medicine. 2011;171(5):425-31. doi: 10.1001/archinternmed.2010.541.
81. Suzuki J, Matthews ML, Brick D, Nguyen M-T, Jamison RN, Ellner AL, Tishler LW, Weiss RD. Implementation of a collaborative care management program with buprenorphine in primary care: A comparison between opioid-dependent patients and chronic pain patients using opioids non-medically. Journal of opioid management. 2014;10(3):159-68. doi: 10.5055/jom.2014.0204. PubMed PMID: PMC4085743.
82. Watkins KE, Ober AJ, Lamp K, et al. Collaborative care for opioid and alcohol use disorders in primary care: The summit randomized clinical trial. JAMA Internal Medicine. 2017. doi: 10.1001/jamainternmed.2017.3947.
83. Arora S, Thornton K, Komaromy M, Kalishman S, Katzman J, Duhigg D. Demonopolizing Medical Knowledge. Academic Medicine. 2014;89(1):30-2. doi: 10.1097/acm.0000000000000051. PubMed PMID: 00001888-201401000-00014.
84. Arora S, Geppert CM, Kalishman S, Dion D, Pullara F, Bjeletich B, Simpson G, Alverson DC, Moore LB, Kuhl D, Scaletti JV. Academic health center management of chronic diseases through knowledge networks: Project ECHO. Acad Med. 2007;82(2):154-60. Epub 2007/02/01. doi: 10.1097/ACM.0b013e31802d8f68. PubMed PMID: 17264693; PMCID: PMC3855463.
85. Arora S, Thornton K, Murata G, Deming P, Kalishman S, Dion D, Parish B, Burke T, Pak W, Dunkelberg J, Kistin M, Brown J, Jenkusky S, Komaromy M, Qualls C. Outcomes of treatment for hepatitis C virus infection by primary care providers. New England Journal of Medicine. 2011;364(23):2199-207. doi: 10.1056/NEJMoa1009370.

APPROVED BY THE YALE UNIVERSITY IRB 12/9/2020

APPROVED BY THE YALE UNIVERSITY IRB 12/9/2020

86. Zhou C, Crawford A, Serhal E, Kurdyak P, Sockalingam S. The Impact of Project ECHO on Participant and Patient Outcomes: A Systematic Review. Acad Med. 2016;91(10):1439-61. Epub 2016/08/05. doi: 10.1097/acm.0000000000001328. PubMed PMID: 27489018.
87. Miriam Komaromy, Judy Bartlett, Kathryn Manis, Sanjeev Arora. Enhanced Primary Care Treatment of Behavioral Disorders With ECHO Case-Based Learning. Psychiatric Services. 2017;68(9):873-5. doi: 10.1176/appi.ps.201600471. PubMed PMID: 28806893.
88. Moeckli J, Stewart KR, Ono S, Alexander B, Goss T, Maier M, Tien PC, Howren MB, Ohl ME. Mixed-Methods Study of Uptake of the Extension for Community Health Outcomes (ECHO) Telemedicine Model for Rural Veterans With HIV. J Rural Health. 2017;33(3):323-31. Epub 2016/08/25. doi: 10.1111/jrh.12200. PubMed PMID: 27557039.
89. Rich KM, Bia J, Altice FL, Feinberg J. Integrated Models of Care for Individuals with Opioid Use Disorder: How Do We Prevent HIV and HCV? Curr HIV/AIDS Rep. 2018;15(3):266-75. Epub 2018/05/19. doi: 10.1007/s11904-018-0396-x. PubMed PMID: 29774442; PMCID: PMC6003996.
90. Mazurek MO, Brown R, Curran A, Sohl K. ECHO Autism: A New Model for Training Primary Care Providers in Best-Practice Care for Children with Autism. Clinical Pediatrics. 2017;56(3):247-56. doi: 10.1177/0009922816648288.
91. Sockalingam S, Arena A, Serhal E, Mohri L, Alloo J, Crawford A. Building Provincial Mental Health Capacity in Primary Care: An Evaluation of a Project ECHO Mental Health Program. Acad Psychiatry. 2018;42(4):451-7. Epub 2017/06/09. doi: 10.1007/s40596-017-0735-z. PubMed PMID: 28593537.
92. Carey EP, Frank JW, Kerns RD, Michael Ho P, Kirsh SR. Implementation of telementoring for pain management in Veterans Health Administration: Spatial analysis. Journal of Rehabilitation Research and Development. 2016;53(1):147-56. doi: 10.1682/JRRD.2014.10.0247.
93. Katzman JG, Comerci G, Boyle JF, Duhigg D, Shelley B, Olivas C, Daitz B, Carroll C, Som D, Monette R, Kalishman S, Arora S. Innovative telementoring for pain management: Project ECHO pain. Journal of Continuing Education in the Health Professions. 2014;34(1):68-75. doi: 10.1002/chp.21210.
94. Colleran K, Harding E, Kipp BJ, Zurawski A, MacMillan B, Jelinkova L, Kalishman S, Dion D, Som D, Arora S. Building Capacity to Reduce Disparities in Diabetes:Training Community Health Workers Using an Integrated Distance Learning Model. The Diabetes Educator. 2012;38(3):386-96. doi: 10.1177/0145721712441523. PubMed PMID: 22491397.
95. Komaromy M, Duhigg D, Metcalf A, Carlson C, Kalishman S, Hayes L, Burke T, Thornton K, Arora S. Project ECHO (Extension for Community Healthcare Outcomes): A new model for educating primary care providers about treatment of substance use disorders. Subst Abus. 2016;37(1):20-4. Epub 2016/02/06. doi: 10.1080/08897077.2015.1129388. PubMed PMID: 26848803; PMCID: PMC4873719.
96. Huhn AS, Dunn KE. Why aren't physicians prescribing more buprenorphine? Journal of Substance Abuse Treatment. 2017;78:1-7. doi: 10.1016/j.jsat.2017.04.005.
97. Turner BJ, Laine C, Lin Y, Lynch K. Barriers and facilitators to primary care or human immunodeficiency virus clinics providing methadone or buprenorphine for the management of opioid dependence. Archives of Internal Medicine. 2005;165(15):1769-76. doi: 10.1001/archinte.165.15.1769.
98. Wilder C, Lewis D, Winhusen T. Medication assisted treatment discontinuation in pregnant and postpartum women with opioid use disorder. Drug Alcohol Depend. 2015;149:225-31. Epub 2015/03/05. doi: 10.1016/j.drugalcdep.2015.02.012. PubMed PMID: 25735465.
99. Edgington E. Randomization Tests. 2nd ed. New York, NY: Marcel Dekker; 1987.
100. Gail MH, Mark SD, Carroll RJ, Green SB, Pee D. On design considerations and randomizatjion-based inference for community intervention trials. Statistics in Medicine. 1996;15(11):1069-92. doi: 10.1002/(SICI)1097-0258(19960615)15:11<1069::AID-SIM220>3.0.CO;2-Q.
101. Feng Z, Diehr P, Peterson A, McLerran D. Selected Statistical Issues in Group Randomized Trials. Annual Review of Public Health. 2001;22(1):167-87. doi: 10.1146/annurev.publhealth.22.1.167.

APPROVED BY THE YALE UNIVERSITY IRB 12/9/2020

APPROVED BY THE YALE UNIVERSITY IRB 12/9/2020

102. Patton M. Qualitative Evaluation and Research Methods. 2nd Edition ed. Newbury Park, California: Sage; 1990.
103. Krippendorff K. Content Analysis: An Introduction to its Methodology. 3rd Edition ed. Thousand Oaks, CA: Sage; 2013.
104. Ltd QIP. NVivo qualiative data analysis software. Version 10 ed2012.
105. Patton M. Enhancing the quality and credibility of qualitative analysis. Health Services Research. 1999;34:1189-208.
106. Guba E, Lincoln Y. Handbook of Qualitative Research. Newbury Park, CA: Sage; 1994.
107. Fals-Stewart W, O'Farrell TJ, Freitas TT, McFarlin SK, Rutigliano P. The timeline followback reports of psychoactive substance use by drug-abusing patients: psychometric properties. J Consult Clin Psychol. 2000;68:134-44.
108. Alegria M, Chatterji P, Wells K, Z C, Chen C-n, Takeuchi D, Jackson J, Meng X. Disparity in depression treatment among racial and ethnic minority populations in the United States Psychiatric Services 2008;59(11):1264-72.
109. Maly RC, Frank JC, Marshall GN, DiMatteo MR, Reuben DB. Perceived efficacy in patient-physician interactions (PEPPI): validation of an instrument in older persons. J Am Geriatr Soc. 1998;46(7):889-94. Epub 1998/07/22. PubMed PMID: 9670878.
110. Kim SC, Boren D, Solem SL. The Kim Alliance Scale: Development and Preliminary Testing. Clinical Nursing Research. 2001;10(3):314-31. doi: 10.1177/c10n3r7.
111. Cella D, Yount S, Rothrock N, Gershon R, Cook K, Reeve B, Ader D, Fries JF, Bruce B, Rose M. The Patient-Reported Outcomes Measurement Information System (PROMIS): Progress of an NIH Roadmap Cooperative Group During its First Two Years. Medical care. 2007;45(5 Suppl 1):S3-S11. doi: 10.1097/01.mlr.0000258615.42478.55. PubMed PMID: PMC2829758.
112. Cox JL, Holden JM, Sagovsky R. Detection of postnatal depression: Development of the 10-item Edinburgh Postnatal Depression Scale. The British Journal of Psychiatry. 1987;150:782-6.
113. Cox JL, Chapman G, Murray D, Jones P. Validation of the Edinburgh Postnatal Depression Scale (EPDS) in non-postnatal women. J Affect Disord. 1996;Volume 39(Issue 3):185-9.

APPROVED BY THE YALE UNIVERSITY IRB 12/9/2020

APPROVED BY THE YALE UNIVERSITY IRB 12/9/2020

APPENDIX: STUDY PERSONNEL AND ROLES

Kimberly Ann Yonkers, M.D., Co-Principal Investigator

Dr. Kimberly Yonkers is a Professor in the Departments of Psychiatry and Obstetrics, Gynecology and Reproductive Sciences at the Yale School of Medicine. She is Chief of the Psychological Medicine Service and Director of Research for the Yale New Haven Hospital (YNHH) Division of Psychiatry Dr. Yonkers also has a history of continuous NIH funding for the past 20 years with an emphasis on treatment of substance use disorders. As co-Principal Investigator of this proposed project, Dr. Yonkers and Dr. Forray will oversee the overall conduct of the project. Dr. Yonkers will work on administrative components of the project, ensuring that human subjects approvals are completed, data collection forms and systems are designed comprehensively and with good functionality and that the budgets and contracts are accurately managed. She will also participate with other members of the team in designing the educational material for both sites. She will work with PCORI, the SAC and the PSG in making modifications to the final protocol. In terms of data collection and management systems, she will oversee the Project Manager and statisticians to design, test and implement the forms and databases that are needed for the extensive data collection. Dr. Yonkers will work with Dr. Forray and others in developing a publication plan and will work with the team to develop and submit manuscripts.

Ariadna Forray, M.D., Co-Principal Investigator

Dr. Forray is a reproductive psychiatrist and Associate Professor in the Department of Psychiatry. She is Co-Director of the Center for Wellness of Women and Mothers, a reproductive psychiatry research program. Dr. Forray has a background in women's mental health and addiction. As a principal investigator and co-investigator on several NIH-funded grants, she has developed and implemented addiction treatment interventions, successfully recruited pregnant women with substance use disorders for participation in clinical research and collaborated with researchers and clinicians across disciplines. As co-Principal Investigator of this proposed project, Dr. Yonkers and Dr. Forray will oversee the overall conduct of the project. Dr. Forray will be responsible for the field aspects of the study. With the other investigators, she will finalize the educational material for both groups, and will work to assemble the SAC and PSG and ensure that their input is integrated into the protocol. She will oversee and provide onsite supervision and training of sites in Connecticut. She will also work with the sites and the Project Manager to ensure that CT sites have the supplies and resources to conduct their tasks related to the trial, in addition to working with the Project Manager to plan and conduct staff meetings.

Nancy Byatt, D.O., MS, MBA, FACLP, UMass Site PI

Dr. Byatt is a perinatal and consultation-liaison psychiatrist, Associate Professor of Psychiatry, Ob/Gyn, and Quantitative Health Sciences, and expert in addressing perinatal mental health and substance use disorders in obstetric settings. She is the Medical Director of the population-based program, the Massachusetts Psychiatry Access Program for Moms (MCPAP for Moms), that aims to address perinatal

APPROVED BY THE YALE UNIVERSITY IRB 12/9/2020

depression throughout the state of MA by building the capacity of front line Ob/Gyn providers to address perinatal mental health and substance use disorders. Her program of research - developing and evaluating innovative interventions to improve mental health outcomes for perinatal women mental health and substance use disorders - is completely aligned with the objectives of this application. She is currently testing and intervention, the PRogram In Support of Moms (PRISM). It is a cluster randomized control trial (RCT) that compares a set of 6 Massachusetts practices using MCPAP for Moms telephone support to a set of 6 practices using MCPAP for Moms telephone support plus a stepped care approach.

Dr. Byatt will have ultimate managerial and administrative oversight for the Massachusetts sites and be the identified liaison with the Dr. Yonkers and the primary Yale study team. She will work in partnership with the PIs (Yonkers and Forray) and her Co-Is (Ms. Allen, Dr. Callaghan, Dr. Mittal, Dr. Lipkin, Dr. Fiellin) as well as the Study Advisory Group and the Patient Stakeholder Group in all aspects of the study. She will help ensure the integrity of the research, including developing materials for the IRB and working with UMMS and site-specific IRBs, and developing and executing participant recruitment strategies. She will help ensure data security and quality. She will participate on collaborative work with PCORI staff, patient stakeholders and the research group on the design, analyses and dissemination of study results, and will be involved all manuscript preparations. She will work closely with the study team and stakeholders on dissemination materials. She will participate in monthly conference calls with collaborators and provide progress updates.

David Fiellin, M.D., Co-Investigator

Dr. Fiellin is an Addiction Medicine trained internist at Yale, who served as national Director of Buprenorphine Education for the American Society of Addiction Medicine, was the first Medical Director of the SAMHSA-funded Physician Clinical Support System for Buprenorphine (PCSS-B), and currently serves as a special advisor to the Provider Clinical Support System for Medication Assisted Treatment (PCSS-MAT). He will provide the in-person Drug Addiction Treatment Act of 2000 (DATA 2000) buprenorphine waiver trainings. He will also help develop the educational materials on OUD and MAT for the CC and ECHO conditions.

<u>Katherine Callaghan, M.D.,</u> Co-Investigator

Dr. Callaghan is an Assistant Professor at University of Massachusetts Medical School. She is an Obstetrician-Gynecologist who was the founder of the Karen W Green Clinic for Pregnancy and Recovery. This program is embedded in the Community Women's Care program and provides both targeted obstetrical care and recovery services to pregnant and postpartum women in Central Massachusetts. The most recent year had 100 pregnant women with OUD. Dr. Callaghan was also the PI for a 3-year SAMHA grant awarded to Massachusetts Department of Public Health's Bureau of Substance Addiction Services in order to expand access to MAT for OUDs (MomsDoCare).

Heather Lipkind, M.D., Co-Investigator

Dr. Lipkind is an obstetrician/gynecologist at Yale with a background in perinatal epidemiology. She has collaborated with Drs. Yonkers and Forray work on examining the association of psychiatric conditions

and pregnancy outcomes. She will provide support on the use of buprenorphine in pregnancy for practices in CT. She will also work with Dr. Yonkers on data collection systems, and help develop the educational materials for the CC and ECHO conditions. She will work with the team in writing up results of findings.

Leena P. Mittal, M.D., FACLP, Co-Investigator

Dr. Mittal is Director of the Division of Women's Mental Health at the Brigham and Women's Hospital. Dr Mittal is an Instructor of Psychiatry at Harvard Medical School. She completed medical school at the University of Maryland School of Medicine and completed residency at the Harvard Longwood Psychiatry Residency Training Program. She also completed a Psychosomatic Medicine fellowship at Brigham and Women's Hospital. Dr Mittal is board certified in Psychiatry, Psychosomatic Medicine and Addiction Medicine. Dr Mittal is a Fellow of the Academy of Consultation Liaison Psychiatry. She has developed an expertise in the treatment of perinatal mental health and substance use disorders and has developed a hospital based program for the integrated treatment of opioid use disorder with buprenorphine during pregnancy with prenatal care. Dr Mittal has published a case series on collaborative care of opioid use disorders in pregnancy. Additionally, Dr Mittal is responsible for expansion of a large statewide perinatal mental health consultation service to include capacity to provide consultations regarding Substance Use Disorders for obstetric, psychiatric and primary care providers.

Joy Kaufman, Ph.D., Qualitative Analyst

Dr. Kaufman is a Professor of Psychiatry; Director of Program and Service System Evaluation at The Consultation Center; Director of Evaluation Research in the Division of Prevention and Community Research; and Director of Psychology Training at The Consultation Center. Dr. Kaufman's area of expertise is program evaluations, needs assessments, and qualitative and quantitative evaluations of service delivery systems. Accordingly, her role on this project will be to conduct, code, and analyze the qualitative interviews administered to the providers and subjects at the 12 sites. She will conduct interviews with providers and patients in years 1-3.

Ralitza Gueorguieva, Ph.D., Biostatistician

Dr. Gueorguieva is a Research Scientist in the Department of Epidemiology and Public Health at the Yale School of Medicine. Dr. Gueorguieva has extensive experience in the analysis of clustered and longitudinal data and has worked with Drs. Yonkers and Forray on various other projects. The goal of the proposal is to compare two conditions and is a cluster randomized trial. As such, the design of the study and the structure of data to be collected require that sophisticated statistical models are used for proper inference.

Kate Gilstad-Hayden, MS, Data Programmer/Manager

Ms. Gilstad-Hayden is an experienced data manager and statistician who has worked with the PIs of this application on previous projects. She is experienced managing large, complicated datasets, overseeing randomization of study subjects, and analyzing clinical and therapeutic outcomes. Ms. Gilstad-Hayden will be responsible for all data programming related to this project which will include programming data screens and tables for the study and checking the system to ensure functionality. She will regularly monitor the integrity of the data and supervise the Research Associate with regards to data entry and cleaning. Lastly, she will work closely with Dr. Gueorguieva to clean and convert the raw data into final data sets which will be analyzed. Her effort has been adjusted to coincide with the work load.

Karen Hunkele, Project Coordinator

Ms. Hunkele is a Research Associate in the Department of Psychiatry at the Yale University School of Medicine. The role on this project will be to serve as Project Coordinator, to supervise the Research Assistant, and to assist Dr. Yonkers and Dr. Forray in overseeing all aspects of data collection, coordination with other sites, recruitment of study subjects, and data entry and management. Ms. Hunkele has a 20-year history of overseeing research projects, including multi-center projects, at Yale School of Medicine.

Protocol Contributions

Drs. Yonkers and Forray conceived the study and are the grant holders. They are responsible for the study design and implementation.

Drs. Byatt, Callahan, Fiellin, Lipkind, Mittal, and Ms. Hunkele contributed to the study design and are assisted with implementation. Dr. Gueorguieva and Ms. Gilstad-Hayden provided statistical expertise, and Ms. Gilstad-Hayden is conducting the primary statistical analysis. Dr. Kaufman provided the qualitative interview design and will be responsible for the qualitative analysis. All personnel listed above contributed to refinement of the study protocol and approved the final version,
